# Direct observation of braiding of Majorana-like zero modes in real space

**DOI:** 10.1038/s41467-026-75765-4

**Published:** 2026-07-31

**Authors:** Qiyun Ma, Hailong He, Weiyin Deng, Meng Xiao, Zhengyou Liu

**Affiliations:** 1 Key Laboratory of Artificial Micro- and Nano-structures of Ministry of Education and School of Physics and Technology, Wuhan University, Wuhan, China; 2 Wuhan Institute of Quantum Technology, Wuhan, China; 3 Institute for Advanced Studies, Wuhan University, Wuhan, China

**Keywords:** Topological matter, Mechanical properties, Electronic devices

## Abstract

Majorana zero modes (MZMs) lie at the heart of fault-tolerant topological quantum computation, where their non-Abelian braiding statistics enable logical operations intrinsically immune to local perturbations and decoherence. Analogs of MZMs, known as Majorana-like zero modes (MLZMs), have recently been proposed in artificial lattices. Despite this progress, a direct real-space observation of the braiding process has remained elusive, representing a fundamental gap between theoretical prediction and experimental realization. Here, we demonstrate the direct observation of the braiding of MLZMs. We identify boundary-bound MLZMs (BMLZMs) that are spatially well separated and can be adiabatically transported along the sample boundary through controlled modulation of the Kekulé phase. By mapping the acoustic-field evolution, we construct the complete real-space trajectory and observe the phase accumulation characteristic of the braiding process. These results establish BMLZMs as a new class of topological excitations and provide a directly observable analog of Majorana braiding dynamics.

## Abstract (web summary)

Majorana-like zero modes are topological bound states whose exchange encodes non-trivial phase information in engineered systems. Here, the authors demonstrate boundary-localized modes in an acoustic Kekulé lattice and track their real-space evolution during braiding, revealing phase accumulation associated with their exchange.

## Introduction

Topological states of matter have transformed our understanding of physical systems by revealing that global invariants can protect localized excitations from disorder, perturbations, and environment-induced decoherence^1–3^. Among the most remarkable are Majorana zero modes (MZMs)^4–6^, whose non-Abelian exchange—or braiding—produces a unitary transformation of the system’s wavefunction, forming the foundation for topological quantum computation^7–9^. Tremendous efforts have been devoted to realizing MZMs in quantum materials^10–17^ such as semiconductor–superconductor heterostructures^12,13^, magnetic atom chains^14,15^, and proximitized topological insulators^16,17^. Although zero-energy signatures consistent with MZMs have been reported^18–20^, direct real-space observation of the wavefunctions and the braiding process remains unachieved, primarily because individual MZMs in electronic systems are difficult to manipulate and image.

In parallel, artificial topological lattices have emerged as versatile platforms for exploring non-Abelian and Majorana-like physics^21–29^. For instance, non-Abelian operations on multiple states have been demonstrated in acoustic and photonic waveguide systems^30–34^, as well as through state exchange induced by encircling multiple exceptional points^35–38^. On the other hand, vortex-textured Kekulé-modulated graphene structures possess effective Hamiltonians that share the same form as the Kitaev model^10^. They can host topological zero modes whose wavefunctions mirror those of MZMs bound to vortices in *p*-wave superconductors^7^. Originally proposed in electronic systems^39^, this correspondence has since inspired extensive research in photonic^21–24^, phononic^25,26^, and mechanical^27–29^ platforms, leading to Majorana-like zero modes (MLZMs) that reproduce the spatial field distribution and topological protection of their quantum counterparts^4–6^. Recent progress extends beyond analog demonstrations—MLZMs have also enabled practical functionalities such as optical vortex lasing^40,41^ and topological waveguiding^42,43^. Previous studies have realized MLZMs localized at vortex centers and reported interference signatures reminiscent of braiding^44–47^. However, these observations were indirect, relying on parameter-space modulation or static interference rather than tracking the real-space evolution of the MLZMs during the braiding process.

Here we report the direct observation of the braiding of MLZMs in real space, enabled by the discovery of boundary-bound Majorana-like zero modes (BMLZMs) in a vortex-textured Kekulé-modulated lattice. Unlike conventional vortex-center modes, these BMLZMs reside along the sample boundary and are spatially well separated. They can be continuously transported by tuning the modulation phase—features that make them ideally suited for braiding operations. By mapping the acoustic-field evolution layer by layer along the evolution direction, we directly visualize the complete trajectory and identify the characteristic phase accumulation that signifies non-Abelian braiding when multiple MLZMs are involved^44^. This real-space observation establishes BMLZMs as a new class of topological excitations and provides a fully observable analog of Majorana braiding dynamics. Beyond confirming a long-sought topological braiding process, our work introduces a powerful strategy for manipulating topological states and achieving robust information processing in both classical and quantum regimes.

## Results

### Lattice model

**Fig. 1:**
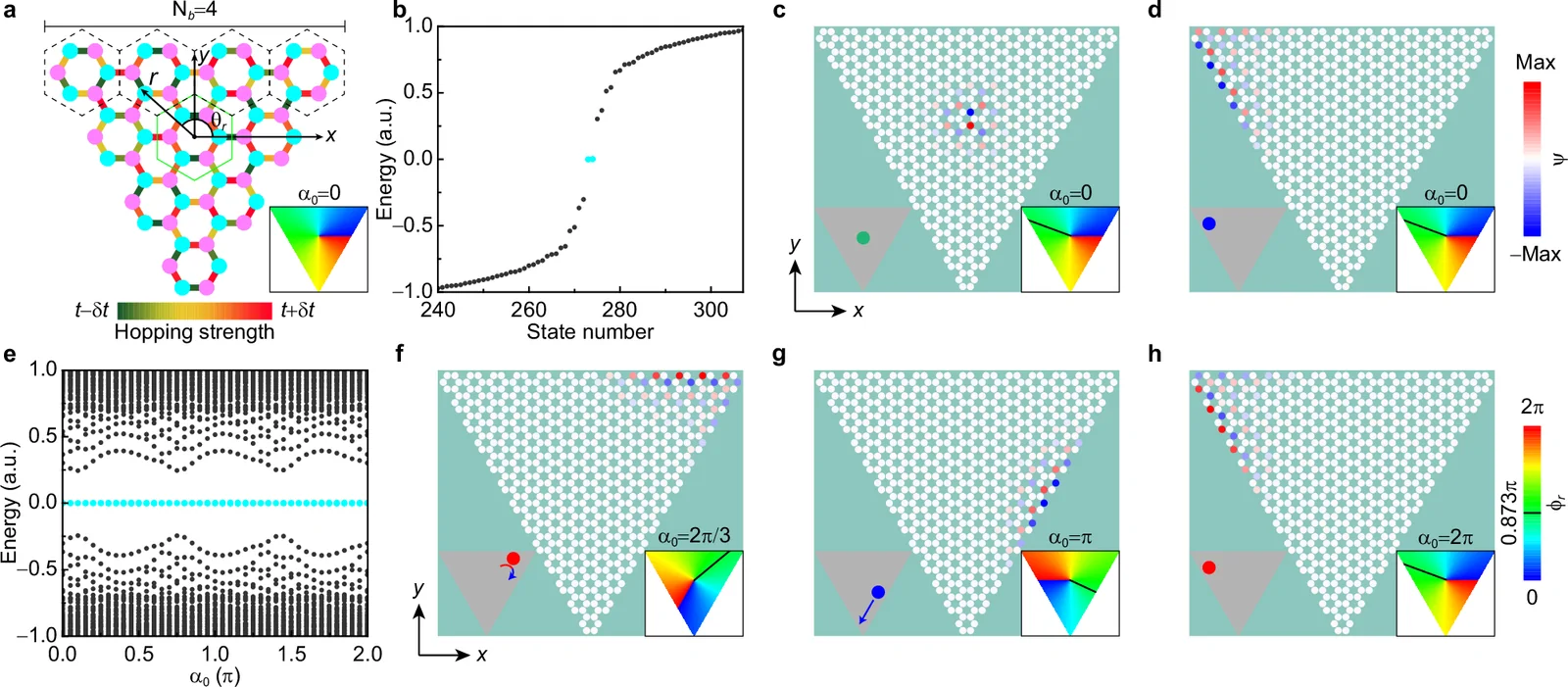
Tight-binding model and MLZMs. **a** Tight-binding model of a graphene lattice under Kekulé vortex modulation. The lattice points in cyan (magenta) correspond to sublattice ${\Lambda }_{a}$ (${\Lambda }_{b}$). The hopping strengths, modulated by the Kekulé vortex texture, are represented by the color of the bonds. The vortex center is set at the center of the triangular shape, with an armchair boundary containing ${N}_{b}$ lattice sites. **b** Eigen energy spectrum of the triangular-shaped system in the vicinity of zero energy for an initial offset ${\alpha }_{0}=0$. Cyan dots denote the two MLZMs. Eigenfield distributions of the CMLZM (**c**) and BMLZM (**d**) for ${\alpha }_{0}=0$. **e** Eigen energy spectrum of the triangular-shaped system for different values of ${\alpha }_{0}$. The cyan dots (doubly degenerate) and the black dots represent the MLZMs and bulk states, respectively. Eigenfield distributions of the BMLZM with ${\alpha }_{0}=2\pi /3$ (**f**), π (**g**), and 2π (**h**). The lower-right insets show the distributions of the modulation phase ${\phi }_{{{{\boldsymbol{r}}}}}$, and the black lines in (**c**, **d**, **f**-**h**) mark the position with ${\phi }_{{{{\boldsymbol{r}}}}}=0.873\pi$. The lower-left insets depict the locations of the MLZMs, and the color arrows indicate the evolution directions with the increase of ${\alpha }_{0}$. In (**b**–**h),** ${N}_{b}=13$, ${t}_{0}=1$, and ${\Delta }_{0}=-1.5$.

We start with a tight-binding model (Fig. 1a) with a graphene lattice modulated by a vortex-textured Kekulé distortion, whose Hamiltonian reads^39^

1 $$H={\sum }_{{{{\boldsymbol{r}}}}\in {\Lambda }_{a}}{\sum }_{i=1}^{3}\left({t}_{0}+{\delta t}_{{{{\boldsymbol{r}}}},i}\right){a}_{{{{\boldsymbol{r}}}}}^{{{\dagger}} }{b}_{{{{\boldsymbol{r}}}}+{{{{\boldsymbol{s}}}}}_{{{{\boldsymbol{i}}}}}}+{{{\rm{H}}}}.{{{\rm{c}}}}.,$$

where ${a}_{{{{\boldsymbol{r}}}}}$ (${b}_{{{{\boldsymbol{r}}}}{{{\boldsymbol{+}}}}{{{{\boldsymbol{s}}}}}_{{{{\boldsymbol{i}}}}}}$) is the annihilation operator at site ${{{\boldsymbol{r}}}}$ (${{{\boldsymbol{r}}}}{{{\boldsymbol{+}}}}{{{{\boldsymbol{s}}}}}_{{{{\boldsymbol{i}}}}}$) belonging to sublattice ${\Lambda }_{a}$ (${\Lambda }_{b}$). The vector ${{{{\boldsymbol{s}}}}}_{{{{\boldsymbol{i}}}}}$ with $i=1,\,2,\,3$ connects points at site ${{{\boldsymbol{r}}}}$ to the three nearest-neighbor lattice sites. The original graphene hopping strength ${t}_{0}$ is modulated by an additional term $\delta {t}_{{{{\boldsymbol{r}}}},i}={\Delta }_{0}{e}^{i({{{{\boldsymbol{K}}}}}_{+}\cdot {{{{\boldsymbol{s}}}}}_{i}+({{{{\boldsymbol{K}}}}}_{{{{\boldsymbol{+}}}}}-{{{{\boldsymbol{K}}}}}_{-}){{{\boldsymbol{\cdot }}}}{{{\boldsymbol{r}}}}+{\phi }_{{{{\boldsymbol{r}}}}})}/3+{{{\rm{c}}}}.{{{\rm{c}}}}.$, where ${K}_{\pm }(0,\pm \frac{4\pi }{3a})$ labels the original Dirac points of the unmodulated graphene lattice, and the modulation phase ${\phi }_{{{{\boldsymbol{r}}}}}={\theta }_{{{{\boldsymbol{r}}}}}+{\alpha }_{0}$ contains the contribution of the azimuth angle ${\theta }_{{{{\boldsymbol{r}}}}}$ of site ${{{\boldsymbol{r}}}}$ relative to the vortex center and an initial offset ${\alpha }_{0}$. The lattice is terminated at three armchair boundaries whose outer normal directions are all parallel to $\Gamma {K}_{+}$, as depicted in Fig. 1a. Unlike the zigzag boundary and other irregular boundaries, the armchair boundary considered herein is free of trivial zero-energy modes (see Supplementary Note I). The vortex center is chosen at the center of the triangular shape, and the lower-right inset of Fig. 1a shows the distribution of ${\phi }_{{{{\boldsymbol{r}}}}}$ for ${\alpha }_{0}=0$. The number of lattices used in the calculation is not shown entirely in Fig. 1a. We use ${N}_{b}$ to denote the number of lattices along each armchair boundary, e.g., ${N}_{b}=4$ in Fig. 1a.

The eigenvalue spectrum of a vortex texture Kekulé modulated graphene with ${\alpha }_{0}=0$ and ${N}_{b}=13$ is shown in Fig. 1b. The black dots denote the bulk modes. Two zero-energy modes (highlighted in cyan) emerge within the bandgap. One is bounded at the center of the Kekulé vortex, whose eigenfield is only located on sublattice ${\Lambda }_{b}$ (Fig. 1c), while the other one is bounded at the boundary and is located only on sublattice ${\Lambda }_{a}$ (Fig. 1d). The vortex-center–bound MLZM (CMLZM) has been investigated in previous studies^25,28,44,45^; however, the BMLZM remains largely unexplored. In Supplementary Note II, we show that although the eigenfield profiles differ, the low-energy effective Hamiltonians of the BMLZM and CMLZM are identical. Moreover, a BMLZM can be annihilated by a CMLZM carrying an opposite topological charge, indicating that both modes share the same topological origin.

The distinct parameter dependence of the BMLZM compared with the CMLZM introduces an additional degree of freedom for controlling the BMLZM’s location. In particular, the position of the CMLZM is independent of ${\alpha }_{0}$, while that of the BMLZM varies continuously with ${\alpha }_{0}$. To illustrate this behavior, we adiabatically increase ${\alpha }_{0}$ from 0 to 2π, and the eigenenergy spectrum is shown in Fig. 1e. Similar to Fig. 1b, the black dots represent bulk states and the cyan dots denote the CMLZM and BMLZM, which remain doubly degenerate. Figure 1f–h display three representative eigenfield distributions of the BMLZMs, with the lower-right insets showing the associated modulation phase distribution ${\phi }_{{{{\boldsymbol{r}}}}}$ (see Supplementary Note III for other ${\phi }_{{{{\boldsymbol{r}}}}}$). As ${\alpha }_{0}$ increased to 2π, the BMLZM rotates clockwise along the boundary and returns to its initial position at last (see also the location denoted by the dots in the lower-left inset in Fig. 1d,f–h). Intriguingly, irrespective of the initial offset ${\alpha }_{0}$, the position of the BMLZM consistently centers at ${\phi }_{{{{\boldsymbol{r}}}}}=0.873\pi$, as labeled by black lines in the lower-right insets of Fig. 1c, d, f–h. This specific modulation phase corresponds to the phase-transition point of the boundary bands, analogous to the well-known Jackiw-Rebbi mechanism^48^ (see Supplementary Note II).

Although the system returns to the same Hamiltonian, a nontrivial phase arises during the braiding process, where the BMLZM encircles the CMLZM once (see Supplementary Note IV). As evident from Fig. 1d and 1h, the BMLZM acquires an additional π phase—manifested by a sign reversal of the outermost field amplitudes—as ${\alpha }_{0}$ increases to 2π. For the system configuration shown in Fig. 1a, this π phase is acquired when the BMLZM crosses the lattice corner (Fig. 1f). Specifically, the field components at the sites nearest to the corner exhibit a sign reversal between the two sides of the corner, while those for the sites along the boundary keep the same (see details in Supplementary Note V). Thus, at the end of an evolution with ${\alpha }_{0}=2\pi$, the BMLZM achieves an additional 3π phase. Taking into account the 2π periodicity of the phase, the BMLZM undergoes a net π phase shift, akin to the braiding process of two CMLZMs^44,45^.

### BMLZMs for acoustic waves

**Fig. 2:**
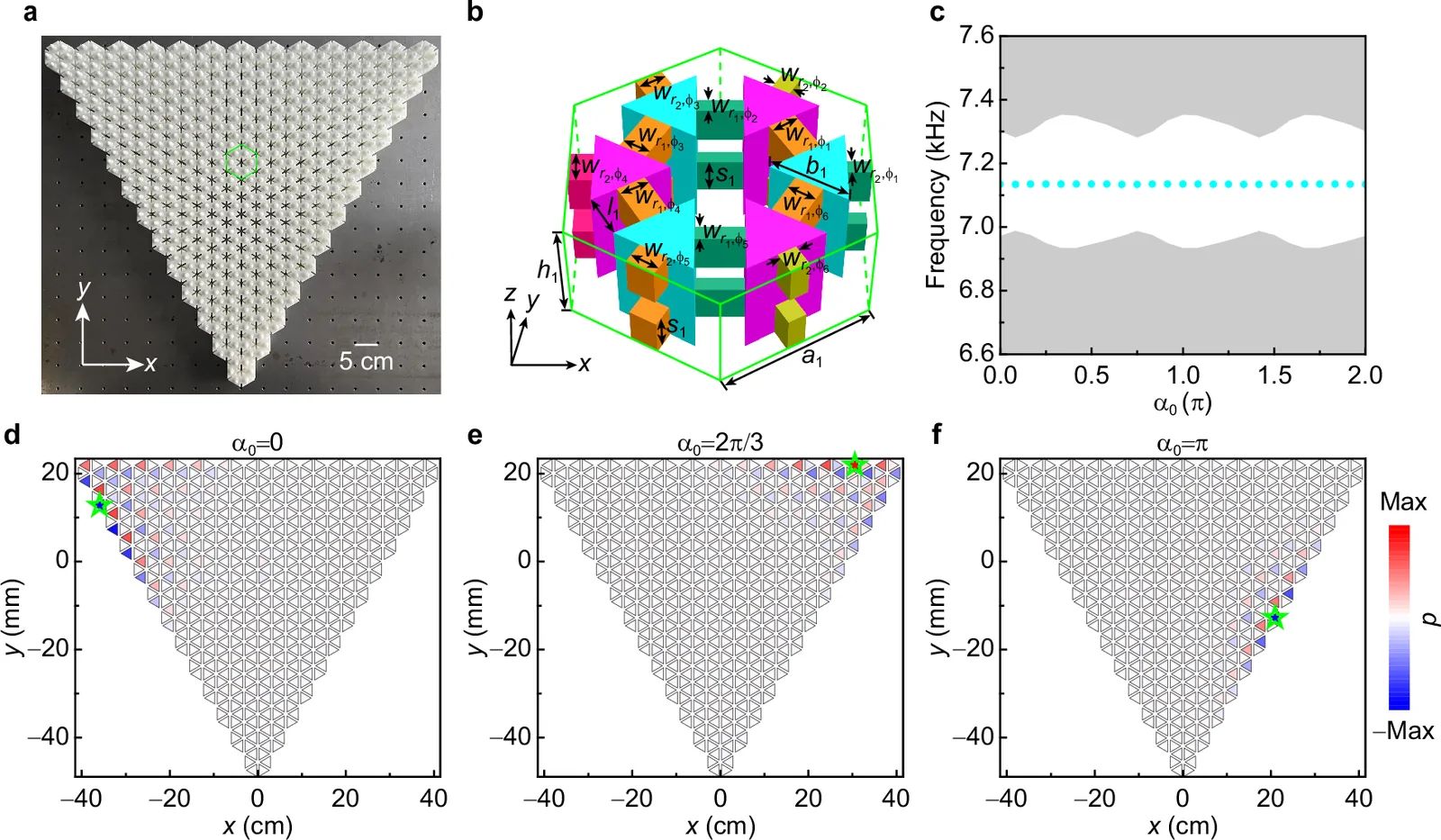
BMLZM measured in PCs with different initial offset *α*_0_. **a** Photograph of a single layer triangular-shaped PC sample with ${N}_{b}=13$. **b** Schematic of a unit cell (highlighted by the green hexagon in (**a**) with key geometric parameters marked. The prism-shaped resonant cavity in cyan (magenta) emulates the sublattice ${\Lambda }_{a}$ (${\Lambda }_{b}$), while the horizontal tubes mimic the couplings. **c**, Eigenfrequency of the BMLZM (cyan dots) for different values of ${\alpha }_{0}$, where the gray regions denote the bulk states. Measured acoustic field distributions of the BMLZMs at ${\alpha }_{0}=0$ (**d**), 2π/3 (**e**), and π (**f**). The green stars indicate the positions of the sound sources operating at $7.14\,{{{\rm{kHz}}}}$.

We next implement the Hamiltonian in Eq. (1) in a phononic crystal (PC) platform, thereby revealing the braiding behavior of MLZMs manifested in acoustic waves. Figure 2a shows a triangular-shaped PC sample with ${N}_{b}=13$ fabricated by 3D printing technology. As shown in Fig. 2b, the unit cell consists of triangular-prism acoustic resonators supporting dipolar modes along the *z* direction, arranged in a graphene lattice with a side length of ${a}_{1}=3.65\,{{{\rm{cm}}}}$. The triangular prisms point to the left (cyan) and right (magenta) sides for the resonators located on the ${\Lambda }_{a}$ and ${\Lambda }_{b}$ sublattices, respectively. The height and side lengths of resonators are ${h}_{1}=2.52\,{{{\rm{cm}}}}$ and ${b}_{1}=2.18\,{{{\rm{cm}}}}$, respectively. The resonators are connected by pairs of parallel cuboid tubes (length ${l}_{1}=0.85\,{{{\rm{cm}}}}$) that emulate the coupling between them. The tube height is fixed at ${s}_{1}=0.90\,{{{\rm{cm}}}}$, while their widths ${w}_{{{{\boldsymbol{r}}}},i}$ —which are linearly proportional to the coupling strength—are modulated as ${w}_{{{{\boldsymbol{r}}}},i}=(0.81+\delta {w}_{{{{\boldsymbol{r}}}},i})\,{{{\rm{cm}}}}$, where $\delta {w}_{{{{\boldsymbol{r}}}},i}={\Delta }_{w}{e}^{i\left({{{{\boldsymbol{K}}}}}_{+}\cdot {{{{\boldsymbol{s}}}}}_{i}+{2{{{\boldsymbol{K}}}}}_{+}\cdot {{{\boldsymbol{r}}}}+{\phi }_{{{{\boldsymbol{r}}}}}\right)}/3+{{{\rm{c}}}}.{{{\rm{c}}}}.$ with ${\Delta }_{w}=0.32$.

The eigenspectrum of the BMLZM for different values of ${\alpha }_{0}$ is shown in Fig. 2c, where the cyan dots denote the BMLZMs and the gray regions represent the bulk states. The eigenfrequencies of the BMLZMs remain nearly independent of ${\alpha }_{0}$ and are located within the bulk bandgap. Further details of the simulated eigenspectra and eigenfield distributions are provided in Supplementary Note VI. To experimentally observe the BMLZMs, we fabricate three representative samples with ${\alpha }_{0}=0$, 2π/3, and π. A dipolar sound source is placed at the sample boundary corresponding to the modulation phase ${\phi }_{{{{\boldsymbol{r}}}}}=0.873\pi$ (indicated by green stars in Fig. 2d–f). Figure 2d–f display the measured acoustic field distributions inside each resonator at $7.14\,{{{\rm{kHz}}}}$ for ${\alpha }_{0}=0$, 2π/3, and π, respectively. The acoustic fields are predominantly localized on the left-oriented resonators (${\Lambda }_{a}$ sublattices), in close agreement with the BMLZMs predicted by the tight-binding model in Fig. 1d, f, g. Furthermore, the sign of the field amplitude for the outermost resonators remains consistent along the boundary (Fig. 2d, f), but reverses across the sample corner (Fig. 2e).

### Braiding of the BMLZMs in acoustic systems

**Fig. 3:**
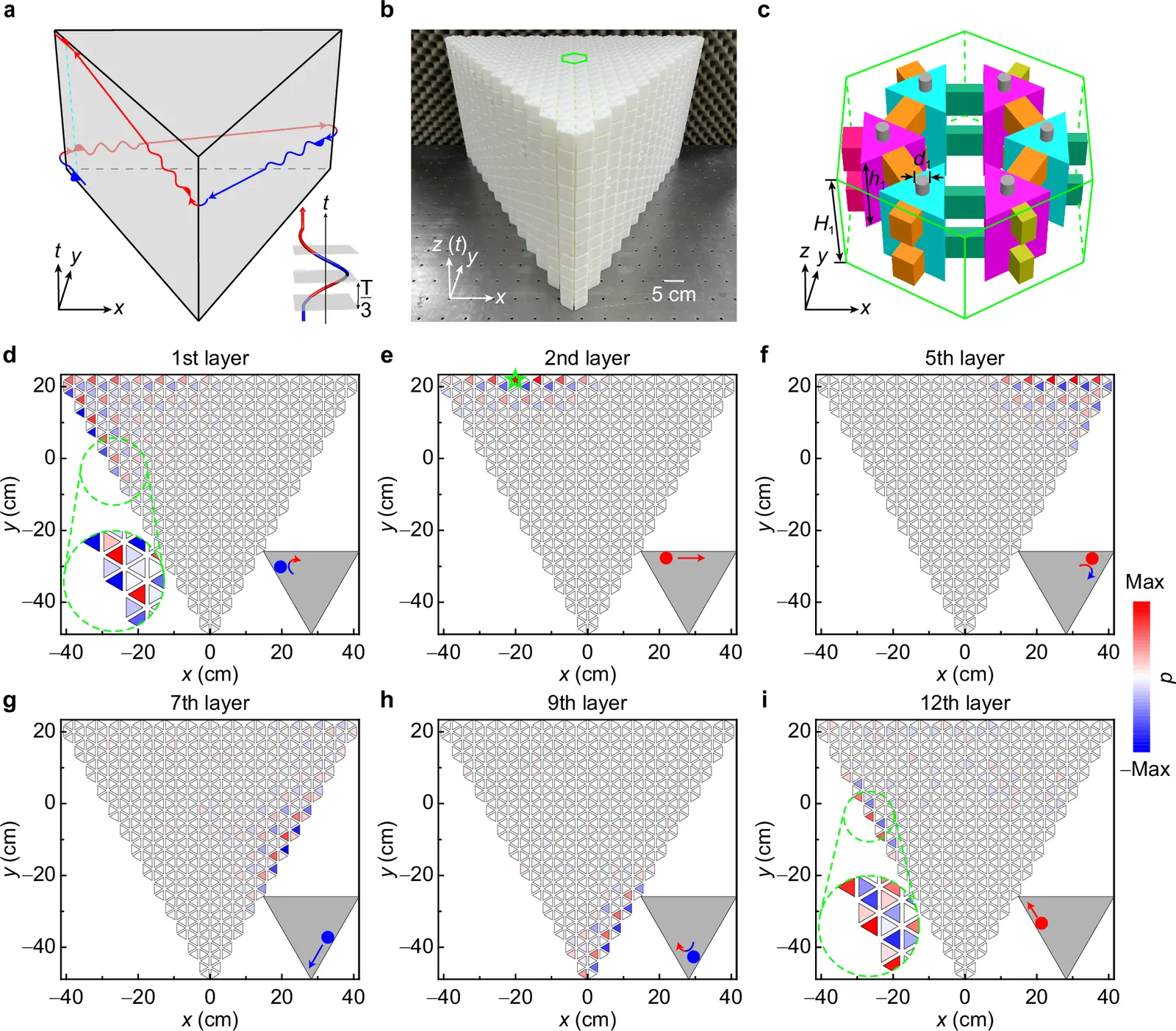
Evolution of the BMLZM in a 3D PC. **a** Evolution trajectory of the BMLZM in the triangular-prism space [*x*–*y*–*z*(*t*)]. The inset highlights the evolution process, with *T* representing the braiding period under ${\phi }_{r}\to {\phi }_{r}+2\pi$. **b** Photograph of the triangular-prism PC sample, constructed by stacking 13 layers of the PCs with different ${\alpha }_{0}$. The ${\alpha }_{0}$ of the first layer is set to 0, and that of each subsequent layer increases by π/6 relative to the layer below. **c**, Schematic of a unit cell, where six gray circular tubes are introduced to provide weak interlayer coupl**i**ng along the z direction. **d**–**i** Measured field distributions of the BMLZMs on six representative layers (layers 1, 2, 5, 7, 9, and 12), corresponding to ${\alpha }_{0}=0$, π/6, 2π/3, π, 4π/3, and 11π/6, respectively. The lower-right insets show the positions of the BMLZMs, with the colored arrows indicating their evolution directions as the layer number increases. The green star in (**e**) marks the position of the sound source operating at $7.23\,{{{\rm{kHz}}}}$. The geometry of the PC in each layer follows the same parameters as in Fig. 2, with ${H}_{1}=3.32\,{{{\rm{cm}}}}$ and ${d}_{1}=0.35\,{{{\rm{cm}}}}$.

The field evolution of the MLZMs during the braiding process can be revealed by slowly varying ${\alpha }_{0}$. As illustrated in Fig. 3a, a schematic shows a BMLZM encircling the side boundary of a triangular-prism sample, where the propagation of the BMLZM in the z direction can be regarded as an evolution in a synthetic time t. The actual 3D PC sample is shown in Fig. 3b, consisting of 13 layers formed by stacking the PC layers from Fig. 2 while gradually increasing ${\alpha }_{0}$. The ${\alpha }_{0}$ of the first layer is set to 0, and that of each subsequent layer increases by π/6 relative to the layer below. As a result, the ${\alpha }_{0}$ of the 13th layer reaches 2π, restoring the same geometric configuration as the first layer. Figure 3c illustrates a schematic of a unit cell, with a lattice constant along the *z* direction of ${H}_{1}=3.32\,{{{\rm{cm}}}}$. The geometry of the layered structure remains the same as before, but six gray cylindrical tubes are introduced to provide weak interlayer coupling. Consequently, when excited, the BMLZM propagates slowly along the z direction as ${\alpha }_{0}$ varies. After reaching the 13th layer, the horizontal position of the BMLZM returns to that of the first layer. This design not only enables the realization of MLZM braiding in a physical PC but also allows direct observation of the BMLZMs during this process through steady-state acoustic field measurements.

Figure 3d–i display the measured field distributions for six representative layers; results for the remaining layers are provided in Supplementary Note VII. The system is excited by a point dipolar source located at the boundary of the second layer (green star in Fig. 3e; see Methods for experimental details). Owing to the weak interlayer coupling introduced by the small z-direction tubes, the resonance frequencies of the resonators—and consequently that of the BMLZM—are slightly shifted from $7.14\,{{{\rm{kHz}}}}$ to $7.23\,{{{\rm{kHz}}}}$. In addition, as airborne sound attenuates during interlayer propagation, the acoustic pressures are normalized to the maximum value within each layer for better illustration. In Fig. 3d–i, the arrows in the lower-right insets indicate the lateral shift direction of the BMLZM as it propagates along the +z direction, while their colors denote the phase of the outermost resonators (red and blue corresponding to positive and negative amplitudes, respectively). The measurements clearly reveal that the BMLZM propagates clockwise along the sample boundary as the layer index increases. Figure 3d and 3f further demonstrate the π phase acquired when crossing a lattice corner, and after a complete evolution, the BMLZM accumulates a net π phase shift (see enlarged views in the lower-left insets of Fig. 3d and 3i).

**Fig. 4:**
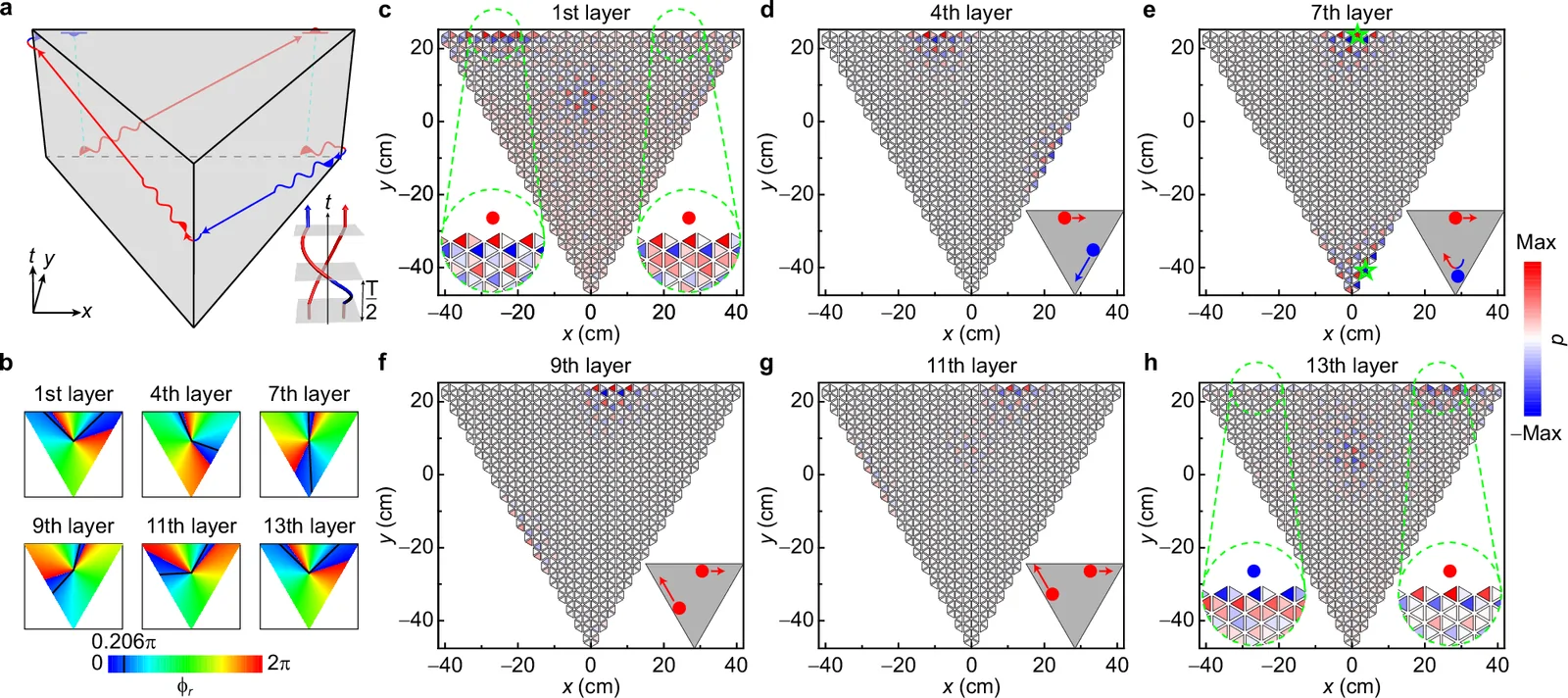
Braiding of two BMLZMs in a 3D PC. **a** Braiding trajectory of the two BMLZMs in a triangular-prism space (x-y-t). The inset illustrates the schematic of the braiding process, with T representing the period for the BMLZMs to rotate back to the initial configuration when the modulation phase ${\phi }_{r}\to {\phi }_{r}+2\pi$. **b** The distributions of the modulation phase ${\phi }_{{{{\boldsymbol{r}}}}}$ for six representative layers (layers 1, 4, 7, 9, 11, and 13), corresponding to ${\alpha }_{0}=0$, π/2, π, 4π/3, 5π/3, and 2π. The black lines mark the position with ${\phi }_{{{{\boldsymbol{r}}}}}=0.206\pi$. **c**–**f** Measured field distributions of the BMLZMs on six representative layers, respectively. The lower-right insets mark the positions of the BMLZMs, with the colored arrows indicating their evolution directions as the layer number increases. The insets in (**c** and **h**) present enlarged views of the normalized field distributions of the two BMLZMs, showing that th**e**y are in phase in **c** and out of phase in (**h**). The green stars in **e** mark the positions of the sound sources operating at $9.96\,{{{\rm{kHz}}}}$. ${N}_{b}=18$ and other geometric parameters of the PC are provided in Supplementary Note IX. Due to unavoidable experimental loss, the signal of the BMLZM on the right-hand side of the first layer, or equivalently on the left-hand side of the 13th layer, propagates over a longer distance and is therefore much weaker than that of the BMLZM on the opposite side.

The braiding rule becomes richer when more MLZMs are involved. In Supplementary Note VIII, we discuss in detail the archetypes of the braiding rules when including more MLZMs. In the experiment, we demonstrate the braiding of two BMLZMs around another two CMLZMs in a 3D PC. The experimental setup is similar to that in Fig. 3, where the propagation along the *z* direction is treated as the synthetic time-evolution axis. The schematic of the braiding of two BMLZMs around another two CMLZMs is illustrated in Fig. 4a. The sample consists of 13 layers, with ${\alpha }_{0}$ gradually increasing from 0 to 2π in steps of π/6. To minimize the interaction between the two BMLZMs, the sample size is increased to ${N}_{b}=18$. Figure 4b shows the distributions of the modulation phase ${\phi }_{{{{\boldsymbol{r}}}}}$ for six representative layers (layers 1, 4, 7, 9, 11, and 13), corresponding to ${\alpha }_{0}=0$, π/2, π, 4π/3, 5π/3, and 2π. The black lines mark the position with ${\phi }_{r}=0.206\pi$ where the BMLZMs are located at (see Supplementary Note II). Initially, on the first layer, both BMLZMs are located at the upper boundary of the triangular sample. Considering the unavoidable propagation loss, the size of all resonators is simultaneously reduced, resulting in a BMLZM resonance frequency of $9.96\,{{{\rm{kHz}}}}$. Details of the experimental setup are provided in Supplementary Note IX.

Figure 4c–h show the measured field distributions for six representative layers, with results for the remaining layers provided in Supplementary Note IX. A pair of point dipolar sources are used to excite the two BMLZMs (see experimental details in Methods). As shown in the enlarged field maps in Fig. 4c, the two BMLZMs share the same phase, denoted by $|{\psi }_{0} > ={\left[{{\mathrm{1,1}}}\right]}^{{{{\rm{T}}}}}$ on the first layer. As the layer number increases, the two BMLZMs rotate clockwise, with the colored arrows in the lower-right insets indicating their braiding directions. Upon adiabatically increasing the modulation phase ${\phi }_{r}\to {\phi }_{r}+2\pi$, the positions of the two BMLZMs are exchanged when reaching the 13th layer. However, while the phase of the right BMLZM on the 13th layer remains the same as that on the first layer, the left BMLZM acquires an additional π phase. In other words, the final state of the two BMLZMs after braiding is $|{\psi }_{T} > ={\left[-{{\mathrm{1,1}}}\right]}^{{{{\rm{T}}}}}$, consistent with the braiding rules discussed in Supplementary Note VIII. Due to unavoidable experimental imperfections, the CMLZMs at the center of the triangular sample are also weakly excited (see Fig. 4c, 4h). In practice, as the two CMLZMs cannot be spatially well-separated within the limited sample size, the implementation of braiding operations involving only CMLZMs becomes significantly more demanding.

## Discussion

In conclusion, we have theoretically demonstrated and experimentally realized the braiding of MLZMs in PCs composed of a vortex-textured Kekulé-modulated graphene lattice. Unlike previous studies focusing on CMLZMs^25,28,44,45^, our approach manipulates the positions of BMLZMs, which can be tuned far apart within a finite-sized sample. This enables a real-space, field-resolved experimental observation of the braiding process. The successful realization of adiabatic braiding involving multiple BMLZMs paves the way for both quantum and classical applications based on similar schemes. Our system can be implemented in other platforms such as photonic crystals^49^, cold atoms^50^, and circuits^51^. Consequently, our findings provide new possibilities for manipulating topological zero modes, offering promising candidates for implementing non-Abelian braiding.

## Methods

### Simulations

All numerical simulations of the PC were performed using COMSOL Multiphysics, a commercial finite-element solver. The PCs were filled with air, modeled with a mass density of $1.29\,{{{\rm{kg}}}}/{{{{\rm{m}}}}}^{3}$ and a sound velocity of $344{{{\rm{m}}}}/{{{\rm{s}}}}$. The 3D-printed photosensitive resin was treated as acoustically rigid owing to the large acoustic-impedance mismatch between the resin and air.

### Experiments

The experimental PC samples were fabricated using 3D printing technology with photosensitive resin. The samples feature a cavity-shell configuration, with the shell thickness measuring $2.5\,{{{\rm{mm}}}}$. In the experiments, a multi-analyzer system (B&K Type 3560B) was used to generate and analyze signals. Two loudspeakers (diameter ~$4.0{{{\rm{mm}}}}$) driven out of phase to form a dipolar source were used to generate sound and excite the dipolar mode within each cavity. The positions of the dipolar sources are indicated by the green stars in Figs. 2d–2f, 3e and 4e. Two acoustic detectors were utilized for sound recording: one acoustic detector (B&K Type 4138 1/8-inch microphone) was positioned near the sound source to collect the reference signal, and the other detector (B&K Type 4182 micro-tube probe, diameter ~$1.3{{{\rm{mm}}}}$) was scanned point by point to map the acoustic field distributions. In Fig. 2, where the system consists of a single layer, the scanning field detector was inserted into the resonant cavity through a probe hole, which is otherwise sealed with a tight-fitting stopper when not in use. In Figs. 3 and 4, where the system comprises 13 layers, the bulk fields were measured using a scanning detector equipped with a sufficiently long probe tip, inserted into the sample from the top. In all measurements, the acoustic signals were recorded at nearly identical positions within the upper half of each resonator.

## Supplementary information


Supplementary Information
Transparent Peer Review file


## Data Availability

All the data supporting this study are displayed in the main text and Supplementary Information. Additional data related to this study are available from the corresponding authors upon request.

## Code availability

The codes that support the results of this study are available from the corresponding authors upon request.
