# Supplementary material for: Direct observation of braiding of Majorana-like zero modes in real space
Source: Nat Commun. 2026 Jul 31;17:9279. doi: 10.1038/s41467-026-75765-4 (PMC13527056; doi:10.1038/s41467-026-75765-4)
Supplement: Supplementary file 1 [file 41467_2026_75765_MOESM1_ESM.pdf]

Supplemental Information for
"Direct observation of braiding of Majorana-like zero modes in real space"

Qiyun Ma[1,†], Hailong He[1,†], Weiyin Deng[1], Meng Xiao,[1,2,*], and Zhengyou Liu[1,3,*]

[1]Key Laboratory of Artificial Micro- and Nano-structures of Ministry of Education and School of Physics and Technology, Wuhan University, Wuhan 430072, China

[2]Wuhan Institute of Quantum Technology, Wuhan 430206, China

[3]Institute for Advanced Studies, Wuhan University, Wuhan 430072, China

[†]These authors contributed equally: Qiyun Ma and Hailong He.

*e-mail: phmxiao@whu.edu.cn, zyliu@whu.edu.cn.

The Supplementary Information includes:

**S-I.**	**Zero modes for different boundaries (Figs. S1-S4)**
**S-II.**	**Basic properties of BMLZMs (Figs. S5-S8 and Table S1)**
**S-III.**	**The position of BMLZM for different α_0 (Figs. S9,S10)**
**S-IV.**	**BMLZM braided with the CMLZM (Fig. S11)**
**S-V.**	**Continuity boundary condition of BMLZMs at lattice corners (Fig. S12)**
**S-VI.**	**CMLZM and BMLZM in discrete phononic crystals (PCs) (Fig. S13,S14)**
**S-VII.**	**Response spectra and field distributions of PCs containing one BMLZM (Figs. S15,S16)**
**S-VIII.**	**Braiding rule of multiple MLZMs (Fig. S17)**
**S-IX.**	**Two BMLZMs in one PC (Figs. S18-S21)**
**S-X.**	**Multiple BMLZMs in one PC (Fig. S22)**

S-I. Zero modes for different boundaries

In the main text, we focus on the zero modes in the lattice with armchair boundaries for simplicity since these boundaries have no trivial zero modes. Here, we discuss the cases when the lattices exhibit different boundaries, such as zigzag boundaries.

We first discuss the rectangular-shaped uniform-Kekulé-texture modulated lattice with two zigzag and two armchair boundaries, as shown in Fig. S1a. The lattice points in cyan (magenta) belong to sublattice Λ_a (Λ_b). Parameter N_x (N_y) describe the lattice lengths along the x (y) direction. The strength of the hoppings modulated by the Kekulé uniform texture is represented by the color of the bonds. Figure S1b displays the eigen energy spectrum of the lattice of $N_x = 10$ and $N_y = 11$. Eight trivial zero modes (cyan points) appear in the band gap. Figures S1c and S1d present the field amplitude distributions of two trivial zero modes. All the eight trivial zero modes are located at zigzag boundaries and the number of them depends on the length of zigzag boundaries, i.e. the value of N_y.

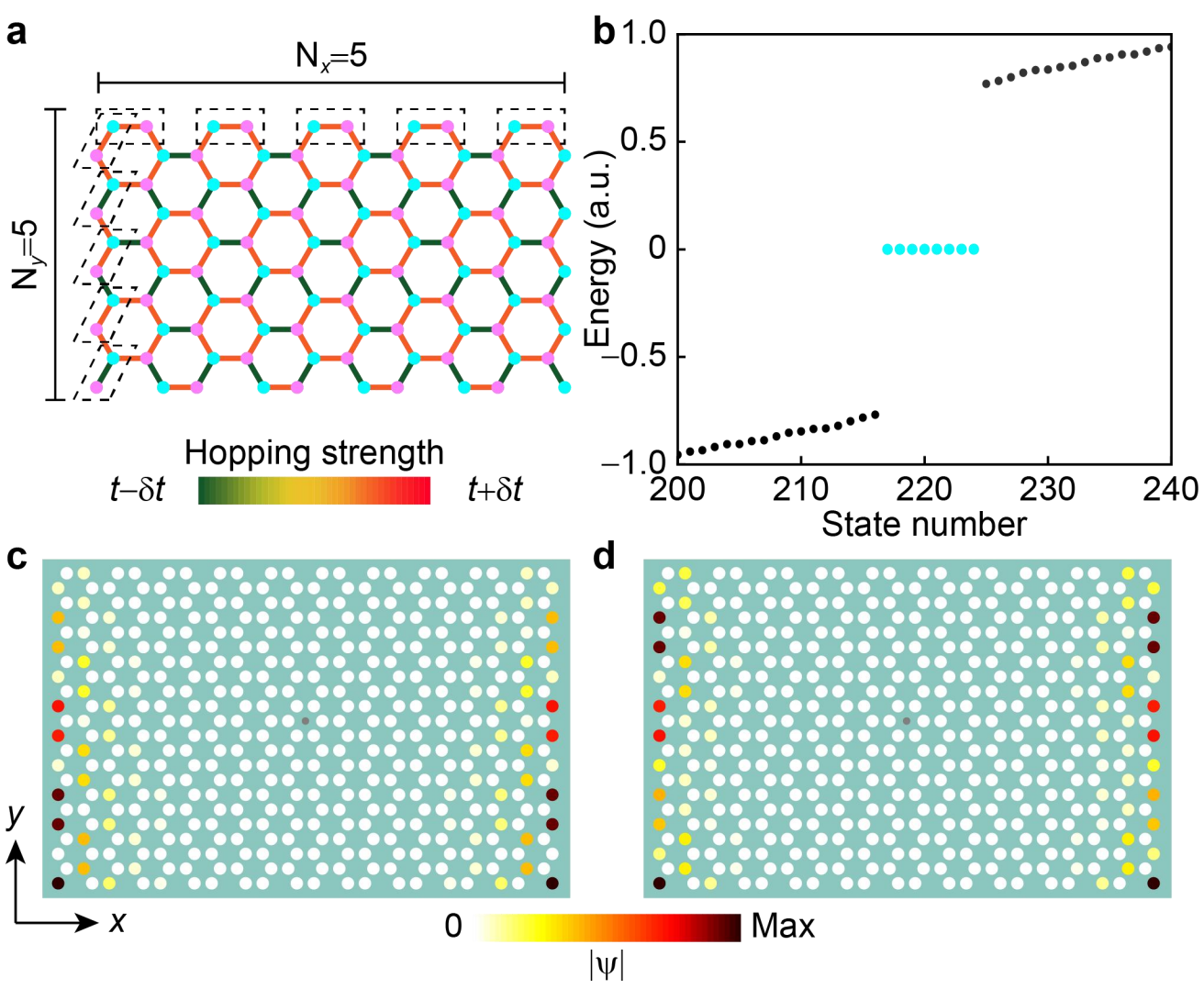

FIG. S1. | Zero modes in a rectangular-shaped honeycomb lattice with a uniform-texture Kekulé modulation. a, Tight-binding model. **b**, Eigen energy spectrum in the vicinity of zero energy. Cyan points highlight eight trivial zero modes that are only located at zigzag boundaries. **c**,**d**, Field amplitude distributions of two trivial zero modes. The grey little dots at the center of models represent the position of origin. In (**b-d**), in calculation $N_x = 10$ and $N_y = 11$.

We then study the same rectangular-shaped lattice but modulated by a vortex-Kekulé-texture, as shown in Fig. S2a. The strength of the hoppings modulated by the Kekulé vortex texture is represented by the color of the bonds. Ten zero modes (cyan points) emerge in the band gap as shown in Fig. S2b. Comparing to the uniform-texture modulation in Fig. S1, two more additional zero mode appears, one at the vortex center (CMLZM) while another appears at the lattice boundary (BMLZM). Similar as Fig. 1, the location of the BMLZM is also determined by the initial offset α_0. As shown in Fig. S2c, these two MLZMs couple with each other due to the finite size effect, resulting in their energy slightly deviates from zero energy (see Fig. S2b). Figure S2d displays the field amplitude distribution of a trivial zero mode.

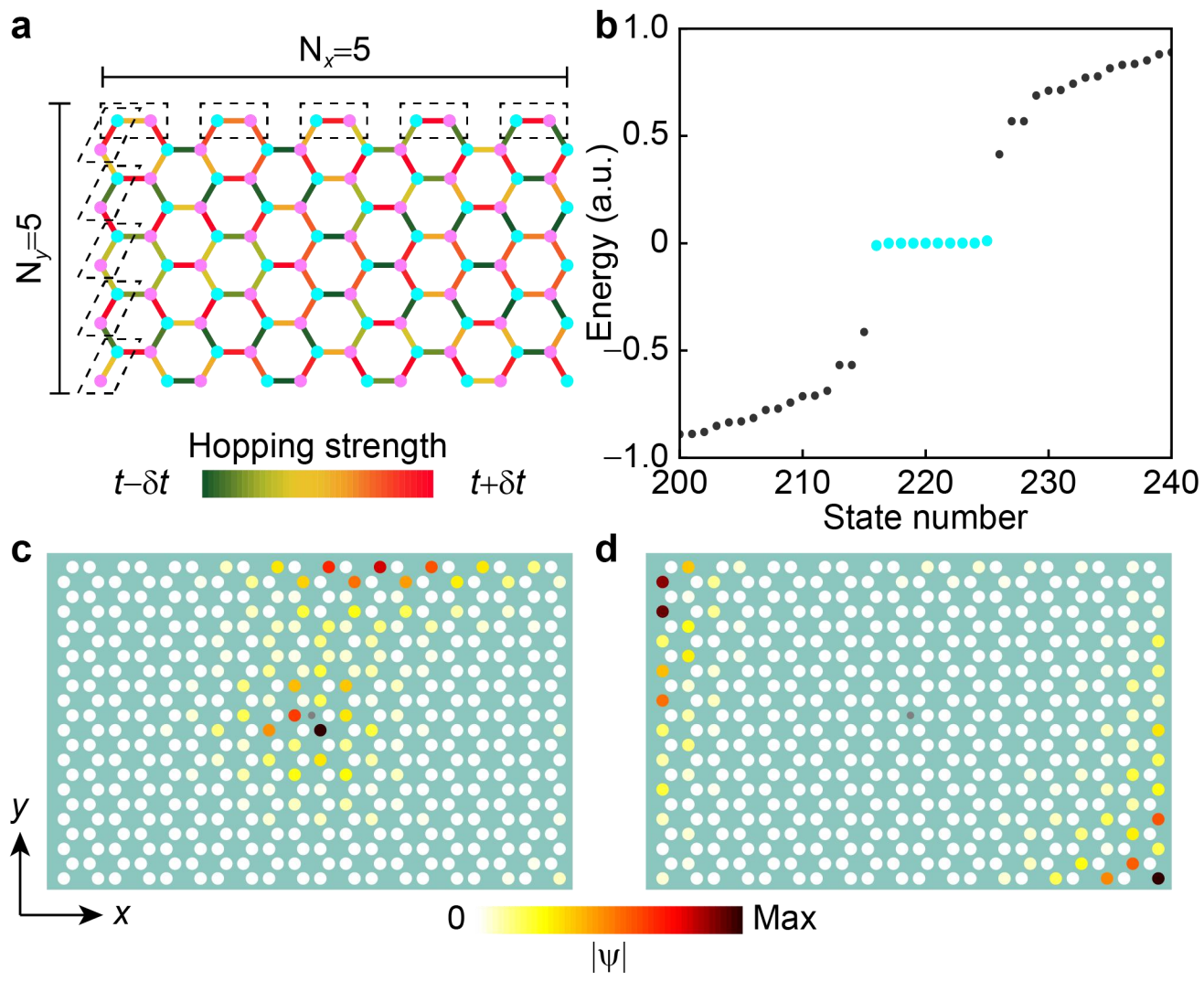

FIG. S2. | Zero modes in a rectangular-shaped honeycomb lattice with a vortex-texture Kekulé modulation. a, Tight-binding model. **b**, Eigen energy spectrum in the vicinity of zero energy. Cyan points highlight ten zero modes. **c**, Field amplitude distribution of the two coupled-MLZMs. **d**, Field amplitude distribution of a trivial zero mode. In (**c**) and (**d**), grey little dots in the center of lattices represent the location of the vortex center. In (**b-d**), in calculation $N_x = 10$, $N_y = 11$ and the initial offset of the modulation phase $\alpha_0 = 0.5\pi$.

We next discuss a more complicated case in an irregular-shaped honeycomb lattice. The lattice contains multiple kinds of boundaries, such as armchair, zigzag, and beard boundaries, as shown in Fig. S3a. The lattice points in cyan (magenta) belong to sublattice Λ_a (Λ_b). The strength of the hoppings modulated by the Kekulé uniform texture is represented by the color of the bonds. We

calculate the eigen energy spectrum of the lattice and discover that the lattice has four trivial zero boundary modes, as shown in Fig. S3b. Their energies split away a little bit from zero due to the finite size effect. Figures S3c and S3d show the distributions of two coupled field amplitude of the trivial boundary modes.

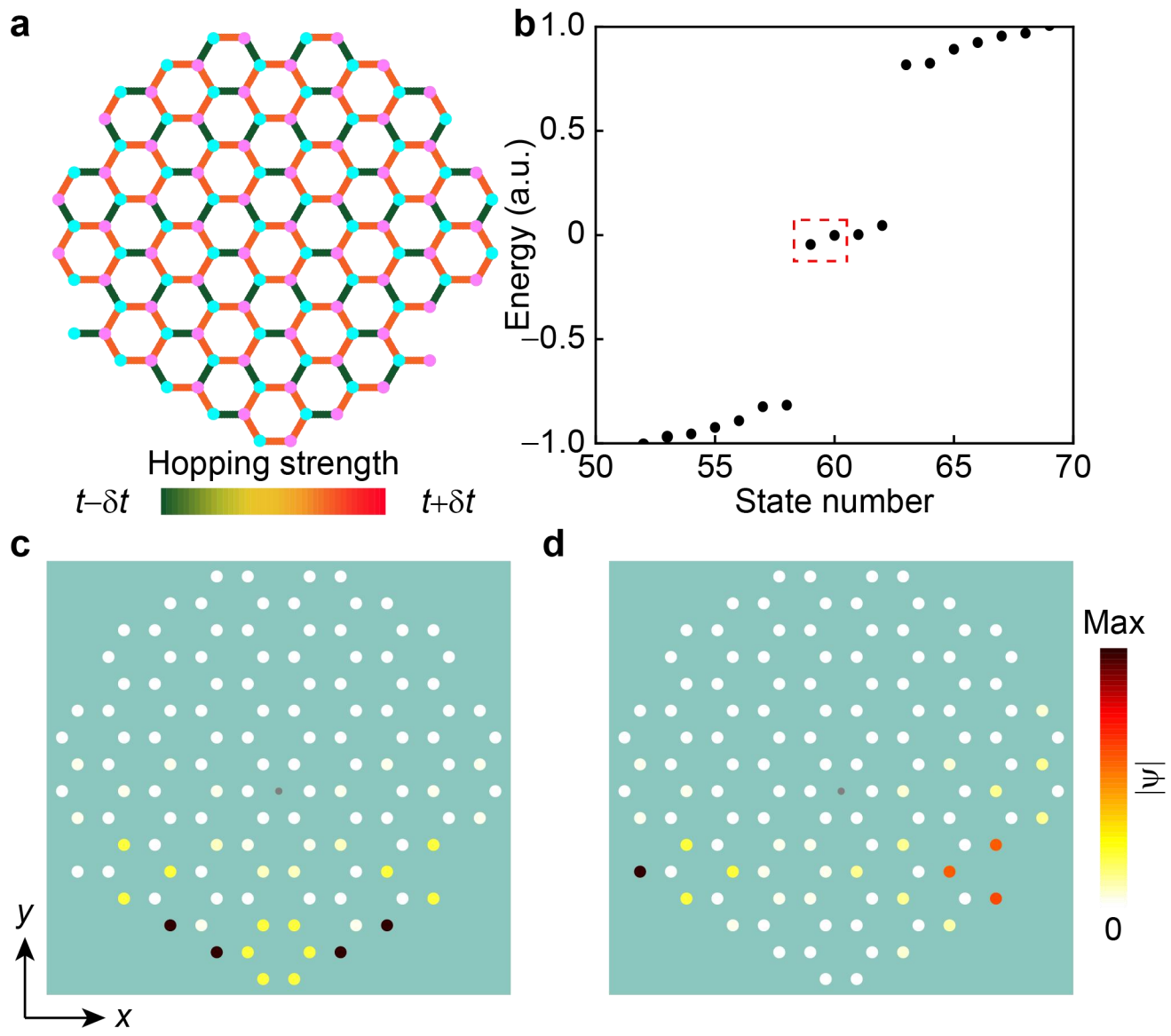

FIG. S3. | Zero modes in an irregular-shaped honeycomb lattice under a uniform-texture Kekulé modulation. a, Tight-binding model. **b**, Eigen energy spectrum in the vicinity of zero energy. The red-dotted rectangle encloses two eigenstates whose field amplitude distribution are plotted in (**c**) and (**d**). The grey little dots in (**c**) and (**d**) at the center of the lattices represent the location of origin.

We finally implement vortex-texture Kekulé modulation to the irregular-shaped honeycomb lattice discussed above, as shown in Fig. S4a. The strength of the hoppings modulated by the Kekulé vortex texture is represented by color of the bonds. Compared to the uniform-texture Kekulé case in Fig. S3, two additional modes (MLZMs) emerge near zero energy in the band gap as shown in Fig. S4b. The small deviations from zero energy of the six modes are due to the finite size effect. Figures S4c and S4d show the field amplitude distributions of two zero energy modes (whose eigenenergies are marked by the red dashed rectangular in Fig. S4b).

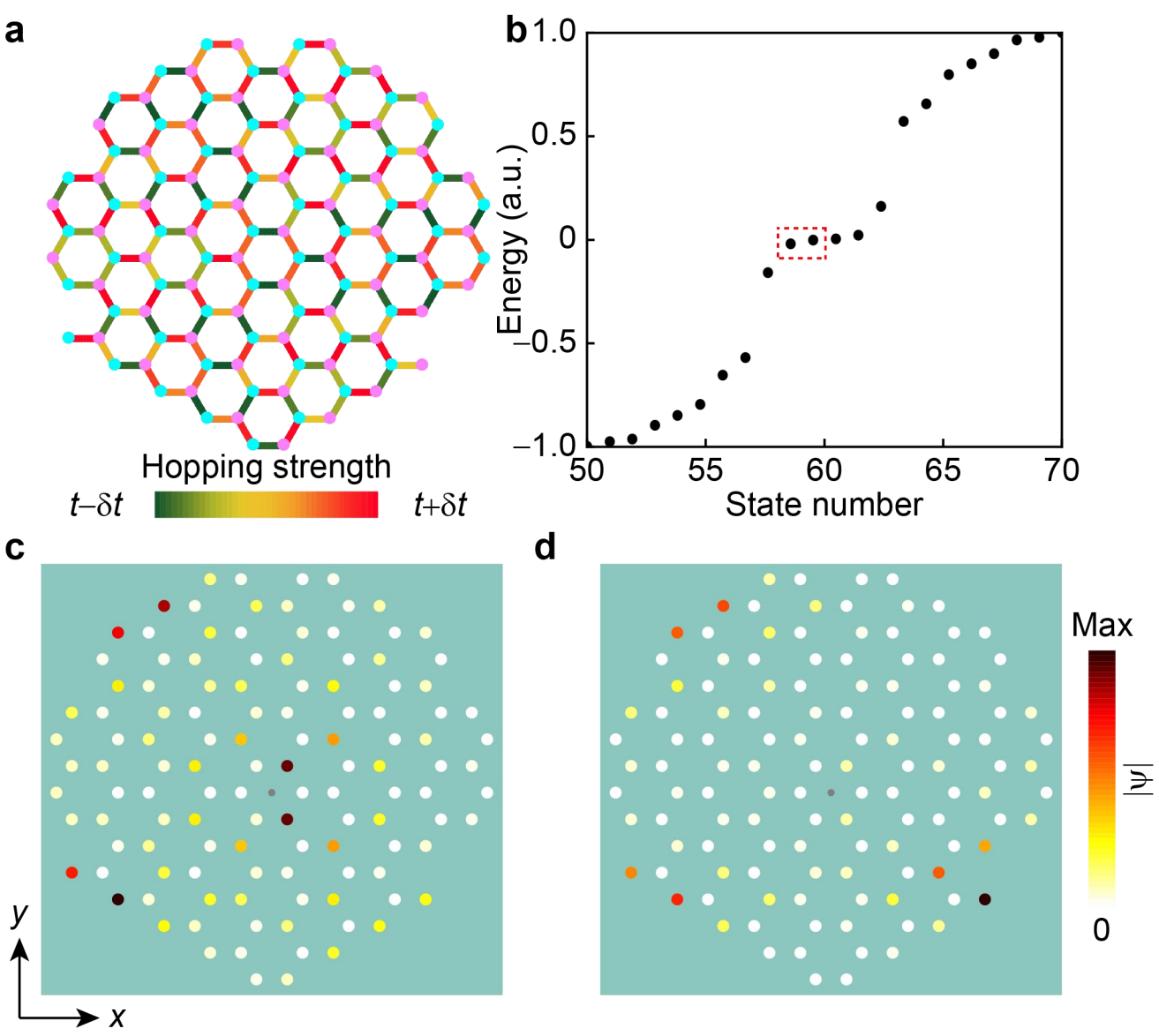

FIG. S4. | Zero modes in an irregular-shaped honeycomb lattice under a vortex-texture Kekulé modulation. a, Tight-binding model. **b**, Eigen energy spectrum in the vicinity of zero energy. The red-dotted rectangle encloses two states whose wavefunctions are plotted in (**c**) and (**d**). **c**,**d**, Distribution of coupled field amplitude of MLZMs and trivial zero modes. In (**c**) and (**d**), the grey little dots at the center of lattices represent the location of the vortex center.

S-II. Basic properties of BMLZMs

a. Low energy effective Hamiltonian and zero-energy modes

The Hamiltonian of the graphene lattice is

$$H = \sum_{r\in\Lambda_a}\sum_{i=1}^{3} t a_r^\dagger b_{r+s_i} + \mathrm{H.c.}, \tag{S1}$$

where $a_r(b_r)$ is the annihilation operators at $\boldsymbol{r}$ of the sublattice $\Lambda_a(\Lambda_b)$. Without the Kekulé modulation, a graphene lattice exhibit two Dirac points at the corners of the Brillouin zone, i.e., $K_\pm(0, \pm 4\pi/3a)$. In the vicinity of the Dirac point $\boldsymbol{k} = \mathbf{K}_\pm + \boldsymbol{\delta k}$, the $\boldsymbol{k}\cdot\boldsymbol{p}$ Hamiltonian is

$$H_{\mathbf{K}_\pm} = v_F\left[u(v)_a^\dagger \quad u(v)_b^\dagger\right]\begin{pmatrix} & \partial_x \pm i\partial_y \\ -\partial_x \pm i\partial_y & \end{pmatrix}\begin{bmatrix} u(v)_a \\ u(v)_b \end{bmatrix}, \tag{S2}$$

where $v_F = \sqrt{3}at/2$, and u_a, u_b (v_a, v_b) are the annihilation operators at Dirac point $\mathbf{K}_+$ ($\mathbf{K}_-$) for the corresponding sublattice. After introducing a uniform Kekulé modulation, the two Dirac points

$\mathbf{K}_\pm$ are folded to the center of the Brillouin zone. The Hamiltonian in the vicinity of the Γ point preserving only first-order linear terms is

$$H_\Gamma = \frac{3}{2}\left(u_b^\dagger, u_a^\dagger, v_a^\dagger, v_b^\dagger\right)\begin{pmatrix} & -iAt\partial_{\bar{z}} & \Delta & \\ -iAt\partial_z & & & \Delta \\ \bar{\Delta} & & & iAt\partial_{\bar{z}} \\ & \bar{\Delta} & iAt\partial_z & \end{pmatrix}\begin{pmatrix} u_b \\ u_a \\ v_a \\ v_b \end{pmatrix}, \tag{S3}$$

where $\Delta = \Delta_0 e^{i\phi_r}$, $\partial_z = i\partial_x - \partial_y$, and $A = \sqrt{3}a$ is the lattice constant of the uniform Kekulé lattice. A gap with a size proportional to Δ_0 opens due to the interaction introduced by the Kekulé modulation.

Zero modes of the Hamiltonian in Eq. (S3) can be obtained by solving the eigen equation

$$H\psi = 0, \tag{S4}$$

i.e.,

$$At\left(-\partial_x + i\partial_y\right)u_a + \Delta v_a = 0, \tag{S5a}$$

$$At\left(\partial_x + i\partial_y\right)u_b + \Delta v_b = 0, \tag{S5b}$$

$$\bar{\Delta}u_b + At\left(\partial_x - i\partial_y\right)v_b = 0, \tag{S5c}$$

$$\bar{\Delta}u_a + At\left(-\partial_x - i\partial_y\right)v_a = 0. \tag{S5d}$$

We can find that the conditions $u_a = \pm v_a^*$ and $u_b = \pm v_b^*$ are satisfied by the eigenvectors. Thus, the above equations can be simplified as

$$At\left(-\partial_x + i\partial_y\right)u_a \pm \Delta_0 e^{i(\phi_r - \phi')}u_a^* = 0, \tag{S6a}$$

$$At\left(\partial_x + i\partial_y\right)u_b \pm \Delta_0 e^{i(\phi_r - \phi')}u_b^* = 0, \tag{S6b}$$

where ϕ' is an undetermined parameter. For the BMLZMs, it depends on the details of the ϕ_r variation at the boundary.

b. Topological phase transition of the boundary bands

For a large enough system, the modulation phase at the boundary can be effectively regarded as locally uniform. To simplify our analysis, we start with the case with uniform-texture Kekulé modulation phase ϕ which only depends on the initial offset α_0, i.e., the $\boldsymbol{\theta_r}$ term is set as zero in

$\phi_{\boldsymbol{r}}$. As shown in Fig. S5a, we consider a ribbon lattice which is periodic in the x direction and large enough in the y direction. The outer normal of the upper armchair edge boundary is parallel to ΓK_{+} (the same as the boundaries in Fig. 1 of main text), while that of the lower armchair edge boundary is parallel to ΓK_{-}. As labeled by color dash lines in Fig. S5a, there are three types of terminals, denoted as Boundary I (black line), II (red line), and III (blue line). Take Boundary I as an example, we calculate the energy bands of the ribbon lattice terminated by Boundary I for ϕ with $k_x = 0$, as shown in Fig. S5b. The uniform-texture Kekulé modulation couples the two Dirac points at K and K' points, and a bulk gap whose size depends on the modulation strength opens[1-3]. Besides the bulk states (yellow shaded region), there are two boundary modes (dark green and brown bold lines) on each armchair boundary, and they form 1D Dirac points at $\phi = \pm 0.873\pi$ for the upper and lower boundary states, respectively. The boundary states for $\phi \neq \pm 0.873\pi$ always remain gapped for all k_x. We focus on the upper boundary hereafter.

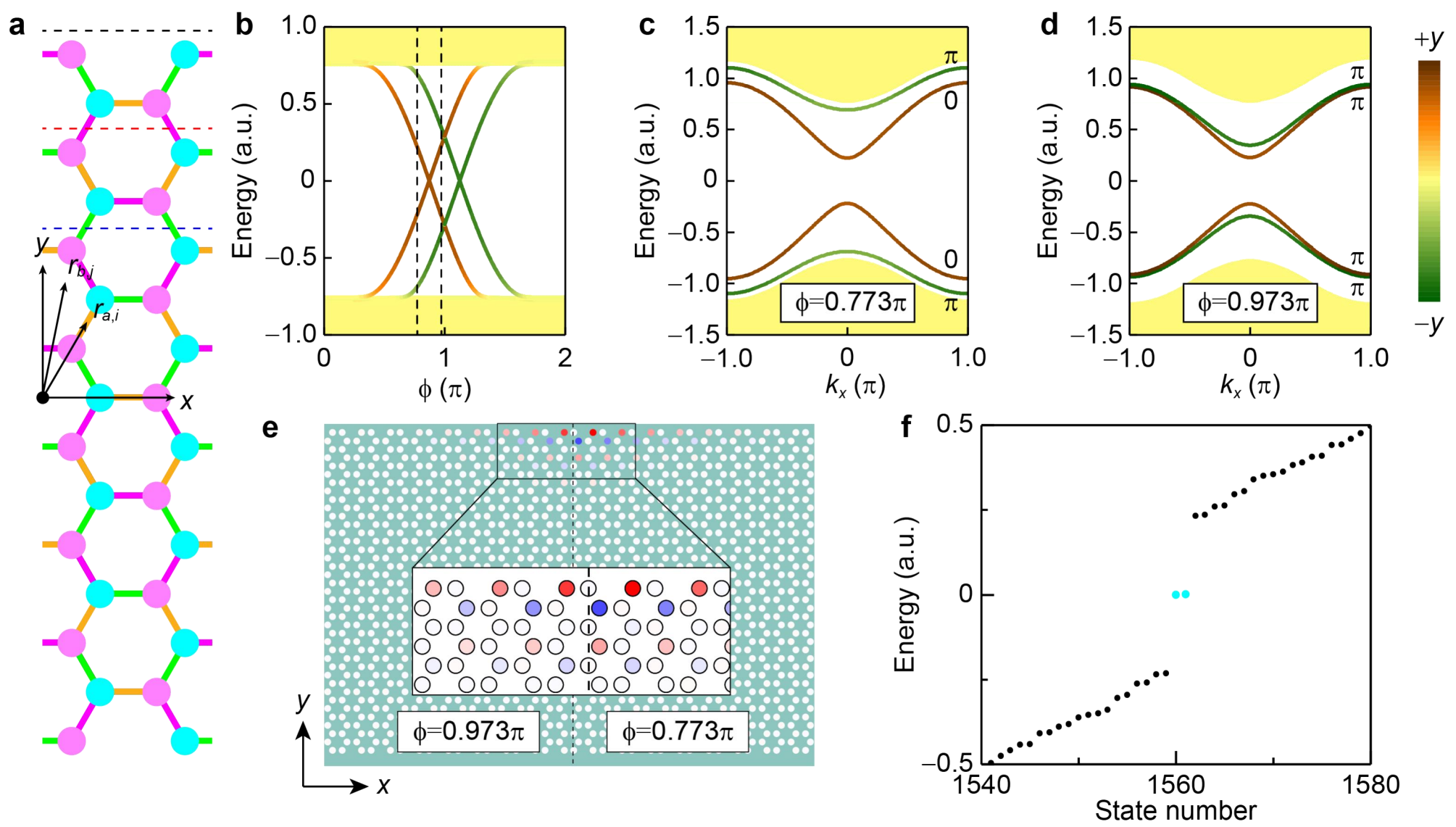

Fig. S5. | Topological phase transition of the boundary bands. a, A ribbon of the lattice with uniform Kekulé modulation denoted by ϕ. **b**, Energy bands of the ribbon structure terminated by Boundary I at $k_x = 0$ as a function of ϕ. **c**,**d**, Energy bands of the ribbon structure for k_x at $\phi = 0.773\pi$ (**c**) and $\phi = 0.973\pi$ (**d**), where the Zak phases of the boundary modes are also given. In (**b-d**), the color code represents the location of the eigenstates, brown (dark green) lines indicate the states at upper (lower) boundary and yellow regions denotes bulk states. **e**,**f**, A domain wall (**e**) formed between two regions with different uniform Kekulé modulation phases, with the

corresponding energy spectrum shown in (**f**). The cyan points represent the two domain-wall zero modes and the field distribution of one is shown in (**e**). In calculation, the periodic boundary conditions, with $t = 1$, and $\Delta_0 =- 1.5$, are applied in the *x* direction for both (**a**) and (**e**).

Figures S5c and S5d show the energy band of the ribbon lattice terminated by Boundary I for k_x with $\phi = 0.973\pi$ and $\phi = 0.773\pi$, respectively. These two ϕ values are labeled by the two vertical dashed lines in Fig. S5b, which are purposely chosen to be on different sides of the Dirac point of the upper boundary states. The Zak phases of the boundary bands are also shown in Figs. S5c and S5d, and clearly a (0, 0) to (π, π) topological phase transition occurs. Such a phase transition is similar to the well-known Jackiw-Rebbi mechanism[4], and here is quantization of Zak phase is ensured by the mirror symmetry along the *x* direction. Accordingly, a boundary domain wall state emerges at the domain wall of two regions with ϕ at two sides of the boundary Dirac point. To demonstrate this point, we calculate the band structure with a domain wall formed by uniform-texture Kekulé modulation phase $\phi = 0.973\pi$ and $\phi = 0.773\pi$, as shown in Fig. S5e. Periodic boundary condition is still imposed in the x direction, and the number of lattices in the y direction is finite while large enough. The corresponding energy spectrum of the lattice is shown in Fig. S5f. Two zero energy modes emerge in the band gap, corresponding to the boundary domain wall states. Note that there is no topology phase transition at the lower boundary, and hence these two zero energy modes are all localized at the upper boundary. One is localized at the middle domain wall and the other at the outer domain wall since a periodic boundary condition is applied. Figure S5e also shows the eigenfield of the zero-energy mode localized at the middle domain wall, which exhibits almost the same field distribution as the BMLZM in Fig. 1 of main text. Hence, the discussions above explain the topological origin and as well as the localization of the BMLZM at $\phi_r = 0.873\pi$. Similarly, for the ribbon lattice terminated by Boundary II (or III), two boundary modes also form 1D Dirac points at $\phi =\pm 0.206\pi$ ($\pm 1.540\pi$) for the upper and lower boundary, respectively. When assembling the triangular-shaped model terminated by Boundary II (or III), the BMLZM is localized at $\phi_r = 0.206\pi$ (1.540π).

c. Solution of the BMLZMs at a domain wall

Here, we proceed to derive the analytical solution of the domain-wall state of the BMLZM that satisfies the low-energy effective Hamiltonian. The solution is

$$u_a = e^{\pm\frac{\Delta_0 \cos(\phi_r-\phi')}{At}(x-x_0)} e^{\mp\frac{\Delta_0 \sin(\phi_r-\phi')}{At}(y-y_0)}, \tag{S7a}$$

$$u_b = e^{\mp\frac{\Delta_0 \cos(\phi_r-\phi')}{At}(x-x_0)} e^{\mp\frac{\Delta_0 \sin(\phi_r-\phi')}{At}(y-y_0)}, \tag{S7b}$$

here the $+/-$ sign depends on the boundary chosen, i.e., only the decaying waves from the domain boundary are allowed, x_0 (y_0) represents the position of the domain-wall (the upper boundary in the y direction), as also labeled in Fig. S6a. The full wavefunction is given by $\psi = u_a + v_a + u_b + v_b$. We first consider the solution at the edge boundary along the x direction. On the different side of the domain wall, $\cos(\phi_r - \phi')$ should have opposite signs to give rise a localized mode along the x direction. In the case of Fig. 2(e) in the main text, $\phi_{\text{right}} < \phi_{\text{close}} < \phi_{\text{left}}$, where $\phi_{\text{close}} = 0.873\pi$ is the band closing modulation phase. Suppose $\phi_{\text{left}} = \phi_{\text{close}} + \delta\phi$, $\phi_{\text{right}} = \phi_{\text{close}} - \delta\phi'$, where $\delta\phi$, $\delta\phi'$ are positive numbers. Then the condition become $\cos(\phi_{\text{close}} - \delta\phi - \phi')$ and $\cos(\phi_{\text{close}} + \delta\phi' - \phi')$ should have opposite signs. Then, we have

$$\phi_{\text{close}} - \phi' = \pm\frac{\pi}{2}, \tag{S8}$$

Thus, $\phi' = \phi_{\text{close}} \pm \frac{\pi}{2}$. The solution at the boundary is

$$u_a = e^{\mp\frac{\Delta_0 \sin(\phi_r-\phi_{\text{close}})}{At}(x-x_0)} e^{\mp\frac{\Delta_0 \cos(\phi_r-\phi_{\text{close}})}{At}(y-y_0)}, \tag{S9a}$$

$$u_b = e^{\pm\frac{\Delta_0 \sin(\phi_r-\phi_{\text{close}})}{At}(x-x_0)} e^{\mp\frac{\Delta_0 \cos(\phi_r-\phi_{\text{close}})}{At}(y-y_0)}. \tag{S9b}$$

Considering that the mode should decay along the y direction (and $\Delta_0 < 0$ in our calculation), the solution should be

$$u_a = e^{-\frac{\Delta_0 \sin(\phi_r-\phi_{\text{close}})}{At}(x-x_0)} e^{-\frac{\Delta_0 \cos(\phi_r-\phi_{\text{close}})}{At}(y-y_0)}, \tag{S10a}$$

$$u_b = e^{\frac{\Delta_0 \sin(\phi_r-\phi_{close})}{At}(x-x_0)} e^{-\frac{\Delta_0 \cos(\phi_r-\phi_{\text{close}})}{At}(y-y_0)}, \tag{S10b}$$

Only u_a can be normalized in this case. Thus, the field only exists in Λ_a sublattice, which is consistent with the numerical result.

The solution has a different boundary condition at the domain wall along the y direction, i.e.,

continuity boundary condition normal to the domain wall. Thus,

$$-\Delta_0 \sin\left(\phi_{\text{left}} - \phi'\right) = -\Delta_0 \sin\left(\phi_{\text{right}} - \phi'\right), \tag{S11}$$

Then,

$$\phi' = \frac{\alpha_{\text{left}} + \alpha_{\text{right}} - \pi}{2}. \tag{S12}$$

The solution thus becomes

$$u_a = e^{\mp\frac{\Delta_0}{At}\sin\left(\phi_r - \frac{\alpha_{\text{left}}+\alpha_{\text{right}}}{2}\right)(x-x_0)} e^{\mp\frac{\Delta_0}{At}\cos\left(\phi_r - \frac{\alpha_{\text{left}}+\alpha_{\text{right}}}{2}\right)(y-y_0)}, \tag{S13a}$$

$$u_b = e^{\pm\frac{\Delta_0}{At}\sin\left(\phi_r - \frac{\alpha_{\text{left}}+\alpha_{\text{right}}}{2}\right)(x-x_0)} e^{\mp\frac{\Delta_0}{At}\cos\left(\phi_r - \frac{\alpha_{\text{left}}+\alpha_{\text{right}}}{2}\right)(y-y_0)}, \tag{S13b}$$

Also, only u_a can be normalized in this case.

d. Comparison of the domain wall solution of the BMLZM with numerical results

In this section, we compare the $\boldsymbol{k}\cdot\boldsymbol{p}$ Hamiltonian solution Eq. (S7) with the numerical results obtained from the tight-binding model along both the boundary and the domain wall. We sample the numerically solved eigenfield of the BMLZM along four representative lines in the vicinity of the domain wall. Two of them are at the boundary and along the x direction, while the other two are at the domain wall and along the y direction, as sketched in Fig. S6a. The decaying follows the relation $\psi(\Delta r) = \psi_0 e^{\beta \Delta r}$ ($\beta\Delta r < 0$), where Δr is the distance between the site and domain wall and β is the decay factor. Since the $\boldsymbol{k}\cdot\boldsymbol{p}$ Hamiltonian only preserves the first-order terms, we need to set Δ_0 to be small enough to make higher-order effects negligible. Here, in the numerical calculation, we choose $\Delta_0 = -0.3$, $\phi_{\text{left}} = 0.813\pi$, $\phi_{\text{right}} = 0.933\pi$. Under this change of Δ_0, the boundary band closing point on the ΓK_+ boundary is changed to $\phi_{\text{close}} = 0.8424\pi$.

The field distribution of the BMLZM in the vicinity of the domain wall is plotted in Fig. S6b. To obtain the decay factor of the tight-binding model, we sample one point per Kekulé unit cell to obtain the field amplitudes. We then divide these amplitudes with the maximum of them (actually the amplitude of the point closest to the domain wall) and take the logarithm. The logarithmic amplitudes for the points on x_{left} and y_{left} lines are plotted in Figs. S6c,d, respectively. By linear fitting the numerical data from the tight-binding model, we obtain the decay factor (slope of the linear fitting line) and then compare them with the value predicted by $\boldsymbol{k}\cdot\boldsymbol{p}$ Hamiltonian. The

results are shown in Table. S1 where the consistency is observed.

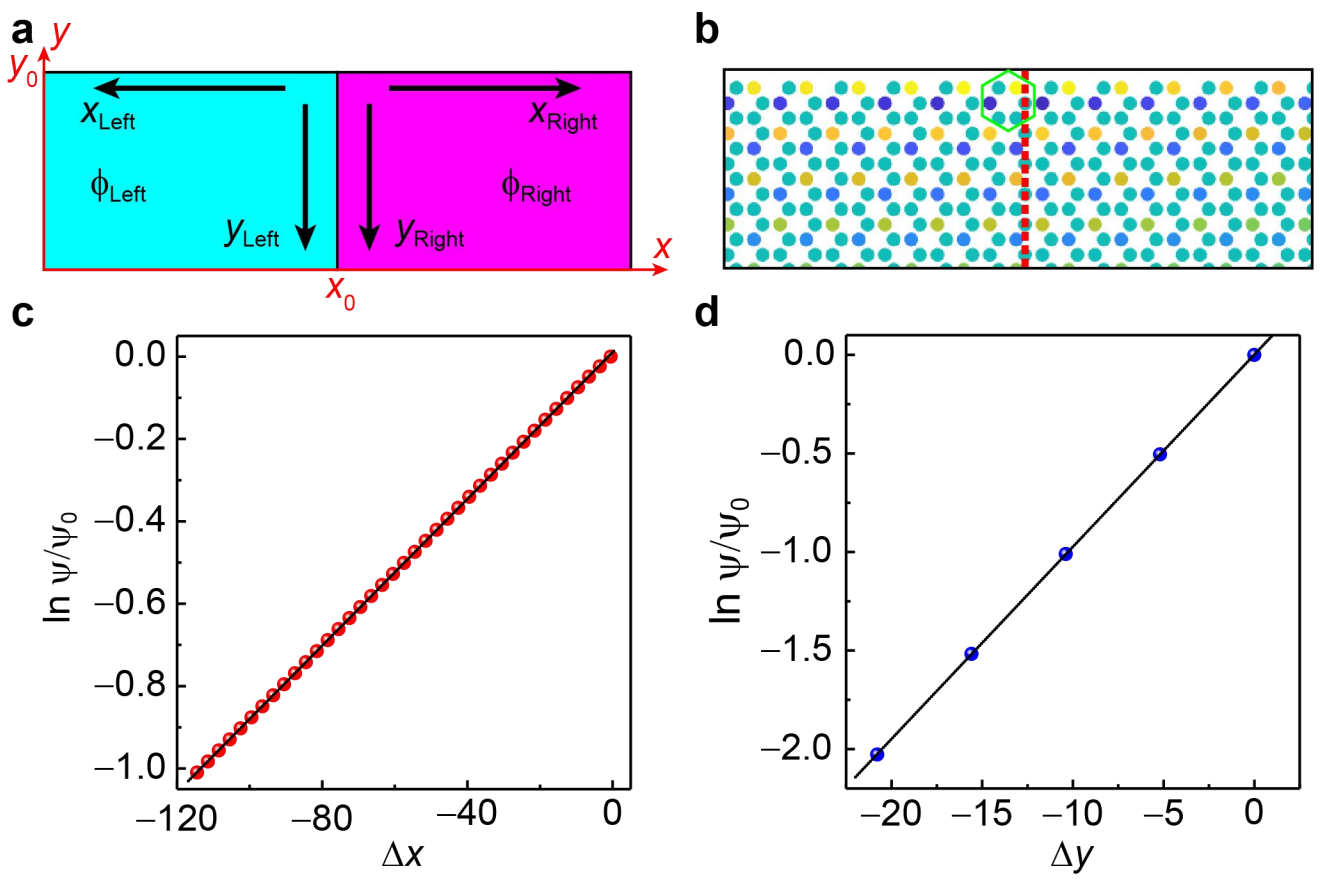

FIG. S6. | Comparing the $\boldsymbol{k}\cdot\boldsymbol{p}$ Hamiltonian solution and the numerical result. a, Sketch showing the sampling points location and two domains. **b**, Field distribution of the BMLZM in the vicinity of the domain wall. The green hexagon indicates the unit cell of the uniformed Kekulé lattice. **c**, Logarithmic amplitude versus the distance from the domain wall. **d**, Logarithmic amplitude versus the distance from the boundary. The fields on the left domain are plotted in (**c**) and (**d**) along the *x* and *y* directions, respectively. The size of the domain is chosen to be large enough to keep the logarithmic behavior in (**c**) and (**d**). $\Delta_0 = 0.3$, $\alpha_{\text{left}} = 0.813\pi$, $\alpha_{\text{right}} = 0.933\pi$, and the other parameter used are the same as Fig. S5.

Line	Numerical decay factor	$\boldsymbol{k}\cdot\boldsymbol{p}$ Hamiltonian predicted decay factor
x_{left}	0.0089	0.0092
x_{right}	-0.0287	-0.0281
y_{left}	0.09753	0.09823
y_{right}	0.0977	0.09823

TABLE. S1 Decay factors obtained from the numerical solution of the tight-binding model and that obtained from the effective $\boldsymbol{k}\cdot\boldsymbol{p}$ Hamiltonian.

e. Annihilation of the BMLZM with the CMLZM

The topological charges of the BMLZMs and CMLZMs are of the same type. When a BMLZM and a CMLZM carry opposite charges, they can couple with each other when the size of the system

is not large enough. When the CMLZM is moved to be close enough to the BMLZM, these two zero energy modes can annihilate each other, and their energy split. In Fig. S7a, we display the eigen energy spectrum as a function of the distance d_0 between the vortex center and the BMLZM. When $d_0 = l_0$ (with l_0 being the distance between the center of the lattice and the BMLZM), the distance between the two MLZMs is large enough, the energy splitting is small. Here the wavefunction is clearly distinguishable as shown in Fig. S7b. As the vortex center (so as the CMLZM) moves towards the position of the BMLZM, the coupling between these two MLZMs increases, and the energy splitting becomes observable. The typical eigenfield distributions of the modes denoted by the cyan dots in Fig. S7a (the negative energy ones) for a few representative locations of the vortex centers are shown in Figs. S7b-f. When the CMLZM is close enough with the BMLZM, these two MLZMs annihilate with each other.

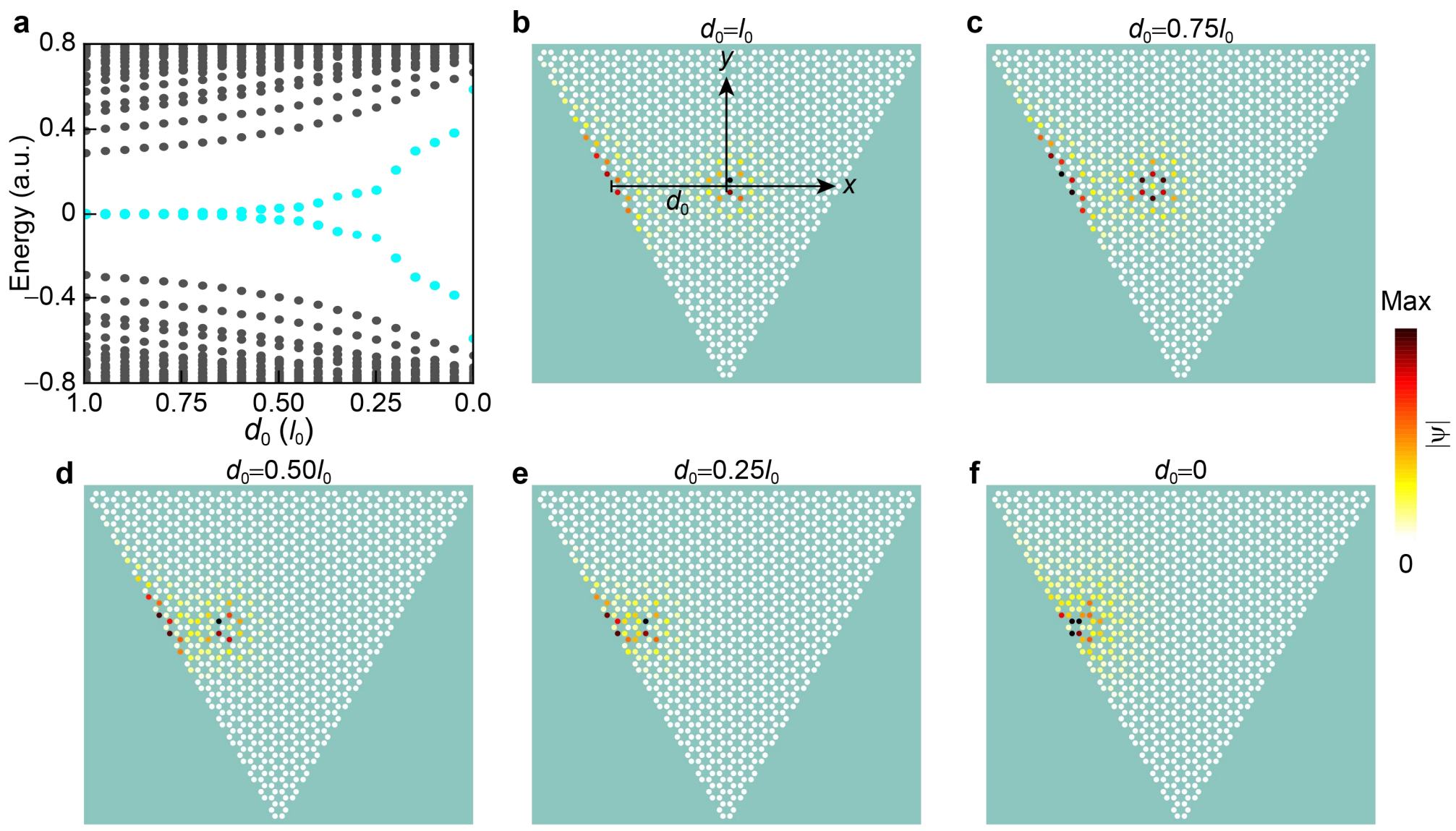

FIG. S7 | Annihilation of the BMLZM with the CMLZM. a, Eigen energy spectrum versus locations of the vortex core. Cyan dots mark the Majorana-like modes. **b**-**f**, Field amplitude distributions of the hybrid Majorana-like mode with negative energy. The distance between the vortex center and the BMLZM is defined as d_0, and the distance between the center of the lattice and the BMLZM is labeled as l_0. In **b**-**f**, we gradually change the position of the vortex center while keeping other parameters unchanged. In calculation, $t = 1$, $\Delta_0 =- 1.5$, and $\alpha_0 = 0$.

f. Annihilation of the two CMLZMs

This section considers the annihilation of two CMLZMs with opposite charges. We start from two CMLZMs whose vortex centers are separated by a distance d_1 larger than twice the effective size of a single CMLZM, and then gradually decrease this distance. In Fig. S8a, we first show the eigenenergy spectrum as a function of d_1. When $d_1 = 6\sqrt{3}a$, the two CMLZMs are far apart, with clearly separated spatial field distributions, as shown in Fig. S8b, and their eigenenergies are nearly degenerate.

As the vortex centers, and hence the CMLZMs, move closer to each other, their mutual coupling increases and the eigenenergies split into $\pm\varepsilon$, as shown in Figs. S8c, d. The corresponding eigenstates can be interpreted as even and odd combinations of the two original CMLZMs. When the vortex centers become very close, for example at $d_1/\sqrt{3}a = 0.01$ in Fig. S8e, the eigenfield gradually loses the characteristic localization of the CMLZMs and becomes spatially extended.

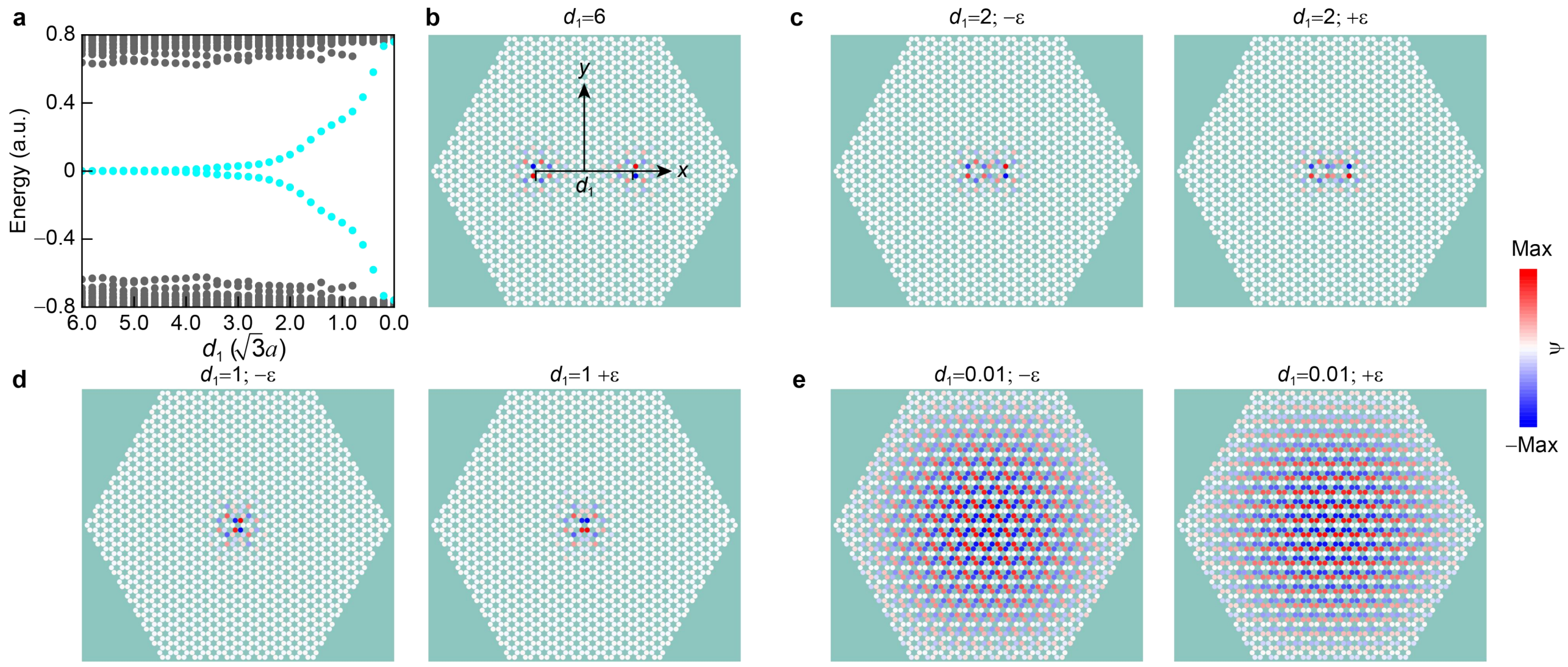

FIG. S8 | Annihilation of two CMLZMs. a, Eigenenergy spectrum as a function of the distance d_1 between the vortex cores, as marked in (**b**). Cyan dots indicate the energies of the two CMLZM-like modes. **b**–**d**, Eigenfield distributions of the CMLZM modes for different separations: $d_1 = 6\sqrt{3}a$ in **b**, $d_1 = 2\sqrt{3}a$ in **c**, and $d_1 = \sqrt{3}a$ in **d**. **e**, Eigenfield distribution when the vortex centers become very close, with $d_1 = 0.01\sqrt{3}a$. In **c**–**e**, $+\varepsilon$ and $-\varepsilon$ label the eigenstates with positive and negative energies, respectively. In the calculations, except for the shape of the PC and the locations of the MLZMs, all other unspecified parameters are the same as those in Fig. S7.

S-III. The position of BMLZM for different α_0

Figure 1e of the main text shows the eigen spectrum of the system for different α_0. In Figs. 1d,f-h of the main text, four typical eigenfield distributions of BMLZMs are given. As we change α_0, the position of the BMLZM shifts accordingly with its center roughly at the position where the modulation phase $\phi_r = 0.873\pi$. Figures S9a-h show the field distributions of one zero energy mode at other few α_0. The lower-left insets depict the locations of the BMLZMs, where the half-red-half-blue disk in Fig. S9f highlights the π phase jump at the corner, and the color arrows indicate the directions of evolution with the increasing of α_0. The lower-right insets show the distributions of the modulation phase ϕ_r, where the black lines mark the position with $\phi_r = 0.873\pi$, which always match well with the positions of the BMLZM. In addition, as α_0 increases from 0 to 2π, both the BMLZMs and the CMLZM (shown in Part S-IV) acquire an additional π phase.

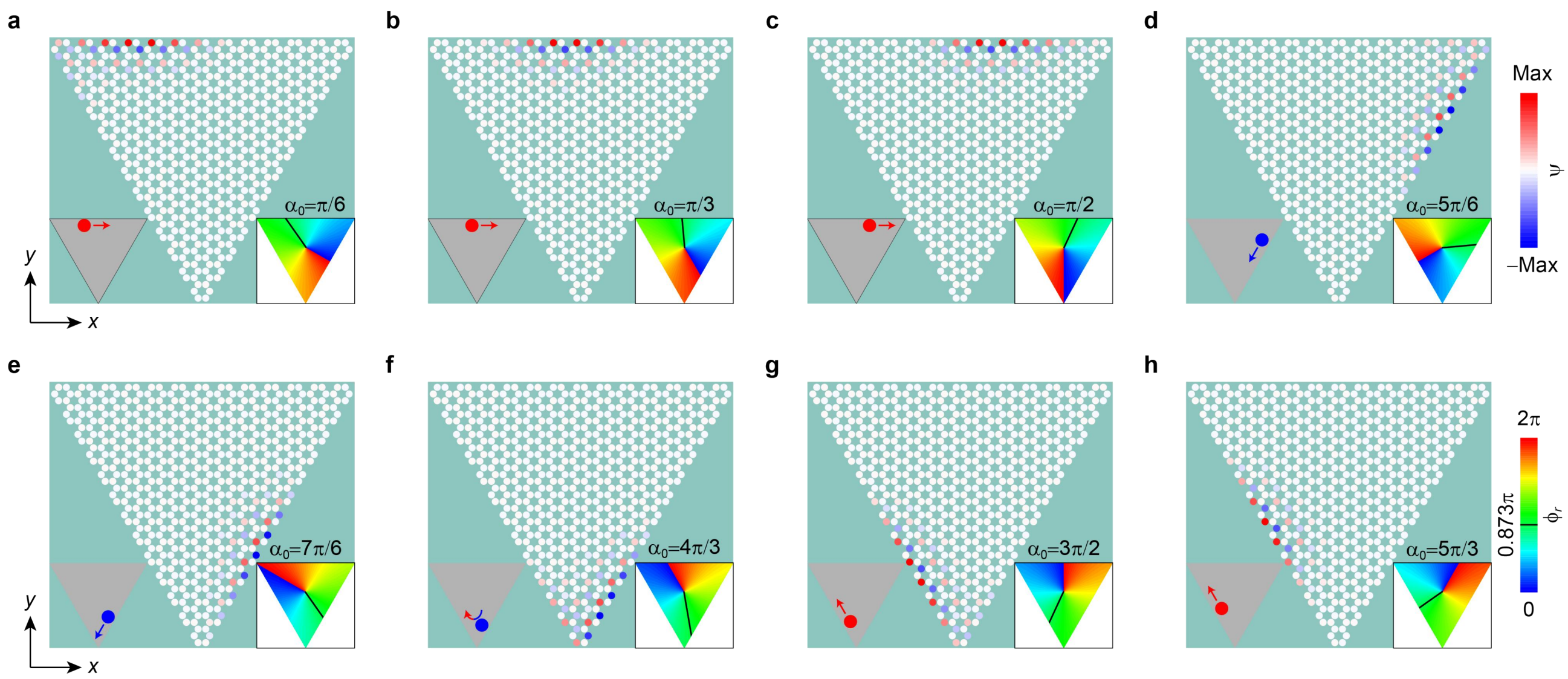

FIG. S9. | The position of BMLZMs for different α_0. a-h, Eigenfield distributions of the BMLZM with $\alpha_0 = \pi/6$ (**a**), $\pi/3$ (**b**), and $\pi/2$ (**c**), $5\pi/6$ (**d**), $7\pi/6$ (**e**), $4\pi/3$ (**f**), $3\pi/2$ (**g**), and $5\pi/3$ (**h**), respectively. The lower-left insets depict the locations of the MLZMs, where the half-red-half-blue disk in (**f**) highlights the π phase jump at the corner, and the colored arrows indicate the directions of evolution with the increasing of α_0. The lower-right insets show the distributions of the modulation phase ϕ_r, and the black lines mark the position with $\phi_r = 0.873\pi$. Except for α_0, all the other parameters are the same as Fig. 1 of the main text.

In Fig. 1 of the main text and Fig. S9, the lattice contains only one kind of armchair boundary (Boundary I), whose outer normal is along the ΓK_+ direction, thus the localization of the BMLZM is consistently centers at $\phi_r = 0.873\pi$. Here, we discuss the case when the outer normal of the boundaries consists of both ΓK_+ and ΓK_-. In this case, the lattice contains six armchair boundaries, with three ΓK_+ boundaries and three ΓK_- boundaries. As shown in Fig. S10a, we calculated the eigen energy spectrum as a function of the initial offset α_0. Same as Fig. 1e of main text, there are two zero energy MLZMs for all α_0. However, the eigenfields of the BMLZM become a little bit more complex as now we have different outer boundaries.

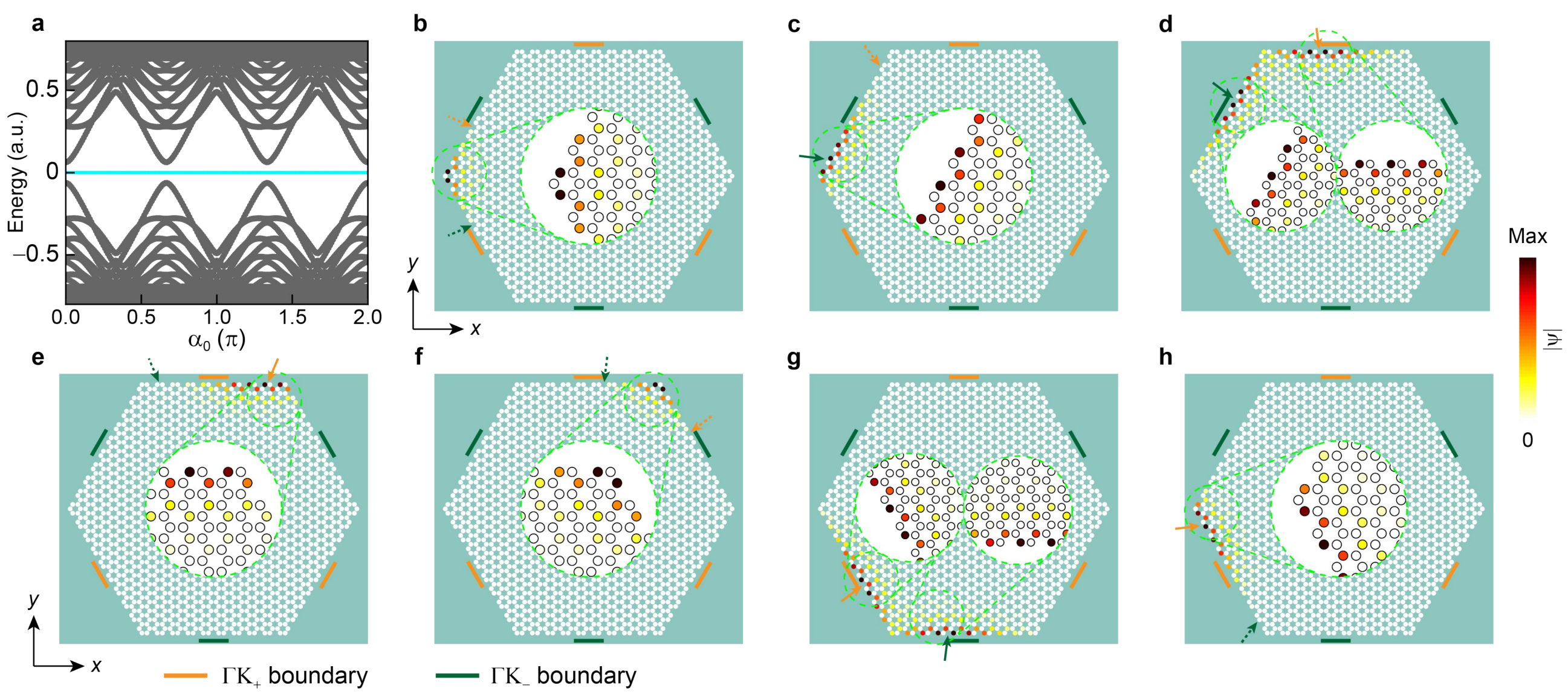

FIG. S10. | Evolution of the BMLZMs in case when both ΓK_+ and ΓK_- boundaries present. a, Eigen energy spectrum as a function of initial offset α_0. Cyan dots represent the two MLZMs, and the dark gray dots represent the bulk modes. **b-h**, Field amplitudes for the zero energy state with $\alpha_0 = 0,\ \frac{1}{6}\pi,\ \frac{2}{6}\pi,\ \frac{3}{6}\pi,\ \frac{4}{6}\pi,\ \frac{10}{6}\pi$, and $\frac{11}{6}\pi$, respectively. The orange and dark green lines mark the ΓK_+ and ΓK_- boundaries, respectively. The orange and dark green arrows denote the location with $\phi_r = 0.873\pi$ and $\phi_r =- 0.873\pi$, respectively. **c**,**e**,**h**, depict type I BMLZMs, **d**,**g**, depict type II BMLZMs, **b**,**f**, depict type III BMLZMs. In calculation, $N_{\Gamma K_+} = N_{\Gamma K_1} = 10$, $t = 1$, and $\Delta_0 =- 1.5$.

In Figs. S10b-h, the eigenfield amplitudes distributions of one of the MLZMs are shown for $\alpha_0 = 0,\ \frac{1}{6}\pi,\ \frac{2}{6}\pi,\ \frac{3}{6}\pi,\ \frac{4}{6}\pi,\ \frac{10}{6}\pi$, and $\frac{11}{6}\pi$, respectively. Here the orange and dark green lines mark the

ΓK$_+$ and ΓK$_-$ boundaries, respectively. As discussed in Fig. S5, the BMLZM locates at $\phi_r = 0.873\pi$ (-0.873π) for the ΓK$_+$ (ΓK$_-$) boundary and decays away from that point (except a few cases as discussed below). Depending on the position of these critical modulation phases, the wave function of the BMLZM can be classified into three different types. Type I, as shown in Figs. S10c,e,h, when either $\phi_r = 0.873\pi$ (pointed by the orange arrows) is on the ΓK$_+$ boundary or $\phi_r =- 0.873\pi$ (pointed by the green arrows) is on the ΓK$_-$ boundary, the wavefunction of BMLZM is similar as Fig. S5. Type II appears when $\phi_r = 0.873\pi$ is on the ΓK$_+$ boundary while $\phi_r =- 0.873\pi$ is also on the ΓK$_-$ boundary, as shown in Figs. S10d,g. According to our analysis in Fig. S5, the wavefunction of the BMLZM emerges at both $\phi_r =\pm 0.873\pi$. However, the wavefunction meets the corner before it decays to zero. As a consequence, the BMLZM becomes a two-peak function at the corner. Note here, there is still only one BMLZM though the wave function spread widely. Type III appears when neither $\phi_r = 0.873\pi$ is on the ΓK$_+$ boundary nor $\phi_r =- 0.873\pi$ is on the ΓK$_-$ boundary, as shown in Figs. S10b,f. The presence of the BMLZM can be understood from the picture of adiabatic parameter tuning process. Different from type II, the wavefunction of type III is pretty localized at the corner where $\phi_r \approx \pi$. Same as Fig. 1 of main text and Fig. S5, the BMLZM acquires a π phase as α_0 increases from 0 to 2π. As α_0 adiabatically increases from 0 to 2π, the BMLZM moves around the six boundaries of the lattice in Fig. S10. The BMLZM gets no additional phase when it moves from ΓK$_-$ boundary to ΓK$_+$ boundary. However, it acquires a π phase when it moves from ΓK$_+$ boundary to ΓK$_-$ boundary. Or equivalently speaking, as α_0 adiabatically increases from 0 to 2π, the BMLZM goes through three processes like Type III → Type I → Type II → Type I → Type III as shown in Figs. S10b-f, and the BMLZM has a π phase difference between every two processes.

S-IV. Braiding between a BMLZM and a CMLZM

For the system considered in Fig. 1 of the main text and Fig. S8, both a CMLZM and a BMLZM are present. As discussed in Fig. 1, when α_0 increases from 0 to 2π, the BMLZM acquires a π phase factor. In fact, during this process, the CMLZM also acquires an additional π phase. To illustrate this point, Fig. S11 displays the eigenfield distributions of the BMLZM and CMLZM at different values

of α_0. To highlight the phase accumulation during this process, the lower-left insets show the phase distribution along the boundary and at the center of the CMLZM. Here, the red and blue disks denote the BMLZM with phases 0 and π, respectively, the hexamers denote the CMLZM, and the colored arrows indicate the evolution direction of the BMLZM. All other parameters are the same as those in Fig. 1. Clearly, as α_0 increases from 0 to 2π, the BMLZM rotates clockwise along the boundary, eventually returns to its initial position, and acquires an additional π phase. Meanwhile, the CMLZM also acquires an additional π phase, as shown by comparing Figs. S11a and S11m.

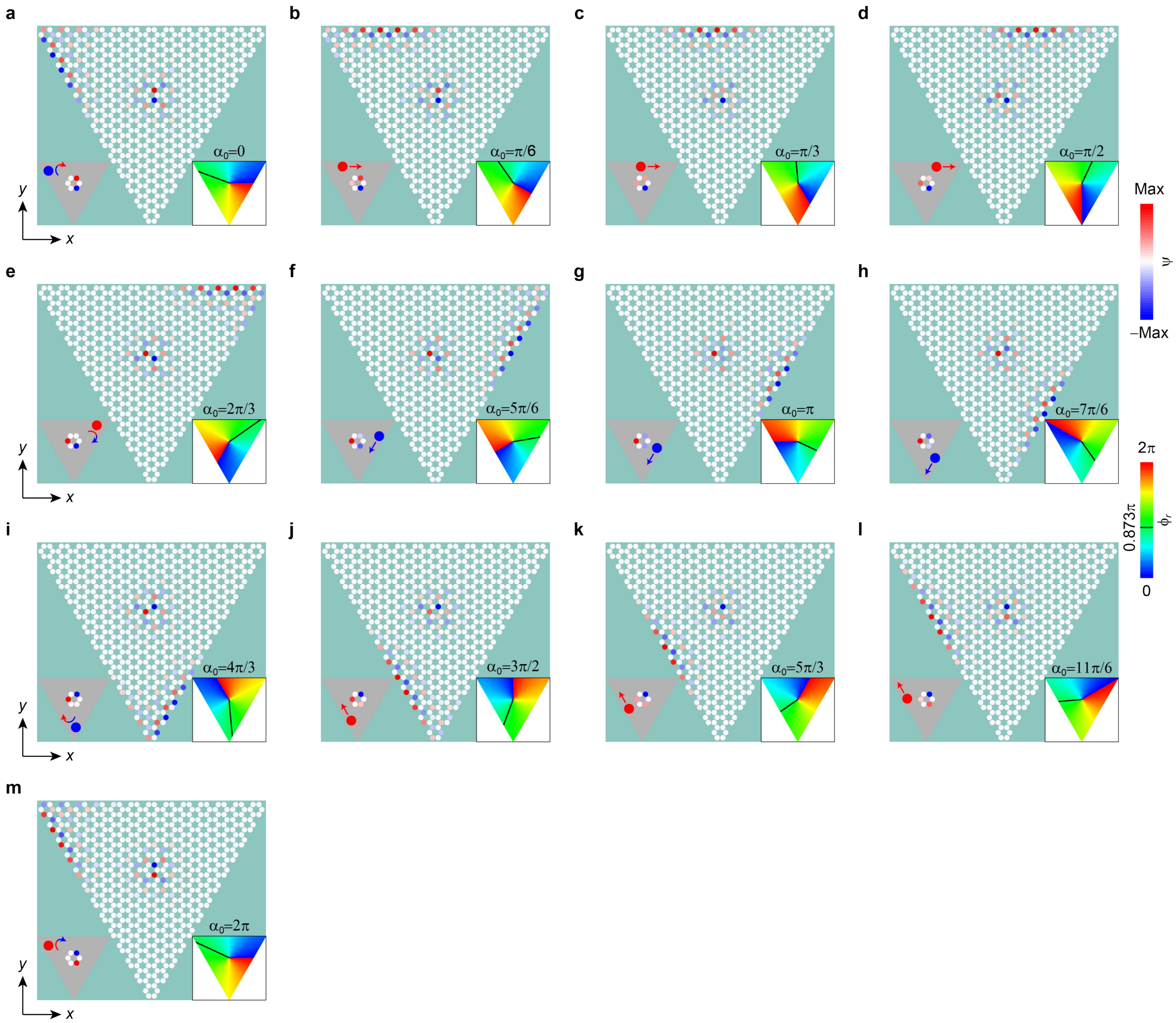

FIG. S11. | BMLZM braided with the CMLZM as α_0 is gradually increased. a-m, Eigenfield distributions of the BMLZM together with that of the CMLZM for $\alpha_0 = 0$ (**a**), $\pi/6$ (**b**), $\pi/3$ (**c**), $\pi/2$ (**d**), $2\pi/3$ (**e**), $5\pi/6$ (**f**), π (**g**), $7\pi/6$ (**h**), $4\pi/3$ (**i**), $3\pi/2$ (**j**), $5\pi/3$ (**k**) $11\pi/6$ (**l**),

and 2π (**m**), respectively. The lower-left insets depict the locations of the BMLZM and the phase distribution along the boundary and at the center of the CMLZM, while the colored arrows indicate the evolution direction of the BMLZM as α_0 increases. The lower-right insets show the distributions of the modulation phase ϕ_r, and the black lines mark the position with $\phi_r = 0.873\pi$. Except for α_0, all the other parameters are the same as Fig. 1 of the main text.

S-V. Continuity boundary condition of BMLZMs at lattice corners

The wavefunction of the BMLZM satisfies the continuity boundary condition when its position adiabatically moves across corners of model. In Fig. S12, we plot the eigenfield distributions of the BMLZMs at boundaries on both sides of a corner and at the corner. Here we consider the situation when the BMLZM moves clockwise across the upper-right corner. The situations for the other two corners are similar. In Fig. S12a, the wavefunction is positive at the outermost sites and negative at the next-outermost sites. When the BMLZM moves to a corner, the wave function reverses its sign on different sides of the corner, i.e., across the corner, the wavefunction is negative at the outermost sites and positive at the next-outermost sites. Such a sign change is mandated by the tight-binding Hamiltonian, specifically by the coupling relation between the two lattice sites adjacent to the corner. After the BMLZM moves across the corner, its wavefunction keeps being negative at the outermost sites as shown in Fig. S12c. Thus, the wavefunction of BMLZMs at the outermost sites and next-outermost sites change their signs when crossing each corner.

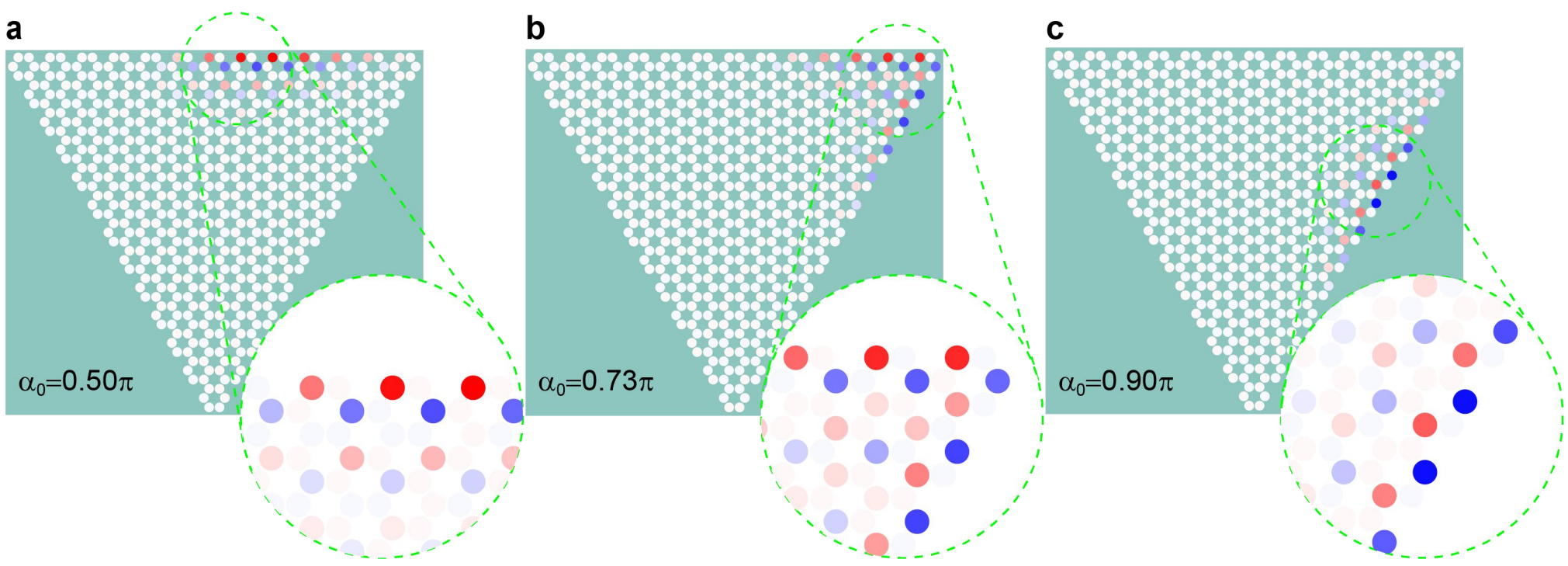

FIG. S12. | Continuity of the wavefunction of BMLZMs at lattice corners. a-c, Eigenfield distributions of the BMLZM at two adjacent boundaries (**a** and **c**) and the corresponding corner (**b**), as α_0 is gradually increased.

S-VI. CMLZM and BMLZM in discrete phononic crystals

In the main text, we implement a phononic crystal (PC) structure (see Fig. S13a) to realize the MLZMs in acoustic waves, and provide the eigenfrequency of BMLZM for different values of α_0. The complete eigenfrequency spectra of the triangular-shaped PC as a function of α_0 is shown in Fig. S13b, which is in overall agreement with the result from the tight-binding model presented in Fig. 1e of the main text. The cyan hollow circles and solid dots represent the eigenenergy of the CMLZMs and BMLZMs, respectively, while the black dots denote those of the bulk states. Two 'zero-energy' modes emerge within the bulk band gap, however there is a noticeable frequency splitting. This frequency splitting arises from unavoidable long-range couplings and finite-size effects that break the chiral symmetry in the real PC[6,7]. It can be mitigated by designing narrower coupling tubes and employing larger samples. To further validate the performance of the PCs, we present the simulated eigenfield distributions of CMLZM (~frequency of 7.18 kHz) and BMLZM (~frequency of 7.14 kHz) in Figs. S13c-e and Figs. S13f-h, respectively. As α_0 increases, the CMLZMs are localized at the center of the vortex all the time. In contrast, the position of BMLZM is dependent on values of α_0, and its wavefunction undergoes a π phase reversal as it crosses the corner of PC (Fig. S13g). Additionally, the acoustic field of two modes is only located on two distinct sublattices.

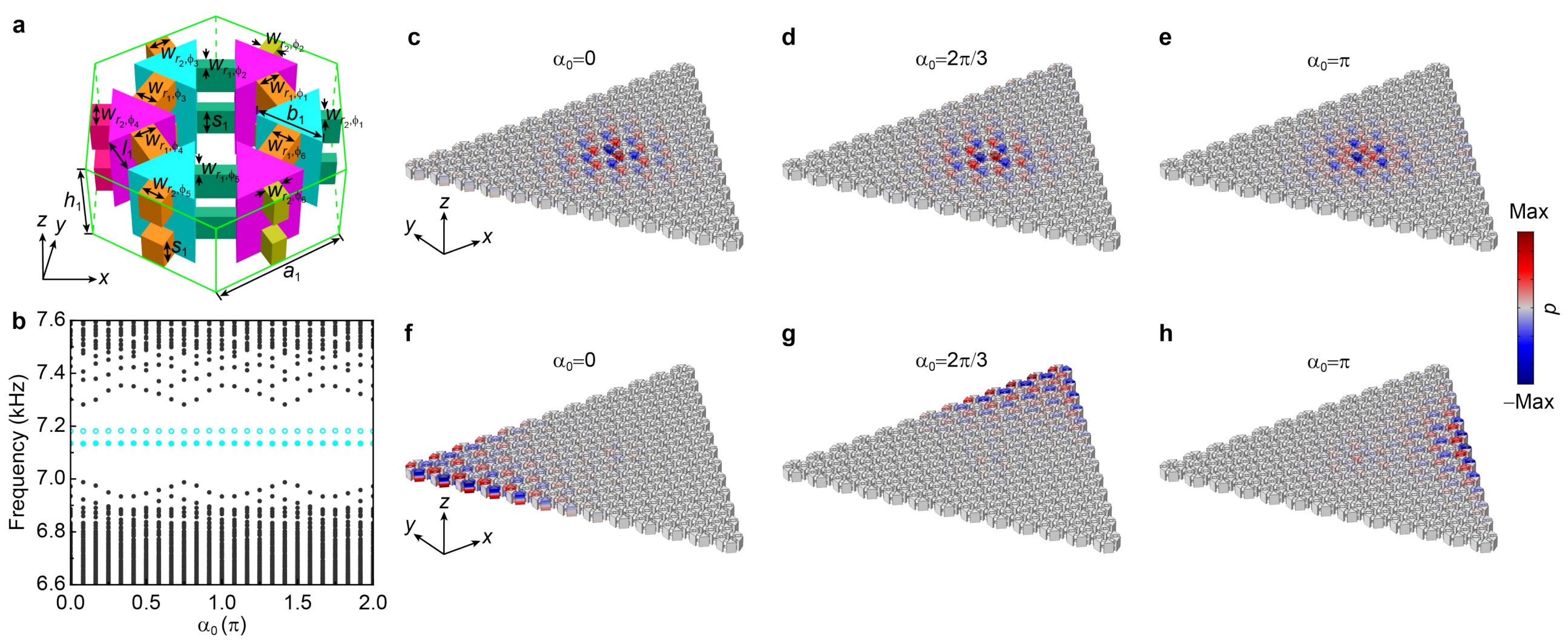

FIG. S13. | CMLZM and BMLZM in PCs. a, Schematic of a unit cell. The widths $w_{r,i}$ of the

coupling tubes are modulated according to the expression $w_{r,i} = (0.81 + \delta w_{r,i})$ cm, where $\delta w_{r,i} = \Delta_w e^{i(\boldsymbol{K}_+ \cdot \boldsymbol{s}_i + 2\boldsymbol{K}_+ \cdot \boldsymbol{r} + \phi_i)}/3 + \mathrm{c.c.}$, with $\Delta_w = 0.32$. **b**, Eigenfrequency spectra of the triangular-shaped PC for different values of α_0. The cyan hollow circles and solid dots denote the CMLZMs and BMLZMs, respectively, while the black dots represent the bulk states. **c-e**, Simulated eigenfield distributions of the CMLZMs (~frequency of 7.18 kHz) for $\alpha_0 = 0$ (**c**), $2\pi/3$ (**d**), and π (**e**). **f-h**, Simulated eigenfield distributions of the BMLZMs (~frequency of 7.14 kHz) for $\alpha_0 = 0$ (**f**), $2\pi/3$ (**g**), and π (**h**).

To elucidate the topological origin of BMLZM in PC, we also consider a ribbon lattice which is periodic in the x direction and large enough in the y direction, as illustrated in Fig. S14a. This ribbon lattice is subjected to the uniform-texture Kekulé modulation phase ϕ, which depends on the initial offset α_0. The outer normal of the upper armchair edge boundary is also parallel to ΓK_+ (the same as the boundaries in Fig. 1 of main text), while that of the lower armchair edge boundary is parallel to ΓK_-. Figures S14b and S14c show the projected bands of the ribbon PC for k_x with $\phi = 0.773\pi$ and $\phi = 0.973\pi$, respectively. Here the dark solid red (gray) lines represent the dispersion of the boundary modes localized on the upper (lower) boundary. These two ϕ values are purposely chosen to be on different sides of the Dirac point of the upper boundary states. The Zak phases of the boundary bands are also calculated and labeled, revealing a topological phase transition from (0, 0) to (π, π) that is linked to the well-known Jackiw-Rebbi mechanism.

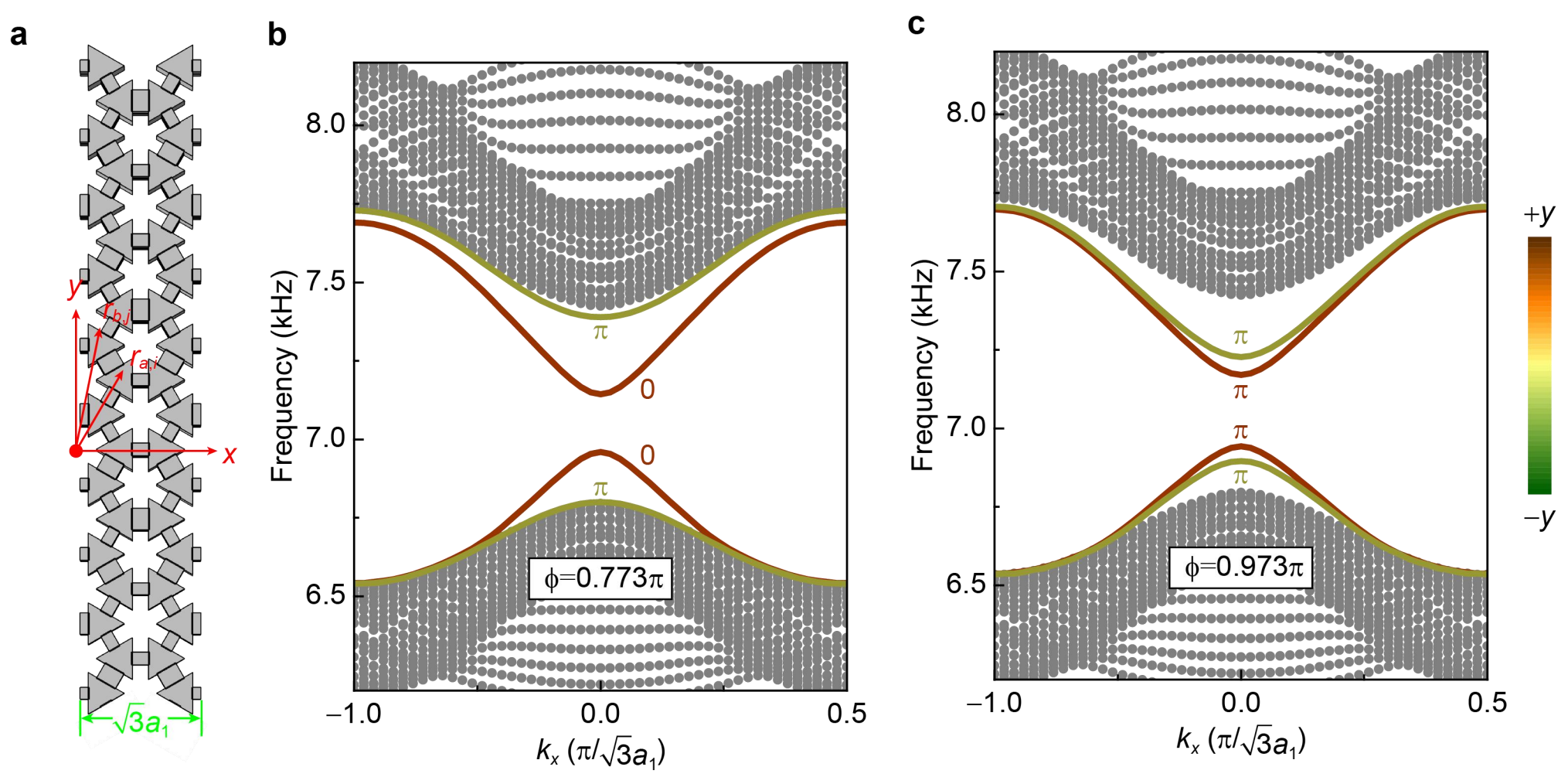

Fig. S14. | Topological phase transition of the boundary bands in PC. a, A ribbon geometry of

PCs with uniform Kekulé modulation denoted by ϕ. **b**,**c**, Projected band structures of the ribbon for versus k_x at $\phi = 0.773\pi$ (**b**) and $\phi = 0.973\pi$ (**c**), where the Zak phases of the boundary modes are also given. The dark red (gray) lines indicate the states at upper (lower) boundary and dots denotes bulk states. In calculation, the periodic boundary conditions are applied in the x direction, while the ribbon structure is thick enough in the y direction (the number of units is 20).

S-VII. Response spectra and field distributions of PCs containing one BMLZM

In the main text, we implement a 3D PC to demonstrate the adiabatic evolution of the BMLZMs, which is constructed by stacking discrete PCs with weak interlayer coupling along the z direction. Here, we measure the response spectra at $\phi_r = 0.873\pi$ on different layers to obtain the frequency of the BMLZMs in the real 3D PC. Two loudspeakers (with opposite phase) are positioned at the boundary of the second layer to excite the BMLZMs in each layer. As shown in Figs. S15a-l, we present the measured response spectra in the 3D PC for all the layers. These response spectra exhibit an intensity peak centered at 7.23 kHz slightly higher than the 7.14 kHz observed in the discrete PCs, and are normalized by the peak intensity. This result confirms that the frequency of the BMLZMs experiences a negligible shift due to introduction of the interlayer coupling.

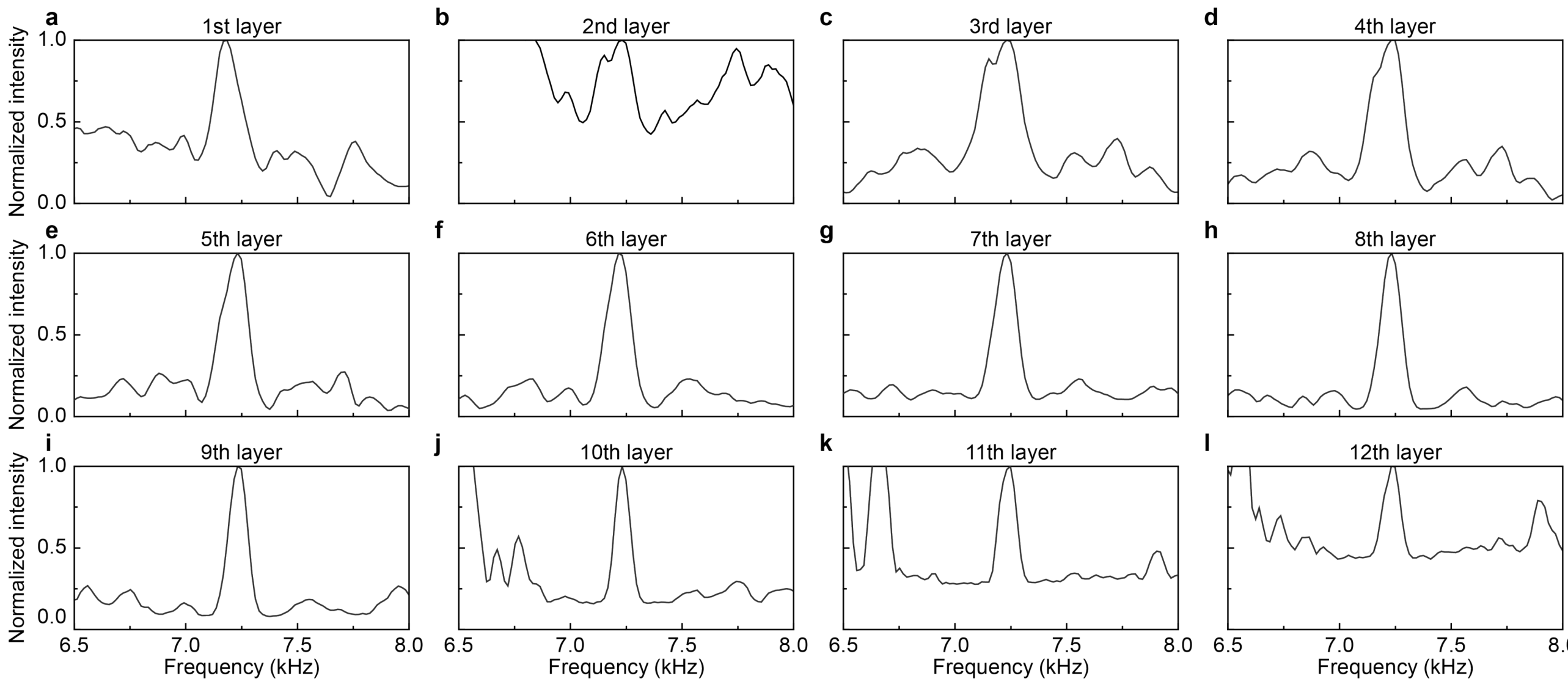

FIG. S15. | Measured response spectra in the 3D PC. a-l, Measured response spectra at the boundary resonator (belonging to sublattice Λ_a) that centers at $\phi_r = 0.873\pi$ for different layers.

The response spectra exhibit an intensity peak centered at 7.23 kHz. Here all the spectra are normalized by the intensity of its maxima.

In the main text, we present the acoustic field distributions of six representative layers in Figs. 3d-i. In Figs. S16a-f, we show the acoustic field distributions for remaining layers at 7.23 kHz, numbered 3, 4, 6, 8, 10, and 11 of the sample, which correspond to $\alpha_0 = \pi/3$, $\pi/2$, $5\pi/6$, $7\pi/6$, $3\pi/2$, and $5\pi/3$, respectively. These results provide a complete complement to those in the main text, and collectively offer a clear demonstration of the adiabatic evolution of the BMLZM.

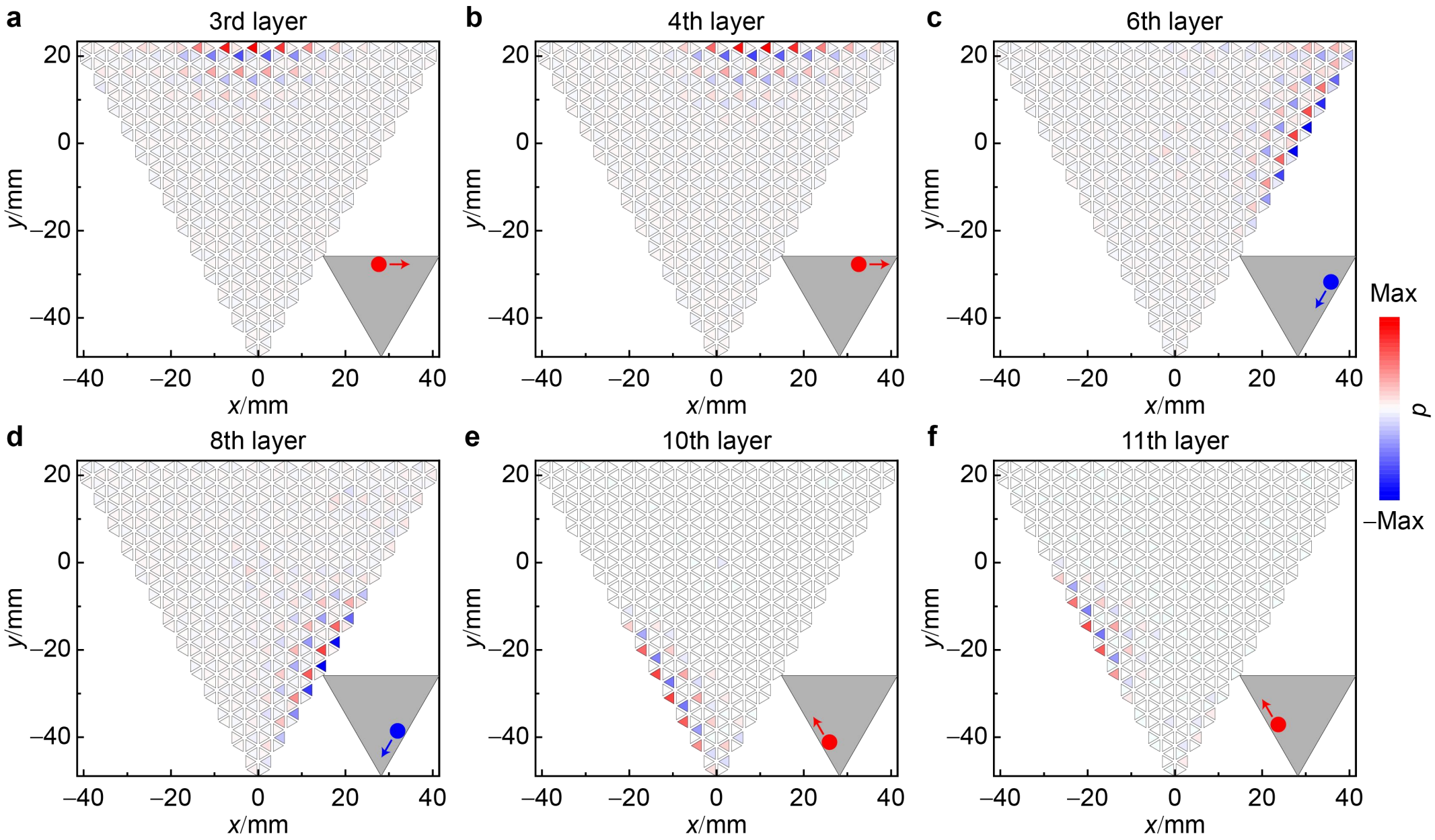

FIG. S16. | Measured field distributions in the 3D PC showing the evolution of the BMLZM. **a-f**, Measured field distributions of the BMLZMs on layers 3, 4, 6, 8, 10, and 11 at frequency of 7.23 kHz, corresponding to $\alpha_0 = \pi/3$, $\pi/2$, $5\pi/6$, $7\pi/6$, $3\pi/2$, and $5\pi/3$, respectively. The right insets depict the positions of the BMLZMs, with the color arrows indicating the directions of evolution as the layer number increases.

S-VIII. Braiding rule of multiple MLZMs

In the main text, we demonstrate the braiding of two BMLZMs that are initially located at the same boundary in a 3D PC. Here we elaborate the details of the other three typical configurations in triangular-shaped systems terminated by Boundary II (defined in Sec. II) with $N_b = 24$.

Firstly, for another two-BMLZMs braiding scenario depicted in the upper panel of Fig. S17a, we initial the two BMLZMs in the same phase at the beginning of the braiding process, denoted as $|\psi_0> = [1,1]^{\mathrm{T}}$. This initialization is depicted in the field distribution and sketch maps at $t = 0$ in the bottom panel of Fig. S17a. The modulation phase is given by $\phi_r = 2\theta_r + \alpha_0$. After adiabatically interchanging their position by increasing the modulation phase $\phi_r \rightarrow \phi_r + 2\pi$, one BMLZM get an additional π phase while the other BMLZM get no additional phase. Thus, the resulting states of the two BMLZMs after the braiding is denoted as $|\psi_T> = [-1,1]^{\mathrm{T}}$. This braiding process is also given by $|\psi_{\mathrm{T}}> = U_2|\psi_0>$ with $U_2 = [0,1;-1,0]$. Both BMLZMs change their phase after another period of braiding.

Next, we discuss the braiding process involving three BMLZMs considering two scenarios. In the first case, we design the distribution of modulation phase ϕ_r so that three BMLZMs are located at the same boundary as shown in the upper panel of Fig. S17b. The three BMLZMs are initialized in the same phase. The modulation phase distribution is

$$\phi_r = \begin{cases} \theta_r + \alpha_0, & 0 \leq \theta_r < 0.2\pi \\ 0.2\pi + 8.5(\theta_r - 0.2\pi) + \alpha_0, & 0.2\pi \leq \theta_r < 0.79\pi \\ 5.215\pi + 0.65(\theta_r - 0.79\pi) + \alpha_0, & 0.79\pi \leq \theta_r < 2\pi \end{cases} \quad \text{(S15)}$$

During the adiabatic evolution that $\phi_r \rightarrow \phi_r + 2\pi$, the rightmost BMLZM clockwise goes around the edge boundary and goes through three corners of the lattice, thus acquiring a π phase at the end of the braiding. Meanwhile, the remaining two BMLZMs move to the location of the next BMLZM without any phase change, as illustrated in the bottom panel of Fig. S17b. This process can be described by $|\psi_T> = [-1, \quad 1, \quad 1]^{\mathrm{T}} = U_3|\psi_0> = [0, 0, -1; 1, 0, 0; 0,1, 0][1, \quad 1, \quad 1]^{\mathrm{T}}$. In the second case shown in Fig. S17c, we set the initial distribution of ϕ_r obeys $\phi_r = 3\theta_r + \alpha_0$ so that three BMLZMs are located at three different boundaries. In the process of adiabatically evolving $\phi_r \rightarrow \phi_r + 2\pi$, all the BMLZMs move clockwise and relocate to the position of the next BMLZM. Since these BMLZMs all acquire an additional π phase as they cross the corners of the lattice, the braiding process can be depictive by the matrix $U_3^{'} = [0, 0, -1; -1,0,0; 0, -1,0]$.

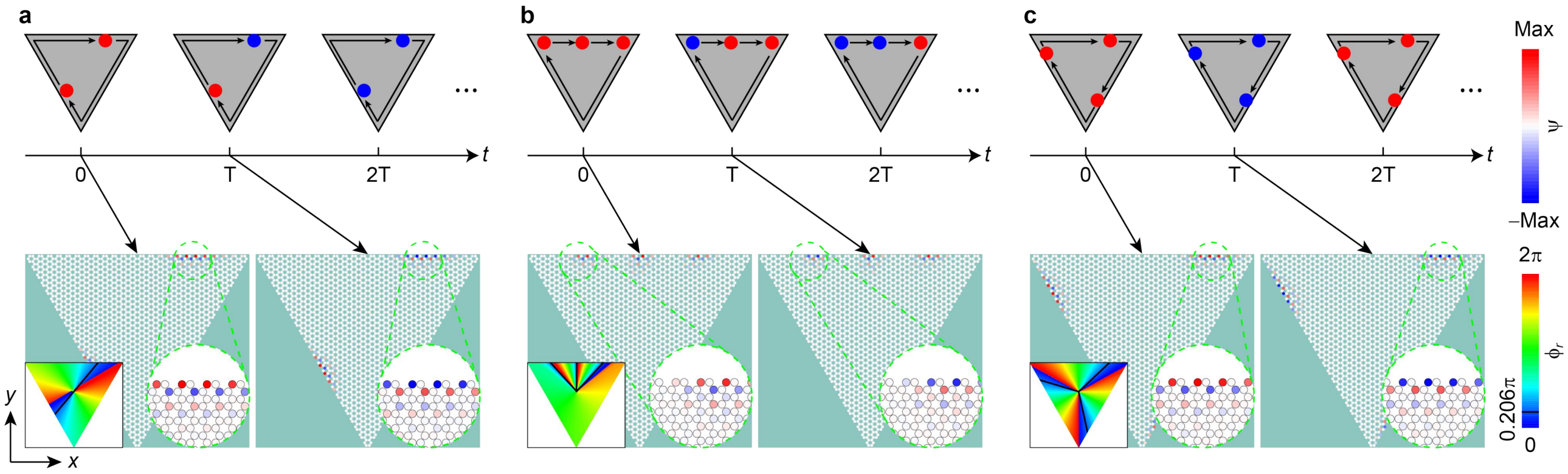

FIG. S17. | Braiding rule of multiple MLZMs in a triangular-shaped system. a, Braiding of two BMLZMs that are initially located at two different boundaries. Here T represents the period for the BMLZMs to rotate back to the initial locations (phase is not required) when the modulation phase $\phi_r \to \phi_r + 2\pi$. The red and blue dots in the upper panel sketch the locations of the BMLZMs with the color indicating whether there is a π phase change during this braiding process. The lower panels show the field distributions at $t = 0$ and $t = T$. If the color of a dot changes, then there is π phase obtained; otherwise, there is no additional phase. **b**, Braiding of three BMLZMs that are initially located at the same boundary. **c**, Braiding of three BMLZMs that are initially located at three different boundaries. The left insets show the distributions of the modulation phase $\phi_{\boldsymbol{r}}$, and the black lines mark the position with $\phi_{\boldsymbol{r}} = 0.206\pi$ (where the BMLZMs are localized at). Here the sample is terminated by Boundary II (defined in Sec. II) with $N_b = 24$.

S-IX. Two BMLZMs in one PC

The generation of multiple BMLZMs can be easily achieved by adjusting the distribution of ϕ_r in the lattice. We now design a PC to support two BMLZMs simultaneously. As shown in Fig. S18a, we give the schematic of a unit cell with key geometric parameters marked. The triangular prism acoustic resonators are form a graphene lattice with side lengths of $a_2 = 2.63$ cm. The height and side lengths of resonators (cyan and magenta belonging to the Λ_a and Λ_b sublattices, respectively) are $h_2 = 1.82$ cm and $b_2 = 1.58$ cm, respectively. The distance of resonators is $l_2 = 0.61$ cm. The widths $w_{\boldsymbol{r},i}$ of the coupling tubes are modulated according to the expression $w_{\boldsymbol{r},i} = (0.59 + \delta w_{\boldsymbol{r},i})$ cm, where $\delta w_{\boldsymbol{r},i} = \Delta_w e^{i(\boldsymbol{K}_+ \cdot \boldsymbol{s}_i + 2\boldsymbol{K}_+ \cdot \boldsymbol{r} + \phi_{\boldsymbol{r}})}/3 + \text{c.c.}$, with $\Delta_w = 0.23$. The height of all the

coupling tubes is kept the same at $s_2 = 0.65$ cm. The modulation phase distribution is designed as

$$\phi_r = \begin{cases} 1.33\theta_r - 0.13\pi + \alpha_0, & 0 \le \theta_r < 0.25\pi \\ 4\theta_r - 0.8\pi + \alpha_0, & 0.25\pi \le \theta_r < 0.75\pi \\ 1.33\theta_r + 1.2\pi + \alpha_0, & 0.75\pi \le \theta < 2\pi \end{cases} \quad \text{(S14)}$$

We also calculate the eigenfrequency spectrum of the PC with $N_b = 18$ terminated by Boundary II for different values of α_0, as shown in Fig. S18b. Four 'zero-energy' modes emerged within the bulk band gap, in which the cyan hollow circles and solid dots are doubly degenerated CMLZMs (i.e., CMLZM1 and CMLZM2; ~frequency of 9.95 kHz) and BMLZMs (i.e., BMLZM1 and BMLZM2; ~frequency of 9.88 kHz), respectively. The simulated eigenfield distributions of two BMLZMs is shown in Figs. S18c-h. The middle insets show the corresponding distributions of the modulation phase ϕ_r, where the black lines mark the position with $\phi_r = 0.206\pi$, matching well with the positions of the BMLZM on the Boundary II (defined in Sec. II). As α_0 increases from 0, the two BMLZMs rotate clockwise simultaneously. The original left BMLZM tardily relocates to the right side without any phase change. However, the original right BMLZM shifts rapidly along the sample boundary, and acquires an additional π phase when crossing each of the lattice corner.

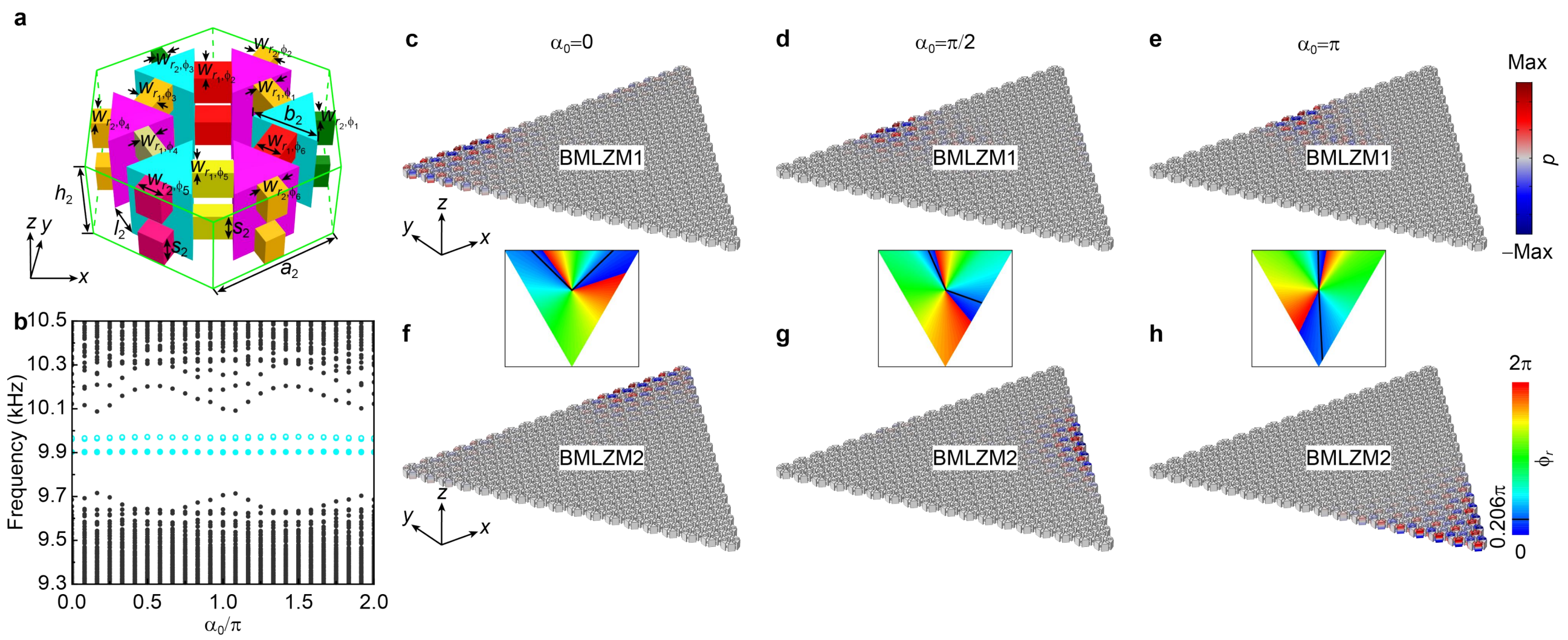

FIG. S18. | Two BMLZMs supported by one PC. a, Schematic of a unit cell with key geometric parameters defined: $a_2 = 2.63$ cm, $b_2 = 1.58$ cm, $h_2 = 1.82$ cm, $l_2 = 0.61$ cm, and $s_2 = 0.65$ cm. The widths $w_{r,i}$ of the coupling tubes are modulated according to the expression $w_{r,i} = (0.59 + \delta w_{r,i})$ cm, where $\delta w_{r,i} = \Delta_w e^{i(\boldsymbol{K}_+ \cdot \boldsymbol{s}_i + 2\boldsymbol{K}_+ \cdot \boldsymbol{r} + \phi_r)}/3 + \text{c.c.}$, with $\Delta_w = 0.23$. **b,** Eigenfrequency spectra of the triangular-shaped PC for different values of α_0. The cyan hollow circles and solid dots are doubly degenerated CMLZMs (i.e., CMLZM1 and CMLZM2; ~frequency

of 9.95 kHz) and BMLZMs (i.e., BMLZM1 and BMLZM2; ~frequency of 9.88 kHz), respectively, while the black dots represent the bulk states. **c-e**, Simulated eigenfield distributions of the BMLZM1 for $\alpha_0 = 0$ (**c**), $\pi/2$ (**d**), and π (**e**). **f-h**, Simulated eigenfield distributions of the BMLZM2 for $\alpha_0 = 0$ (**f**), $\pi/2$ (**g**), and π (**h**). The middle insets show the corresponding distributions of the modulation phase ϕ_r, and the black lines mark the position with $\phi_r = 0.206\pi$. $N_b = 18$ is kept in **b-h**.

The schematic of the braiding of two BMLZMs around another two CMLZMs is illustrated in Fig. S19a. Initially, two BMLZMs with same phase, denoted as $|\psi_0>=[1,1]^{\mathrm{T}}$, are located on the same boundary. After adiabatically increasing the modulation phase $\phi_r \rightarrow \phi_r + 2\pi$, the two BMLZMs rotate clockwise simultaneously. The original left BMLZM relocates to the initial position of the right BMLZM without any phase change, while the right BMLZM traverses the sample boundary and returns to the original position of the left BMLZM, acquiring an additional π phase. The resulting states of the two BMLZMs after the braiding are denoted as $|\psi_T>=[-1,1]^{\mathrm{T}}$, thus this braiding process is represented by $|\psi_{\mathrm{T}}>=U_2|\psi_0>$ with $U_2=[0,1;-1,0]$. Similar to Fig. 3 in the main text, we also treat the third spatial dimension (the z direction) as the synthetic time t. The actual 3D PC sample, with $N_b = 18$, is shown in Fig. S19b. It consists of 13 layers, fabricated using 3D printing technology. As the number of layers increases, the initial offset α_0 gradually increases from 0 to 2π in steps of $\pi/6$. Figure S19c shows a schematic of a unit cell with z-direction lattice constant $H_2 = 2.62$ cm. The geometry of the layered structure remains the same as before, but six gray cylindrical tubes with diameter $d_2 = 0.25$ cm are introduced to provide weak coupling along the z direction.

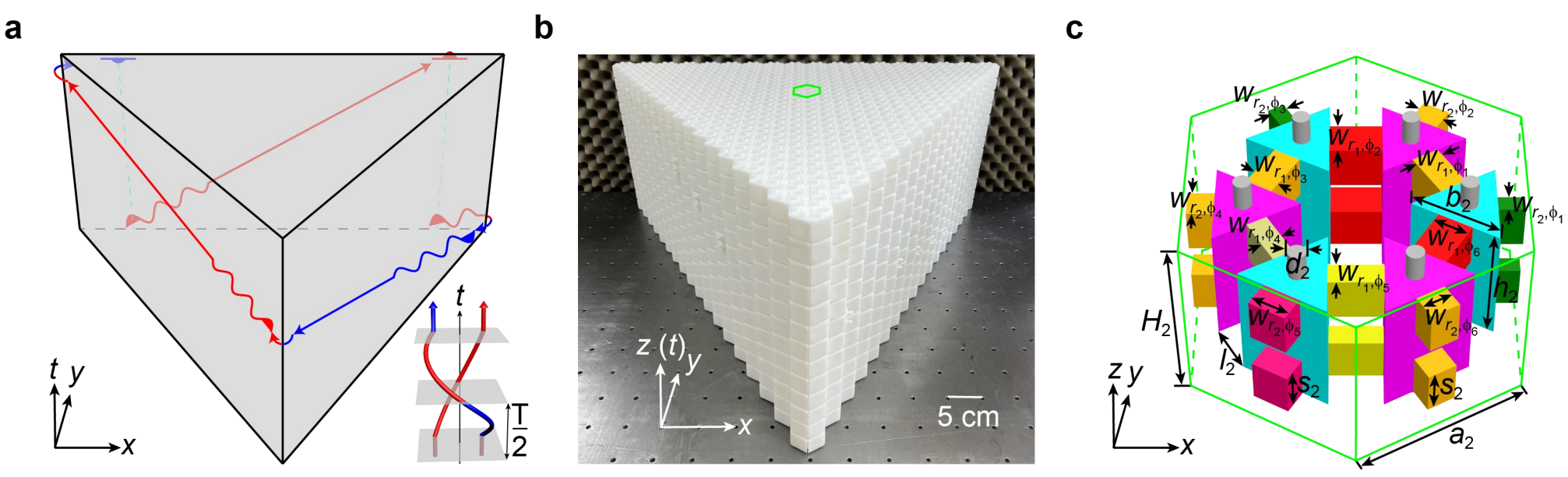

Fig. S19. | Braiding of two BMLZMs in a 3D PC. a, Braiding trajectory of the two BMLZMs in a

triangular-prism space (x-y-t). The inset illustrates the schematic of the braiding process, with T representing the period for the BMLZMs to rotate back to the initial configuration when the modulation phase $\phi_r \rightarrow \phi_r + 2\pi$. **b**, Photograph of a triangular-prism PC sample with $N_b = 18$ for the braiding of two BMLZMs. **c**, Schematic of a unit cell with key geometric parameters marked. The prism-shaped resonant cavity in cyan (magenta) emulates the sublattice Λ_a (Λ_b). All the other unspecified parameters are kept the same as Fig. S18.

To obtain the frequency of the two BMLZMs in the real 3D PC above, we measure the response spectra at $\phi_r = 0.206\pi$ for all the layers. Two dipolar sound sources (two loudspeakers with opposite phase) are positioned at the boundary of the seven layer to excite the BMLZMs. As shown in Figs. S20a-m, we present the measured response spectra in the 3D PC for whole layers. These response spectra exhibit an intensity peak centered at $9.96\ \mathrm{kHz}$, slightly higher than the $9.88\ \mathrm{kHz}$ simulated in the discrete PCs in Fig. S18. The response spectra are normalized by the peak intensity of BMLZM1. Similarly, the frequency of the two BMLZMs experiences a negligible shift due to introduction of the weak interlayer coupling.

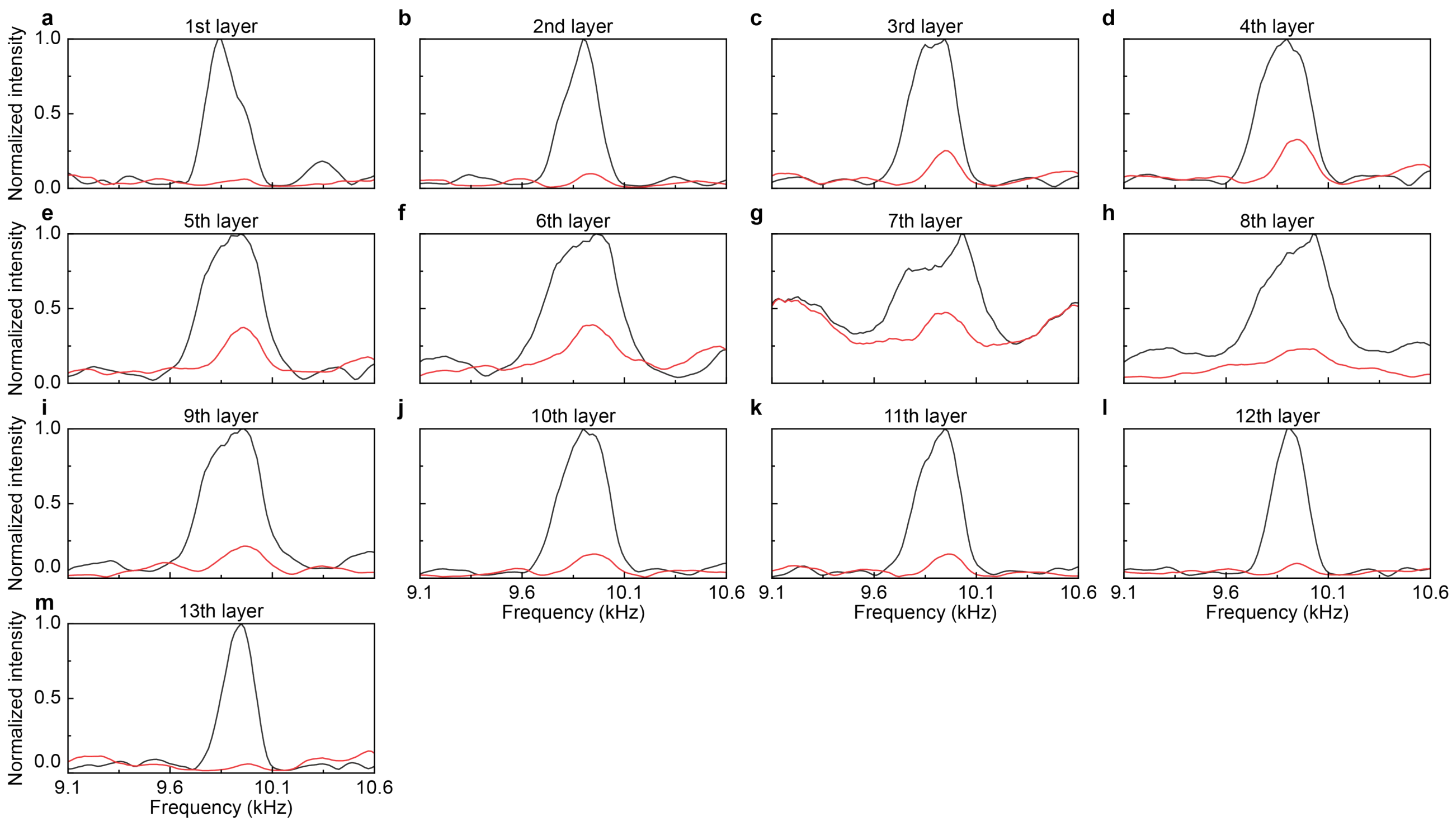

FIG. S20. | Measured response spectra in the 3D PC supporting the braiding of two BMLZMs.

a-m, Measured response spectra at the boundary resonators (belonging to sublattice Λ_a) that centers at $\phi_r = 0.206\pi$ for different layers. The black and red lines denote the spectra measured at the BMLZM1 and BMLZM2, respectively. The response spectra exhibit an intensity peak centered at 9.96 kHz, and are normalized by the peak intensity of the BMLZM1.

In the main text, we present the acoustic field distributions of six representative layers in Figs. 4a-f. In Figs. S21a-g, we show the acoustic field distributions for remaining layers at 9.96 kHz, numbered 2, 3, 5, 6, 8, 10, and 12 of the sample, which correspond to $\alpha_0 = \pi/6$, $\pi/3$, $2\pi/3$, $5\pi/6$, $7\pi/6$, $5\pi/3$, and $11\pi/6$, respectively. These results provide a complete complement to those in the main text, and collectively offer a clear demonstration of the braiding of two BMLZMs.

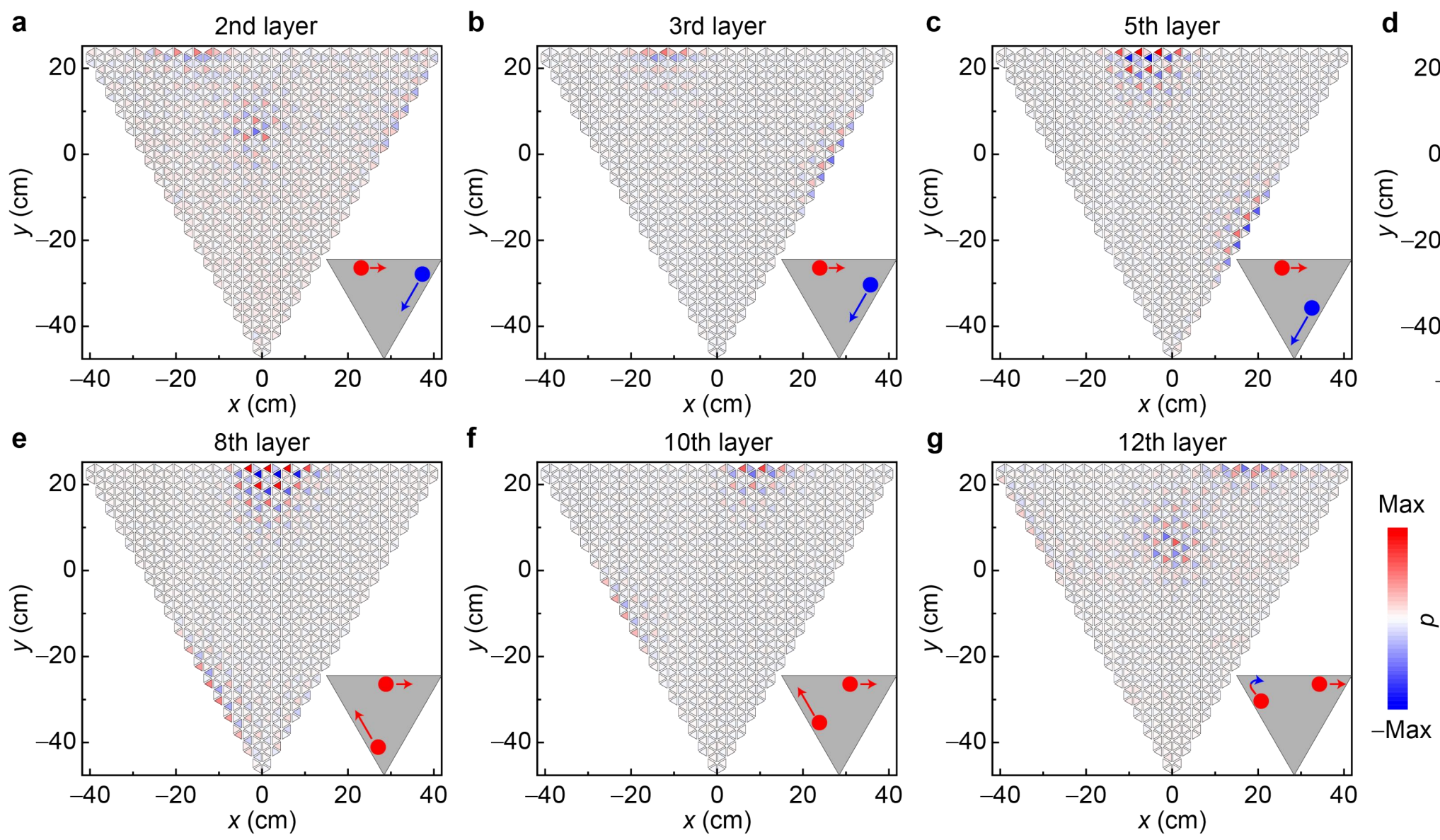

FIG. S21. | Measured field distributions in the 3D PC supporting the braiding of two BMLZMs. a-g, Measured field distributions of the two BMLZMs on the remaining layers at an operating frequency of 9.96 kHz, numbered 2, 3, 5, 6, 8, 10, and 12 of the sample, corresponding to $\alpha_0 = \pi/6$, $\pi/3$, $2\pi/3$, $5\pi/6$, $7\pi/6$, $5\pi/3$, and $11\pi/6$, respectively. The lower-right insets depict the positions of the two BMLZMs, with the colored arrows indicating the directions of evolution as the layer number increases.

S-X. Braiding of multiple BMLZMs in one PC

Here, we design a PC to simulate the braiding of four BMLZMs. The PC is chosen to have a triangular shape, and only type-II boundaries, as defined in Sec. II, are retained for simplicity. We set the initial phase distribution as $\phi_r = 4\theta_r + \alpha_0$, such that four BMLZMs are supported in total. The black lines in the lower insets of Figs. S22a1–d1 mark the positions where $\phi_r = 0.206\pi$, which agree well with the locations of the BMLZMs on Boundary II. The four BMLZMs are initialized with the same phase, as shown by the eigenfield distributions in Figs. S22a2–a5. Among them, two BMLZMs are located at the upper boundary, while the other two are located at the remaining two side boundaries. For clarity, we also summarize the location and phase of each BMLZM in the right panel of Fig. S22a1. Here, all four dots are red, indicating that the four BMLZMs have the same phase.

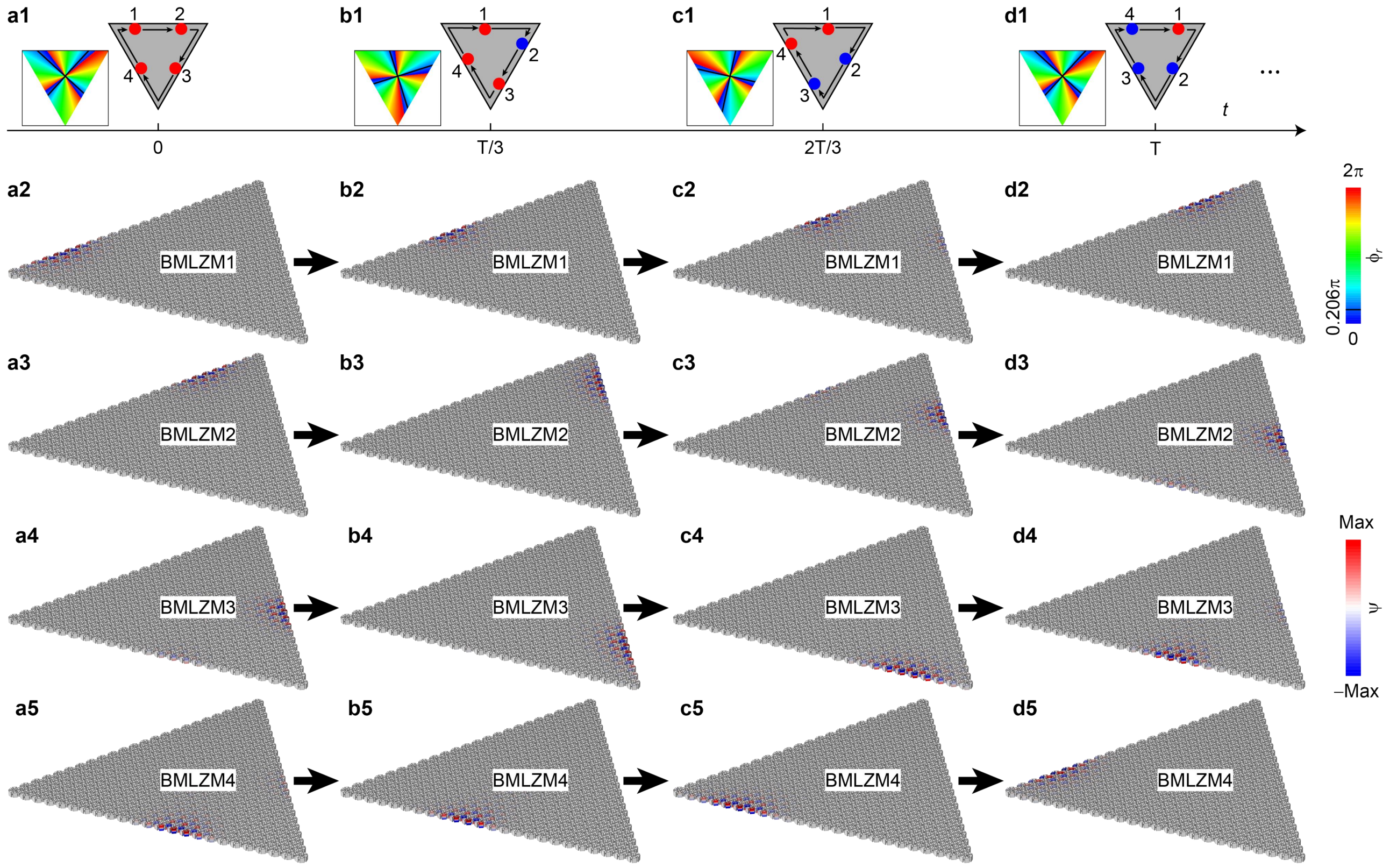

FIG. S22. | Braiding of four BMLZMs. a1-d1, The locations of the BMLZMs (right panels) and the corresponding distributions of the modulation phase ϕ_r (left panels). Here, red and blue dots indicate whether there is a π phase change during this braiding process. Black lines in modulation phase distributions mark the position with $\phi_r = 0.206\pi$. T represents the period for the BMLZMs to rotate back to the initial locations of next BMLZM when the modulation phase $\phi_r \to \phi_r + 2\pi$. **a2-a5,** Simulated eigenfield distributions of the four BMLZMs at initial locations. Same as **a2-a5**,

b2-b5 (**c2-c5** and **d2-d5**) show the corresponding eigenfield distributions at $T/3$ ($2T/3$ and T). $N_b = 24$ is kept in **a-d** and other unspecified parameters are the same as those in Fig. 2.

As the evolution parameter varies within one period, the four BMLZMs rotate clockwise simultaneously. As illustrated in Figs. S22a1–d1, during the adiabatic evolution $\phi_r \to \phi_r + 2\pi$, each BMLZM is relocated to the position of the next BMLZM. Specifically, BMLZM1 moves to the original position of BMLZM2 without acquiring an additional phase. In contrast, the original BMLZM2, BMLZM3, and BMLZM4 move along the sample boundary to the original positions of BMLZM3, BMLZM4, and BMLZM1, respectively, and each acquires an additional π phase (the color of corresponding dots changes from red to blue). Therefore, according to the definition in Sec. VIII, this braiding process can be described by a braiding matrix with $U_4 = [0,\ 0, 0,1; -1, 0,0, 0; 0, -1, 0,0; 0,0, -1, 0]$.

References

1. Hou, C. Y., Chamon, C., & Mudry, C. Electron fractionalization in two-dimensional graphenelike structures. *Phys. Rev. Lett.* **98**, 186809 (2007).
2. Wu, L. H. & Hu, X. Scheme for achieving a topological photonic crystal by using dielectric material. *Phys. Rev. Lett.* **114**, 223901 (2015).
3. Noh, J. et al., Topological protection of photonic mid-gap defect modes. *Nat. Photon.* **12**, 408 (2018).
4. Jackiw, R. & Rebbi, C. Solitons with fermion number ½. *Phys. Rev. D* 13, 3398 (1976).
5. Noh, J. et al. Braiding photonic topological zero modes. *Nat. Phys.* **16**, 989-993 (2020).
6. Li, T. et al., Acoustic Möbius insulators from projective symmetry. *Phys. Rev. Lett.* **128**, 116803 (2022).
7. Ma, Q. et al., Observation of higher-order nodal-line semimetal in phononic crystals. *Phys. Rev. Lett.* **132**, 066601 (2024).
