# Supplementary material for: Direct observation of braiding of Majorana-like zero modes in real space
Source: Nat Commun. 2026 Jul 31;17:9279. doi: 10.1038/s41467-026-75765-4 (PMC13527056; doi:10.1038/s41467-026-75765-4)
Supplement: Supplementary file 2 [file 41467_2026_75765_MOESM2_ESM.pdf]

nature portfolio

# Peer Review File

# Direct observation of braiding of Majorana-like zero modes in real space

Corresponding Author: Professor Zhengyou Liu

**This file contains all reviewer reports in order by version, followed by all author rebuttals in order by version.**

Version 0:

Reviewer comments:

Reviewer #1

(Remarks to the Author)
In this paper, the authors utilize Majorana-like zero modes (MLZMs) in a non-interacting Hamiltonian to realize quasiparticle excitations and braiding of Majorana zero modes, which are typically thought to exist in p-wave superconducting systems. Interestingly, they find that MLZMs are present not only at the vortex core but also at the armchair boundary, enabling boundary-bound Majorana-like zero modes (BMLZMs) to circle around the vortex-center MLZM, thereby achieving the braiding of two MLZMs. I believe this article demonstrates the braiding of elusive Majorana zero modes found in quantum materials using a highly controllable classical system. I recommend this article for publication in Nature Communications. Before that, however, I would like the authors to address the following questions.
1. In the main text, the authors show that nontrivial BMLZMs can be obtained by using armchair boundaries. In Supplementary Material Note I, they consider the case of zigzag boundaries and find: (1) For zigzag boundaries, a uniform Kekulé texture leads to trivial boundary states, and a vortex Kekulé texture does not produce nontrivial BMLZMs; (2) For armchair boundaries, a uniform Kekulé texture does not result in trivial boundary states, while a vortex Kekulé texture gives rise to nontrivial BMLZMs. Please provide a physical explanation for this phenomenon.
2. This work stops at demonstrating the braiding of two BMLZMs. But braiding is more interesting when three or more modes are involved. Can this be done in the system here, e.g., maybe a hexagonal-shaped lattice can support more boundary modes to be braided? If so, it will be great to at least provide some simulation results. If not, what are the limitations? Are there alternative ways to increase the number of braided BMLZMs?
3. Are the boundary states in Fig. 1(d-h) appearing at the phase transition point ϕ_r=0.873 because the boundary bands have different winding numbers on either side of the transition? If so, since the vacuum outside the system boundary should also have a different winding number from the boundary band, why do boundary states appear only at the domain walls between two interfaces along the upper boundary in Fig. S5e, and not along the entire upper boundary? Also, will there be boundary states at the gaps at 0 and π of the bands in Fig. S5c?
4. When two MLZMs annihilation, they should annihilate into the vacuum state and the fermion-occupied state. These two states have energies of $\pm\epsilon$, with a gap of $2\epsilon$. Moreover, the vacuum state and the fermion-occupied state should have identical spatial distributions. To verify this, could the authors plot the spatial distributions of the vacuum and fermion-occupied states formed after the annihilation of the two MLZMs, i.e., the two states with energies $\pm\epsilon$?
5. The paper did not provide sufficient background in the introduction. There have been significant dveleopments on braiding classical setting in the past few years, e.g., Nat. Photon. 16, 390–395 (2022); Nat. Phys. 18, 179–184 (2022); Nat. Commun. 16, 3650 (2025). There are also those that make use of the properties of exceptional points in non-Hermitian systems, please also survey those briefly.

Reviewer #2

(Remarks to the Author)
I co-reviewed this manuscript with one of the reviewers who provided the listed reports. This is part of the Nature Communications initiative to facilitate training in peer review and to provide appropriate recognition for Early Career Researchers who co-review manuscripts.

Reviewer #3

(Remarks to the Author)

In the article entitled “Direct observation of braiding of Majorana-like zero modes in real space”, Ma et al. demonstrated the real-space braiding of Majorana-like zero modes (MLZMs) bounded on boundaries. By modulating the Kekulé modulation phase layer by layer, the MLZMs adiabatically evolve on the surfaces of a three-dimensional phononic crystal. The phase accumulation of MLZMs is captured layer by layer, signifying the braiding process upon exchanging the positions of MLZMs.

Instead of relying on interference between core-localized MLZMs, this work not only offers convenient tuning of MLZM position but also provides a direct real-space visualization of braiding process. Overall, the manuscript is written clearly and logically. I would recommend the publication on Nature Communications if the authors could address the following concerns:

1. In the introduction (line 55), the authors claimed the observation of “characteristic phase accumulation that signifies non-Abelian braiding”. However, it is unclear from the results whether non-Abelian characteristics are direct consequences of phase accumulation, especially when only two MLZMs are involved. Did the authors mean the braiding between more MLZMs, i.e., the cases that show the non-Abelian nature [Phys. Rev. Lett. 117, 073901 (2016)]?

2. In Fig. 3, is the single boundary-bound MLZM (BMLZM) already braided with vortex-center-bound MLZM (CMLZM)? It will be helpful to track the evolution of CMLZM, compared to the sole CMLZM in Ref. [Nat. Phys. 16, 989 (2020)].

3. In Fig. 4, the intensity profile of BMLZM2 looks much weaker than BMLZM1, making its localization hard to see from the experimental result. Its frequency spectrum also shows unclear peak at the zero-mode frequency (Fig. S17). Could the authors explain why?

4. About the Jackiw-Rebbi formalism for BMLZM. Which symmetry protects the quantized Zak phase of topological boundary bands? Such topological transition of boundary bands can be related to embedded topology [Phys. Rev. B 100, 115126 (2019); Phys. Rev. Lett. 135, 136602 (2025)].

5. Continuing from point 4, the authors also directly relate the Jackiw-Rebbi formalism to the phononic crystal. To be clear, could the authors verify whether the phononic crystal hosts the topological boundary bands?

6. For a thorough survey on MLZM applications, the authors may consider citing its recent lasing realizations in photonics [Nat. Commun. 14, 707 (2023); Light Sci. Appl. 12, 255 (2023)].

I also suggest the authors take note of these comments for smoother reading:

1. In Figs. 2-3, are the units of x, y axes in the field mapping consistent with the experimental setup?

2. To differentiate between Figs. 3-4, it will be helpful to include the Kekulé phase map and braiding configuration of BMLZMs in Fig. 4, where multiple zero modes are involved.

3. In the analytical solution of BMLZM [Eq. (S7)], what do $x_0$ and $y_0$ represent?

Version 1:

Reviewer comments:

Reviewer #1

(Remarks to the Author)
The authors’ replies have adequately addressed all my concerns. I recommend the publication of the current version in Nature Communications.

Reviewer #2

(Remarks to the Author)
I co-reviewed this manuscript with one of the reviewers who provided the listed reports. This is part of the Nature Communications initiative to facilitate training in peer review and to provide appropriate recognition for Early Career Researchers who co-review manuscripts.

Reviewer #3

(Remarks to the Author)
The authors have answered my questions satisfactorily. I recommend the publication of this work.

**Open Access** This Peer Review File is licensed under a Creative Commons Attribution 4.0 International License, which permits use, sharing, adaptation, distribution and reproduction in any medium or format, as long as you give appropriate credit to the original author(s) and the source, provide a link to the Creative Commons license, and indicate if changes were made.
In cases where reviewers are anonymous, credit should be given to 'Anonymous Referee' and the source.
The images or other third party material in this Peer Review File are included in the article's Creative Commons license, unless indicated otherwise in a credit line to the material. If material is not included in the article's Creative Commons license and your intended use is not permitted by statutory regulation or exceeds the permitted use, you will need to obtain permission directly from the copyright holder.
To view a copy of this license, visit https://creativecommons.org/licenses/by/4.0/

**Reviewer #1 (Remarks to the Author):**

In this paper, the authors utilize Majorana-like zero modes (MLZMs) in a non-interacting Hamiltonian to realize quasiparticle excitations and braiding of Majorana zero modes, which are typically thought to exist in p-wave superconducting systems. Interestingly, they find that MLZMs are present not only at the vortex core but also at the armchair boundary, enabling boundary-bound Majorana-like zero modes (BMLZMs) to circle around the vortex-center MLZM, thereby achieving the braiding of two MLZMs. I believe this article demonstrates the braiding of elusive Majorana zero modes found in quantum materials using a highly controllable classical system. I recommend this article for publication in Nature Communications. Before that, however, I would like the authors to address the following questions.

**Reply:** We thank the referee for the positive evaluation of our work and the recommendation for its publication in Nature Communications. We address each comment in detail below.

1. In the main text, the authors show that nontrivial BMLZMs can be obtained by using armchair boundaries. In Supplementary Material Note I, they consider the case of zigzag boundaries and find: (1) For zigzag boundaries, a uniform Kekulé texture leads to trivial boundary states, and a vortex Kekulé texture does not produce nontrivial BMLZMs; (2) For armchair boundaries, a uniform Kekulé texture does not result in trivial boundary states, while a vortex Kekulé texture gives rise to nontrivial BMLZMs. Please provide a physical explanation for this phenomenon.

**Reply:** We thank the referee for this insightful comment. In Fig. S1 (also see Figs. R1a-c), we considered a uniform Kekulé texture modulation, for which the number of zero modes is eight. The eigenfield distributions of these eight trivial zero modes are only located at zigzag boundaries.

In Fig. S2 (also see Figs. R1d-f), we considered a vortex Kekulé texture modulation $\phi_r$ with the initial offset $\alpha_0 = 0.5$, for which the number of zero modes increases to ten. Here eight zero modes are inherited from the uniform Kekulé texture case and are localized at the zigzag boundaries. Therefore, each introduced vortex Kekulé texture

will increases the number of MLZMs by two, corresponding to one BMLZM and one CMLZM, consistent with Fig. 1 of the main text. As shown in Fig. R1f, the BMLZM appears on the upper boundary of the rectangular system. Figs. S1 and S2 together may have led to the referee's conclusion that a vortex Kekulé texture does not produce nontrivial BMLZMs at the zigzag boundary, but instead produces a nontrivial BMLZM at the armchair boundary.

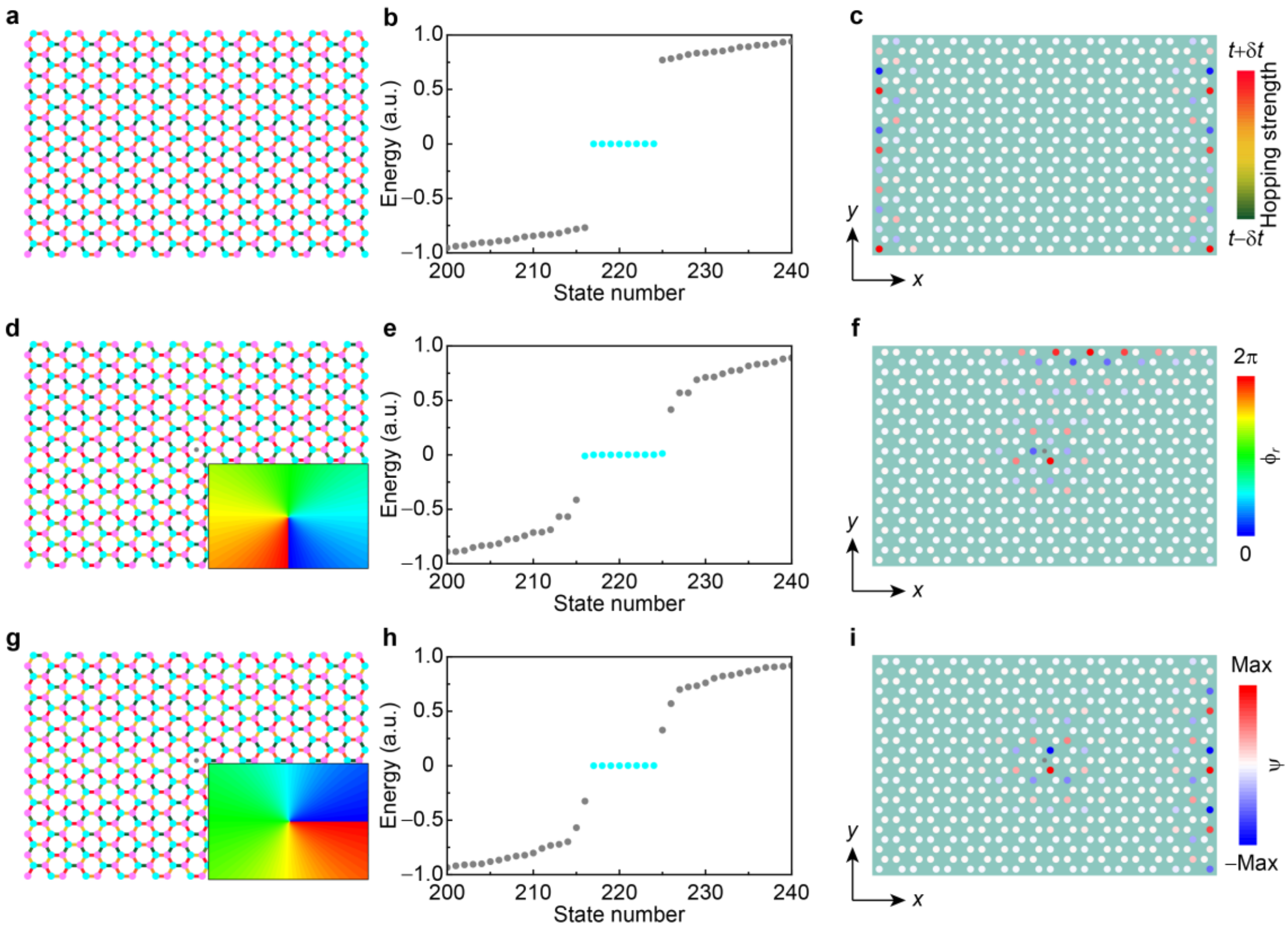


**FIG. R1. | Zero modes in a rectangular-shaped honeycomb lattice with a uniform-texture and a vortex-texture Kekulé modulations. a**, **d**, **g**, Tight-binding models for the rectangular-shaped honeycomb lattice with a uniform-texture Kekulé modulation (**a**), and vortex-texture Kekulé modulations with $\alpha_0 = 0.5$ (**d**) and $\alpha_0 = 0$ (**g**). The color of the bonds denotes the hopping strengths. The lower-right insets in **d** and **g** show the corresponding modulation phase distribution $\phi_r$. **b**, **e**, **h**, Eigenenergy spectra near zero energy for the tight-binding models shown in **a**, **d**, and **g**, respectively. Cyan points highlight the zero modes. **c**, **f**, **i**, Field distribution of one of the zero-energy modes. In **f** and **i**, the zero-energy mode shows the hybridization of the CMLZM with the BMLZM localized at the upper armchair boundary (**f**) and the right zigzag boundary (**i**),

respectively.

In fact, similar as shown in Fig. 1, the location of the BMLZM under this geometry is also determined by $\alpha_0$. If we vary $\alpha_0$, the nontrivial BMLZM can move onto the zigzag boundary. In Figs. R1g-i, we considered a vortex-texture Kekulé modulations with $\alpha_0 = 0$. Figure R1g shows the tight-binding model together with the distribution of the modulation phase, shown in the inset. Figures R1h and R1i show the corresponding eigenenergy spectrum and the eigenfield distribution of one of the coupled MLZMs, respectively. In this case, the BMLZM coupled to the CMLZM is localized at the zigzag boundary. We have also checked the field distributions of all zero-energy modes and found no zero mode localized at the armchair boundary. Therefore, the BMLZM can also appear at the zigzag boundary, depending on the value of $\alpha_0$. We also note that the number of zero-energy modes is now eight, instead of ten as in Fig. R1e. This is because, when the BMLZM moves to the zigzag boundary, it hybridizes with the zero-energy mode originally localized at the zigzag boundary. Upon hybridization, the two zero-energy modes are shifted away from zero energy, leaving only eight zero-energy modes. This feature is also consistent with Figs. S3 and S4.

To clarify this point, we have added the following two sentences before Fig. S2 in the revised Supplementary Information (SI) (lines 36-38), as "Comparing to the uniform-texture modulation in Fig. S1, two more additional zero mode appears, one at the vortex center (CMLZM) while another appears at the lattice boundary (BMLZM). Similar as Fig. 1, the location of the BMLZM is also determined by the initial offset $\alpha_0$."

2. This work stops at demonstrating the braiding of two BMLZMs. But braiding is more interesting when three or more modes are involved. Can this be done in the system here, e.g., maybe a hexagonal-shaped lattice can support more boundary modes to be braided? If so, it will be great to at least provide some simulation results. If not, what are the limitations? Are there alternative ways to increase the number of braided BMLZMs?

**Reply:** We thank the referee for this constructive suggestion. Indeed, braiding becomes

more interesting when multiple BMLZMs are involved. Figure S17 shows the braiding of two and three BMLZMs within the tight binding model. Following the referee's suggestion, we have added a new section to the Supplementary Information, Sec. X, where we simulate the braiding of multiple BMLZMs in a phononic crystal (PC). Specifically, we consider a triangular PC with armchair boundaries, similar to that in the main text, but with an initial phase distribution satisfying $\phi_r = 4\theta_r + \alpha_0$. This configuration supports four BMLZMs. We then gradually vary the modulation phase as $\phi_r \to \phi_r + 2\pi$ and record the corresponding mode distributions. The results are summarized in Figs. S22(a1–d1). We also note that braiding in a hexagonal-shaped lattice is more complex, because different types of armchair boundaries are involved, as shown in Fig. S10.

The simulation results and related discussions are provided in Supplementary as S-X (lines 569-597), also see below.

## S-X. Braiding of multiple BMLZMs in one PC

Here, we design a PC to simulate the braiding of four BMLZMs. The PC is chosen to have a triangular shape, and only type-II boundaries, as defined in Sec. II, are retained for simplicity. We set the initial phase distribution as $\phi_r = 4\theta_r + \alpha_0$, such that four BMLZMs are supported in total. The black lines in the lower insets of Figs. S22a1–d1 mark the positions where $\phi_r = 0.206\pi$, which agree well with the locations of the BMLZMs on type-II Boundary. The four BMLZMs are initialized with the same phase, as shown by the eigenfield distributions in Figs. S22a2–a5. Among them, two BMLZMs are located at the upper boundary, while the other two are located at the remaining two side boundaries. For clarity, we also summarize the location and phase of each BMLZM in the right panel of Fig. S22a1. Here, all four dots are red, indicating that the four BMLZMs have the same phase.

As the evolution parameter varies within one period, the four BMLZMs rotate clockwise simultaneously. As illustrated in Figs. S22a1–d1, during the adiabatic evolution $\phi_r \to \phi_r + 2\pi$, each BMLZM is relocated to the position of the next BMLZM. Specifically, BMLZM1 moves to the original position of BMLZM2 without

acquiring an additional phase. In contrast, the original BMLZM2, BMLZM3, and BMLZM4 move along the sample boundary to the original positions of BMLZM3, BMLZM4, and BMLZM1, respectively, and each acquires an additional $\pi$ phase (the color of corresponding dots changes from red to blue). Therefore, according to the definition in Sec. VIII, this braiding process can be described by a braiding matrix with $U_4 = [0,\ 0,0,1; -1,0,0,0; 0,-1,0,0; 0,0,-1,0]$.

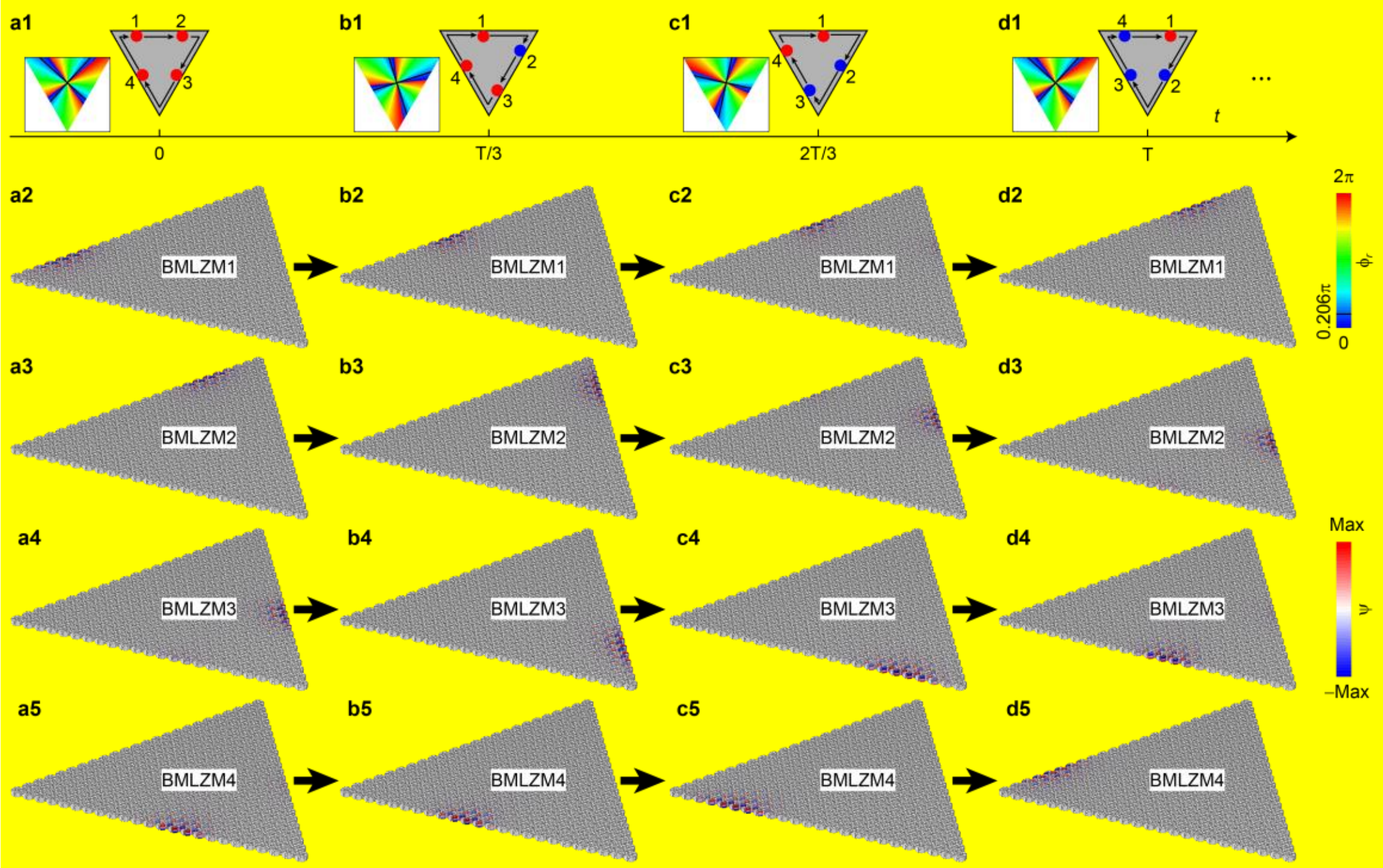


**FIG. S22. | Braiding of four BMLZMs. a1-d1,** The locations of the BMLZMs (right panels) and the corresponding distributions of the modulation phase $\phi_r$ (left panels). Here, red and blue dots indicate whether there is a $\pi$ phase change during this braiding process. Black lines in modulation phase distributions mark the position with $\phi_r = 0.206\pi$. $T$ represents the period for the BMLZMs to rotate back to the initial locations of next BMLZM when the modulation phase $\phi_r \rightarrow \phi_r + 2\pi$. **a2-a5,** Simulated eigenfield distributions of the four BMLZMs at initial locations. Same as **a2-a5**, **b2-b5** (**c2-c5** and **d2-d5**) show the corresponding eigenfield distributions at $T/3$ ($2T/3$ and $T$). $N_b = 24$ is kept in **a-d** and other unspecified parameters are the same as those in Fig. 2.

3. Are the boundary states in Fig. 1(d-h) appearing at the phase transition point

ϕ_r=0.873 because the boundary bands have different winding numbers on either side of the transition? If so, since the vacuum outside the system boundary should also have a different winding number from the boundary band, why do boundary states appear only at the domain walls between two interfaces along the upper boundary in Fig. S5e, and not along the entire upper boundary? Also, will there be boundary states at the gaps at 0 and π of the bands in Fig. S5c?

**Reply:** We thank the referee for the insightful question. As discussed in Part of Sec. S-II, we calculate the Zak phases of the boundary bands of the ribbon lattice terminated by Boundary I. Here there is a (0, 0) to ($\pi$, $\pi$) topological phase transition between the two boundary modes localized on the upper boundary (denoted by the red bands in Fig. S5 b-d). Thus, such a phase transition is similar to the well-known Jackiw-Rebbi mechanism. Focusing only on the boundary modes inside the bulk gap, the ribbon lattice in Fig. S5a can be effectively reduced to a one-dimensional system along the $x$ direction. The reason is that the boundary modes are confined in the $y$ direction by the vacuum on one side and the bulk lattice on the other side. In this effective one-dimensional system, the edge mode is found at the domain wall where the modulation phase crosses $\phi_r = 0.873\pi$. In other words, there is no boundary between the effective one-dimensional system along the $x$ direction and the vacuum on the upper side of the system.

In Fig. S5c, we show four boundary bands. Two of them, shown in red, have Zak phases of (0, 0), while the other two, shown in dark green, have Zak phases of (π, π). Since we consider a ribbon lattice, these boundary modes are localized at two different boundaries of the ribbon and are therefore spatially separated. Specifically, the two red boundary bands are localized at the upper boundary, whereas the two dark-green boundary bands are localized at the lower boundary. Therefore, there should be no edge state in the gaps between the red bands with Zak phase 0 and the dark-green bands with Zak phase π in Fig. S5c.

4. When two MLZMs annihilation, they should annihilate into the vacuum state and the fermion-occupied state. These two states have energies of ±ϵ, with a gap of 2ϵ.

Moreover, the vacuum state and the fermion-occupied state should have identical spatial distributions. To verify this, could the authors plot the spatial distributions of the vacuum and fermion-occupied states formed after the annihilation of the two MLZMs, i.e., the two states with energies $\pm\epsilon$?

**Reply:** We thank the referee for the constructive suggestion. The referee is correct that, when the two MLZMs are brought sufficiently close together, they can annihilate each other, yielding two eigenstates with energies $\pm\varepsilon$.

To demonstrate this feature, we plot the spatial field distributions of the two modes as a function of the distance $d_1$ between the vortex centers. The relevant results and discussions are provided in part f of SI S-II (lines 247-265), also see below.

**f. Annihilation of the two CMLZMs**

This section considers the annihilation of two CMLZMs with opposite charges. We start from two CMLZMs whose vortex centers are separated by a distance $d_1$ larger than twice the effective size of a single CMLZM, and then gradually decrease this distance. In Fig. S8a, we first show the eigenenergy spectrum as a function of $d_1$. When $d_1 = 6\sqrt{3}a$, the two CMLZMs are far apart, with clearly separated spatial field distributions, as shown in Fig. S8b, and their eigenenergies are nearly degenerate.

As the vortex centers, and hence the CMLZMs, move closer to each other, their mutual coupling increases and the eigenenergies split into $\pm\varepsilon$, as shown in Figs. S8c, d. The corresponding eigenstates can be interpreted as even and odd combinations of the two original CMLZMs. When the vortex centers become very close, for example at $d_1/\sqrt{3}a = 0.01$ in Fig. S8e, the eigenfield gradually loses the characteristic localization of the CMLZMs and becomes spatially extended.

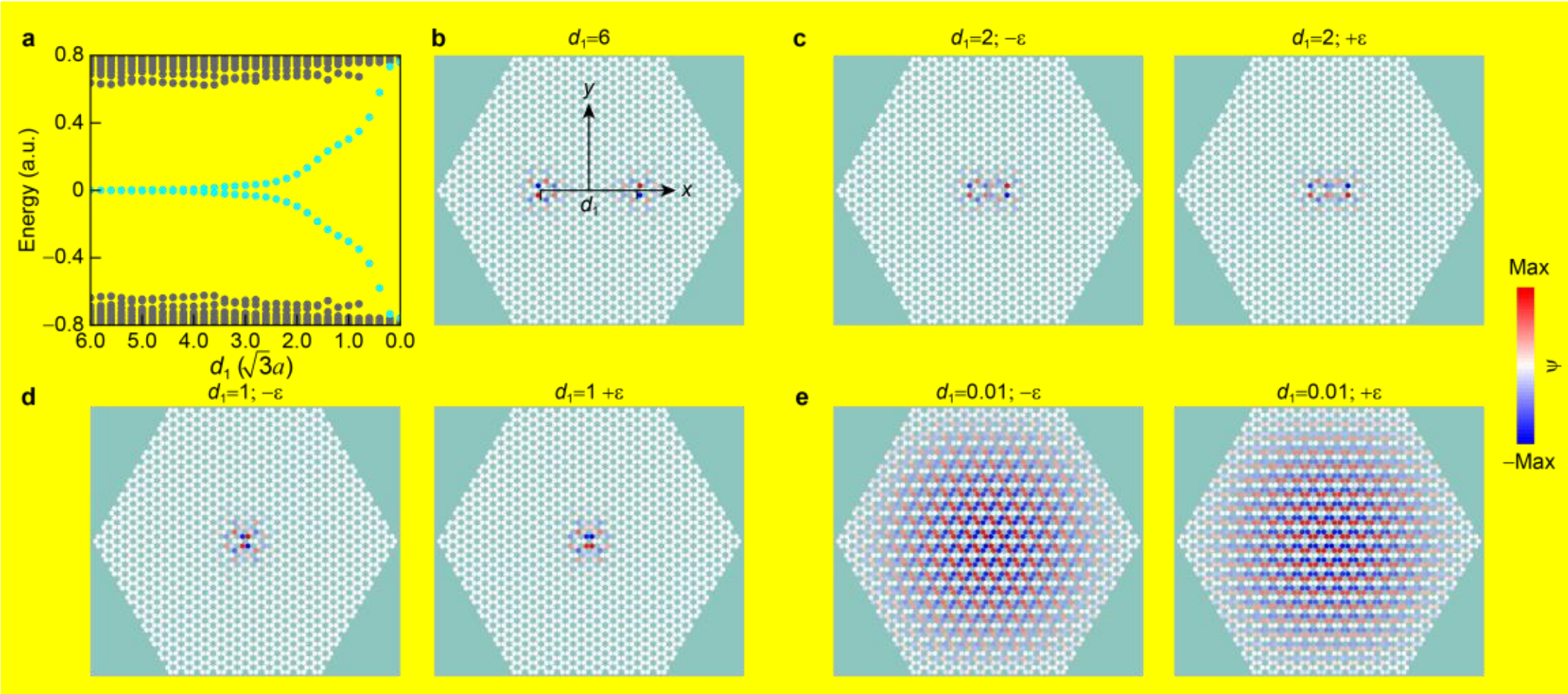


**FIG. S8 | Annihilation of two CMLZMs. a**, Eigenenergy spectrum as a function of the distance $d_1$ between the vortex cores, as marked in **b**. Cyan dots indicate the energies of the two CMLZM-like modes. **b–d**, Eigenfield distributions of the CMLZM modes for different separations: $d_1 = 6\sqrt{3}a$ in **b**, $d_1 = 2\sqrt{3}a$ in **c**, and $d_1 = \sqrt{3}a$ in **d**. **e**, Eigenfield distribution when the vortex centers become very close, with $d_1 = 0.01\sqrt{3}a$. In **c–e**, +ε and −ε label the eigenstates with positive and negative energies, respectively. In the calculations, except for the shape of the PC and the locations of the MLZMs, all other unspecified parameters are the same as those in Fig. S7.

5. The paper did not provide sufficient background in the introduction. There have been significant dveleopments on braiding classical setting in the past few years, e.g., Nat. Photon. 16, 390–395 (2022); Nat. Phys. 18, 179–184 (2022); Nat. Commun. 16, 3650 (2025). There are also those that make use of the properties of exceptional points in non-Hermitian systems, please also survey those briefly.

**Reply:** We thank the referee for this constructive suggestion. We agree that recent progress on braiding in classical wave systems should be more properly introduced. In the revised manuscript, we have expanded the Introduction to include a brief discussion of recent classical realizations of non-Abelian braiding and holonomy in photonic and acoustic systems, with Refs. [30–34] added. In addition, we have acknowledged non-Abelian operations realized by encircling multiple exceptional points, with Refs. [35–38] added. We would also like to emphasize that, our work reports the first direct

observation of the braiding of MLZMs in real space, which is enabled by the discovery of BMLZMs in our system.

To clarify this point, we have revised and added the relevant sentence in the second paragraph of Introduction (lines 36-39), as "In parallel, artificial topological lattices have emerged as versatile platforms for exploring non-Abelian and Majorana-like physics[21-29]. For instance, non-Abelian operations on multiple states have been demonstrated in acoustic and photonic waveguide systems[30-34], as well as through state exchange induced by encircling multiple exceptional points[35-38]. On the other hand, vortex-textured Kekulé-modulated graphene…".

**Reviewer #2 (Remarks to the Author):**

I co-reviewed this manuscript with one of the reviewers who provided the listed reports. This is part of the Nature Communications initiative to facilitate training in peer review and to provide appropriate recognition for Early Career Researchers who co-review manuscripts.

**Reply:** We greatly thank the reviewer and co-reviewer for their time and effort in reviewing our manuscript. We also deeply appreciate the reviewer's helpful comments and suggestions, which have greatly improved our work.

**Reviewer #3 (Remarks to the Author):**

In the article entitled "Direct observation of braiding of Majorana-like zero modes in real space", Ma et al. demonstrated the real-space braiding of Majorana-like zero modes (MLZMs) bounded on boundaries. By modulating the Kekulé modulation phase layer by layer, the MLZMs adiabatically evolve on the surfaces of a three-dimensional phononic crystal. The phase accumulation of MLZMs is captured layer by layer, signifying the braiding process upon exchanging the positions of MLZMs.

Instead of relying on interference between core-localized MLZMs, this work not only offers convenient tuning of MLZM position but also provides a direct real-space visualization of braiding process. Overall, the manuscript is written clearly and logically. I would recommend the publication on Nature Communications if the authors could address the following concerns:

**Reply:** We thank the referee for the high evaluation of our work. We have carefully considered all the concerns raised by the referee and have revised the manuscript and Supplementary Information (SI) accordingly. Detailed point-by-point responses are provided below.

1. In the introduction (line 55), the authors claimed the observation of "characteristic phase accumulation that signifies non-Abelian braiding". However, it is unclear from the results whether non-Abelian characteristics are direct consequences of phase accumulation, especially when only two MLZMs are involved. Did the authors mean the braiding between more MLZMs, i.e., the cases that show the non-Abelian nature [Phys. Rev. Lett. 117, 073901 (2016)]?

**Reply:** We thank the referee for this insightful comment. The referee is correct that the braiding feature of the BMLZMs considered in our system follows the same non-Abelian structure as that discussed in [Phys. Rev. Lett. 117, 073901 (2016)], Ref. [44] of the revised manuscript. This fact can be inferred from Fig. S7, where we consider the annihilation of a CMLZM with a BMLZM by shifting the CMLZM toward the boundary. Conversely, one may also move the BMLZM away from the boundary toward the CMLZM. Once the BMLZM leaves the boundary, it becomes a CMLZM.

Therefore, the non-Abelian characteristics of BMLZMs should be the same as those discussed in Ref. [44].

To clarify this point, we have rewritten the relevant sentence and cited Ref. [44] again in the third paragraph of Introduction (lines 55-58): "By mapping the acoustic-field evolution layer by layer along the evolution direction, we directly visualize the complete trajectory and identify the characteristic phase accumulation that signifies non-Abelian braiding when multiple MLZMs are involved[44]."

2. In Fig. 3, is the single boundary-bound MLZM (BMLZM) already braided with vortex-center-bound MLZM (CMLZM)? It will be helpful to track the evolution of CMLZM, compared to the sole CMLZM in Ref. [Nat. Phys. 16, 989 (2020)].

**Reply:** We thank the referee for this constructive comment. The referee is correct that, in our single-BMLZM setup, when $\alpha_0$ increases from 0 to 2π, the BMLZM is already braided with the CMLZM. As shown in newly added Fig. S11 of the SI, also reproduced below, after the BMLZM rotates clockwise along the sample boundary and returns to its initial position, both the BMLZM and the CMLZM acquire an additional π phase; see the comparison between Figs. S11a and S11m. Here for clarity, we highlight the phase distribution along the boundary and at the center of the CMLZM in the lower left insets.

To clarify this point, we have added a new section, Sec. S-IV, to the SI (lines 328-348), which is also quoted below for reference.

**S-IV. Braiding between a BMLZM and a CMLZM**

For the system considered in Fig. 1 of the main text and Fig. S8, both a CMLZM and a BMLZM are present. As discussed in Fig. 1, when $\alpha_0$ increases from 0 to $2\pi$, the BMLZM acquires a π phase factor. In fact, during this process, the CMLZM also acquires an additional π phase. To illustrate this point, Fig. S11 displays the eigenfield distributions of the BMLZM and CMLZM at different values of $\alpha_0$. To highlight the phase accumulation during this process, the lower-left insets show the phase distribution along the boundary and at the center of the CMLZM. Here, the red and blue

disks denote the BMLZM with phases 0 and π, respectively, the hexamers denote the CMLZM, and the colored arrows indicate the evolution direction of the BMLZM. All other parameters are the same as those in Fig. 1. Clearly, as $\alpha_0$ increases from 0 to 2π, the BMLZM rotates clockwise along the boundary, eventually returns to its initial position, and acquires an additional π phase. Meanwhile, the CMLZM also acquires an additional π phase, as shown by comparing Figs. S11a and S11m.

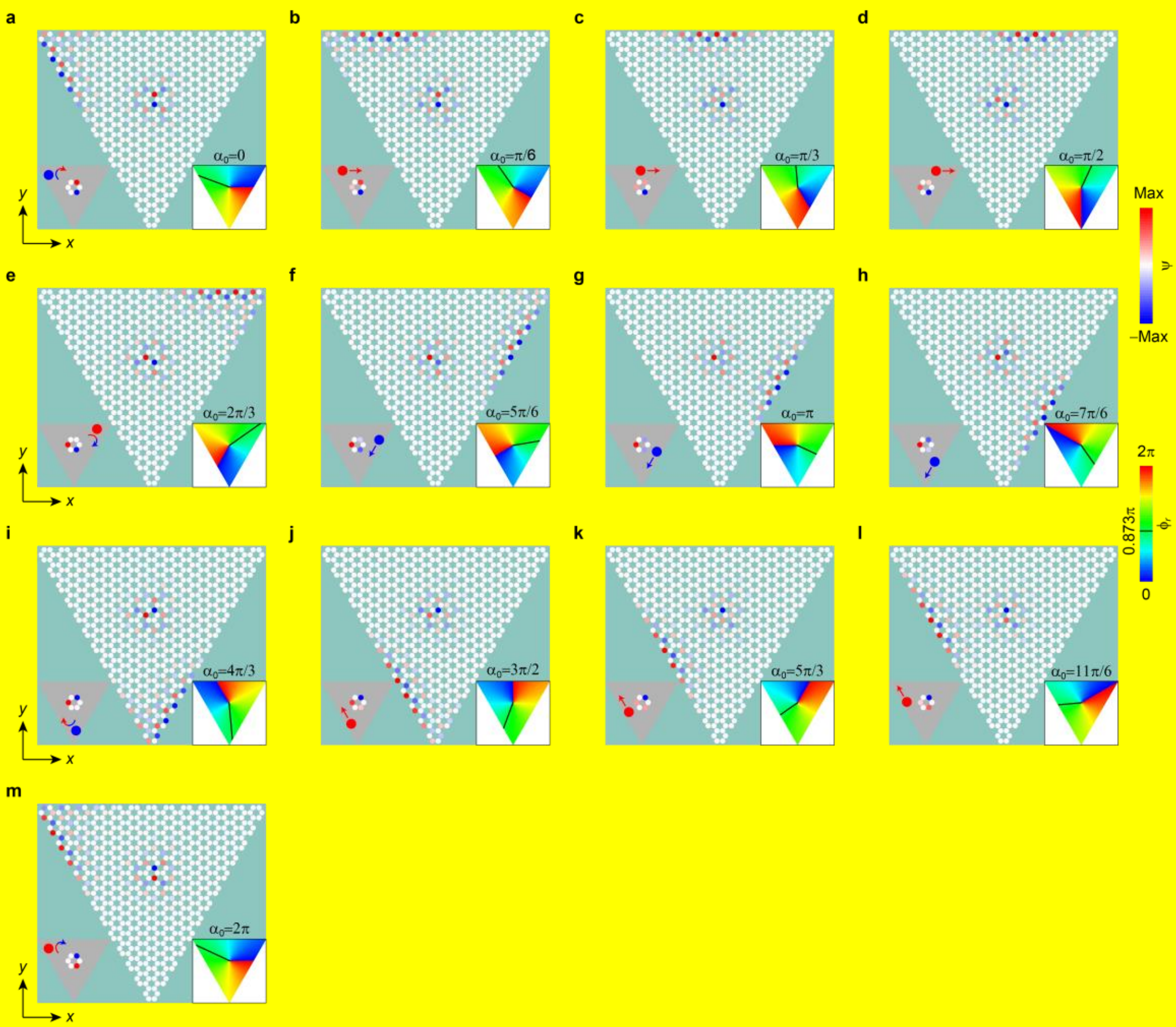


**FIG. S11. | BMLZM braided with the CMLZM as $\alpha_0$ is gradually increased. a-m,** Eigenfield distributions of the BMLZM together with that of the CMLZM for $\alpha_0 = 0$ (**a**), $\pi/6$ (**b**), $\pi/3$ (**c**), $\pi/2$ (**d**), $2\pi/3$ (**e**), $5\pi/6$ (**f**), $\pi$ (**g**), $7\pi/6$ (**h**), $4\pi/3$ (**i**), $3\pi/2$ (**j**), $5\pi/3$ (**k**) $11\pi/6$ (**l**), and $2\pi$ (**m**), respectively. The lower-left insets depict the locations of the BMLZM and the phase distribution along the boundary and at the center of the CMLZM, while the colored arrows indicate the evolution direction of the BMLZM as $\alpha_0$ increases. The lower-right insets show the distributions of the modulation phase $\phi_r$, and the black lines mark the position with $\phi_r = 0.873\pi$.

Except for $\alpha_0$, all the other parameters are the same as Fig. 1 of the main text.

3. In Fig. 4, the intensity profile of BMLZM2 looks much weaker than BMLZM1, making its localization hard to see from the experimental result. Its frequency spectrum also shows unclear peak at the zero-mode frequency (Fig. S17). Could the authors explain why?

**Reply:** We thank the referee for the critical comment. In Fig. 4, also reproduced below as Fig. R2, we design a PC that supports two BMLZMs simultaneously. The distribution of the modulation phase $\phi_r$ is designed such that the two BMLZMs are initially located on the upper boundary. After adiabatically increasing the modulation phase as $\phi_r \to \phi_r + 2\pi$, the two BMLZMs rotate clockwise simultaneously and exchange their positions on the top layer. Figure R2a sketches the braiding process. It is clear that the BMLZM located on the right-hand side of the 1st layer travels a much longer distance than the BMLZM located on the left-hand side during this process. Due to unavoidable experimental loss, the signal of the BMLZM that propagates over the longer distance is expected to be weaker than that of the BMLZM that propagates over the shorter distance. That is why the intensity profile of BMLZM2 looks much weaker than BMLZM1.

In fact, the insets in Figs. R2c and R2h show the normalized measured signals near each BMLZM. From these normalized signals, we can still clearly observe that the two BMLZMs are in phase on the 1st layer and out of phase on the 13th layer.

To clarify this point, we have added two subfigures, Figs. R2a and R2b (Figs. 4a and 4b), which clearly illustrate the different propagation distances. In addition, we have added a new sentence at the end of the caption to explain the contrast in the measured signal (lines 377-379), as "Due to unavoidable experimental loss, the signal of the BMLZM on the right-hand side of the first layer, or equivalently on the left-hand side of the 13th layer, propagates over a longer distance and is therefore much weaker than that of the BMLZM on the opposite side."

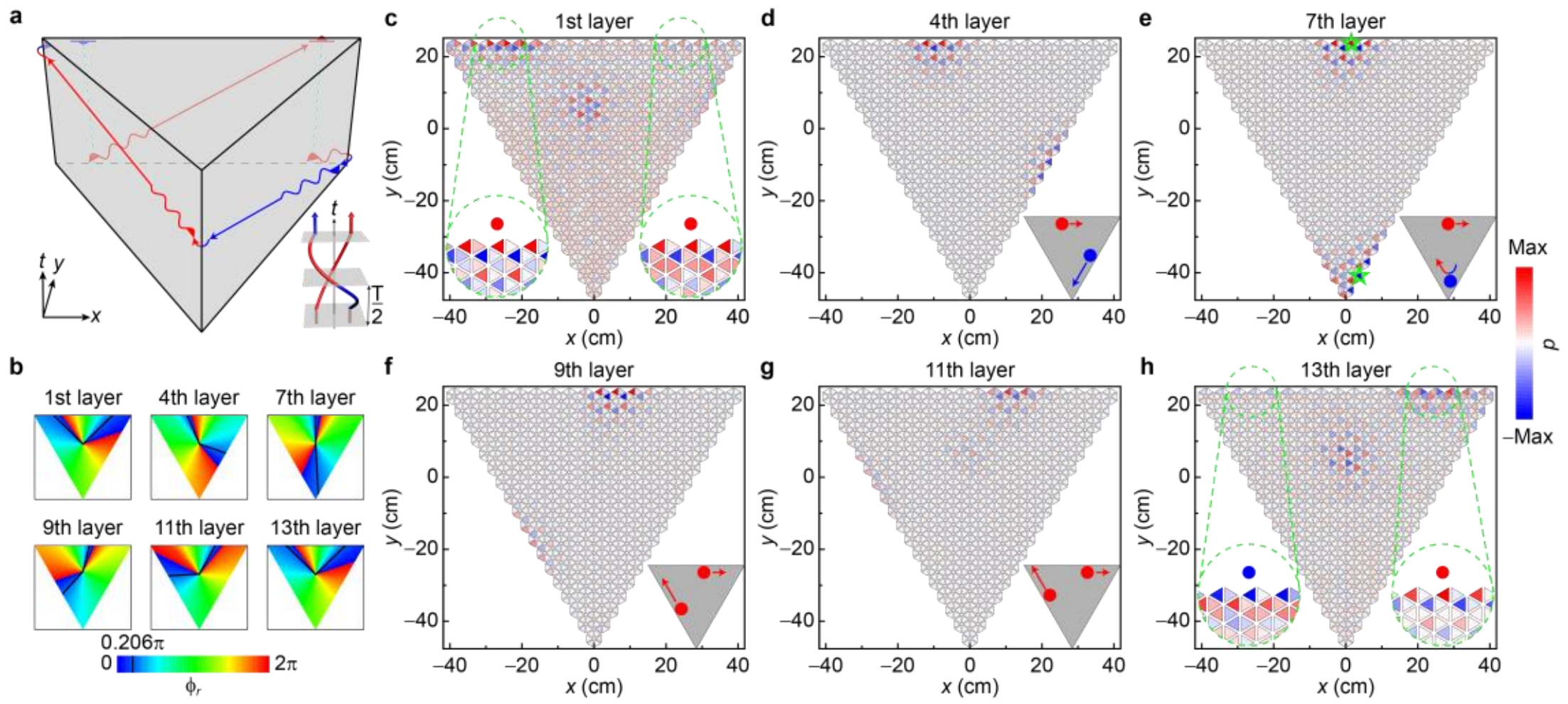

**Fig. R2. Braiding of two BMLZMs in a 3D PC.** **a**, Braiding trajectory of the two BMLZMs in a triangular-prism space ($x$-$y$-$t$). The inset illustrates the schematic of the braiding process, with $T$ representing the period for the BMLZMs to rotate back to the initial configuration when the modulation phase $\phi_r \to \phi_r + 2\pi$. **b**, The distributions of the modulation phase $\phi_r$ for six representative layers (layers 1, 4, 7, 9, 11, and 13), corresponding to $\alpha_0 = 0$, $\pi/2$, $\pi$, $4\pi/3$, $5\pi/3$, and $2\pi$. The black lines mark the position with $\phi_r = 0.206\pi$. **c-f**, Measured field distributions of the BMLZMs on six representative layers, respectively. The lower-right insets mark the positions of the BMLZMs, with the colored arrows indicating their evolution directions as the layer number increases. The insets in **c** and **h** present enlarged views of the normalized field distributions of the two BMLZMs, showing that they are in phase in **c** and out of phase in **h**. The green stars in **e** mark the positions of the sound sources operating at 9.96 kHz. $N_b = 18$ and other geometric parameters of the PC are provided in Supplementary Note IX. Due to unavoidable experimental loss, the signal of the BMLZM on the right-hand side of the first layer, or equivalently on the left-hand side of the 13th layer, propagates over a longer distance and is therefore much weaker than that of the BMLZM on the opposite side.

4. About the Jackiw-Rebbi formalism for BMLZM. Which symmetry protects the quantized Zak phase of topological boundary bands? Such topological transition of boundary bands can be related to embedded topology [Phys. Rev. B 100, 115126 (2019);

Phys. Rev. Lett. 135, 136602 (2025)].

**Reply:** We thank the referee for this insightful comment. As shown in Fig. S5(a) of the SI, the ribbon geometry of the lattice with uniform Kekulé modulation possesses mirror symmetry along the *x* direction. As established in Ref. [J. Phys.: Condens. Matter 12, R107 (2000)], in a one-dimensional system with mirror symmetry, the Zak phase of each isolated band is quantized. Therefore, the Zak phase of the topological boundary band in our system is also quantized. The topological phase transition of the boundary bands occurs when the two boundary bands form a one-dimensional Dirac dispersion at $\phi = \pm 0.873\pi$.

We also note that this system possesses chiral symmetry. However, chiral symmetry is not the symmetry responsible for protecting the quantized Zak phase in this case. This can be verified by adding next-nearest-neighbor coupling, which breaks chiral symmetry by introducing same-sublattice coupling, while the Zak phase remains quantized as long as mirror symmetry is preserved.

The referee correctly points out that the two-dimensional bulk with uniform Kekulé modulation is topologically trivial, whereas the boundary bands can acquire a nontrivial Zak phase after the topological transition at $\phi = \pm 0.873\pi$. Therefore, according to the definition in Ref. [Phys. Rev. B 100, 115126 (2019)], this system can indeed be understood as an example of embedded topology. However, in our system, the topology of the boundary bands is protected by mirror symmetry, rather than by the combined projected symmetry discussed in Ref. [Phys. Rev. Lett. 135, 136602 (2025)].

To clarify this point, we have added one sentence in the part S-II of the revised SI explicitly stating that the quantization of the Zak phase is protected by mirror symmetry along the *x* direction (lines 140-141), as "…and here is quantization of Zak phase is ensured by the mirror symmetry along the *x* direction".

5. Continuing from point 4, the authors also directly relate the Jackiw-Rebbi formalism to the phononic crystal. To be clear, could the authors verify whether the phononic crystal hosts the topological boundary bands?

**Reply:** We thank the referee for the constructive suggestion. To verify the topological

origin of BMLZM in PC, we calculate the topological boundary bands and the corresponding Zak phase. The results and relevant discussions are provided in SI as part of S-VI (lines 3945-411), also quoted below for your reference.

To elucidate the topological origin of BMLZM in PC, we also consider a ribbon lattice which is periodic in the $x$ direction and large enough in the $y$ direction, as illustrated in Fig. S14a. This ribbon lattice is subjected to the uniform-texture Kekulé modulation phase $\phi$, which depends on the initial offset $\alpha_0$. The outer normal of the upper armchair edge boundary is also parallel to $\Gamma K_+$ (the same as the boundaries in Fig. 1 of main text), while that of the lower armchair edge boundary is parallel to $\Gamma K_-$. Figures S14b and S14c show the projected bands of the ribbon PC for $k_x$ with $\phi = 0.973\pi$ and $\phi = 0.773\pi$, respectively. Here the dark solid red (gray) lines represent the dispersion of the boundary modes localized on the upper (lower) boundary. These two $\phi$ values are purposely chosen to be on different sides of the Dirac point of the upper boundary states. The Zak phases of the boundary bands are also calculated and labelled, revealing a topological phase transition (0, 0) to (π, π) for the boundary bands localized on the upper boundary from, which is linked to the well-known Jackiw-Rebbi mechanism.

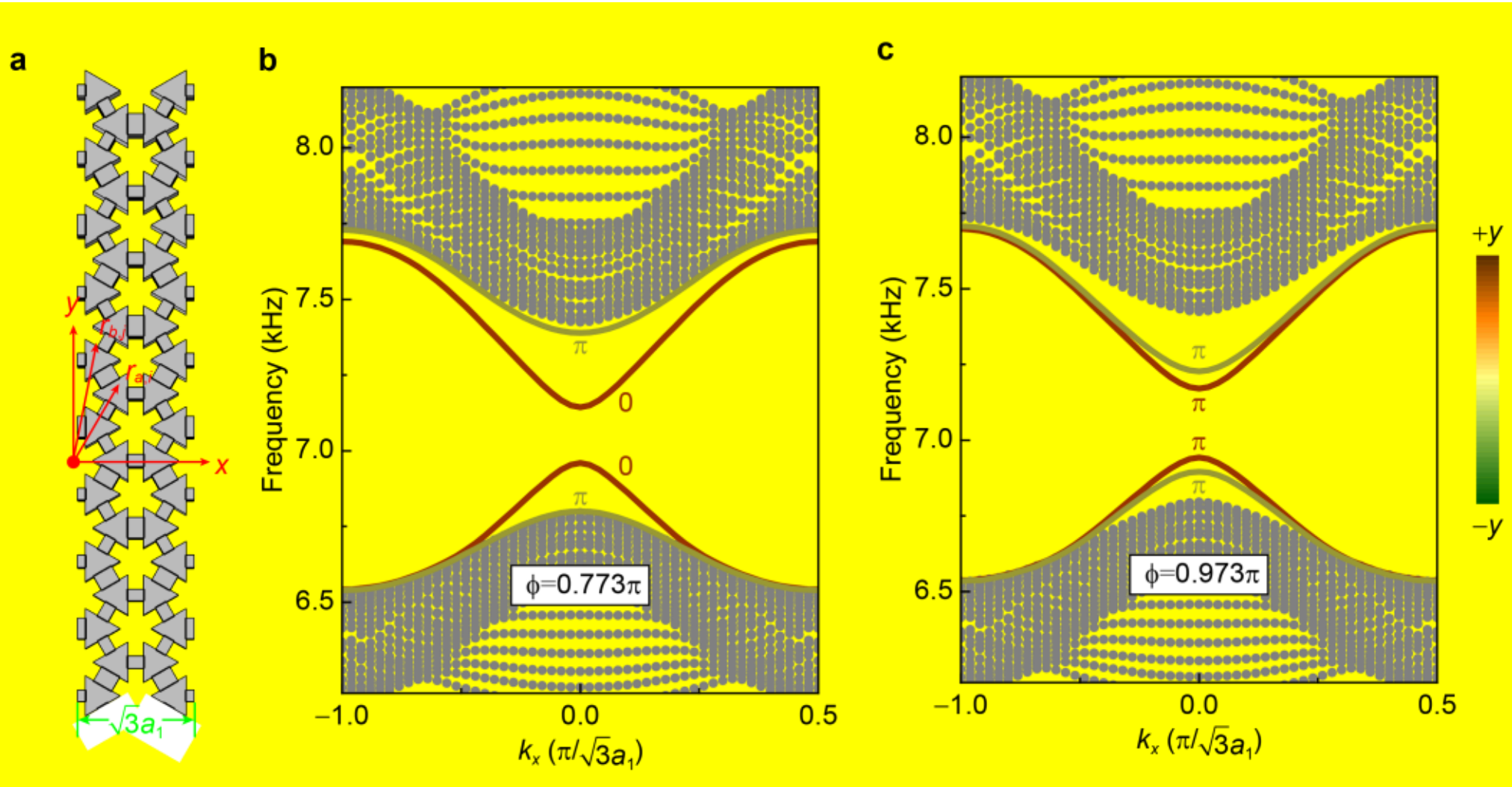


**Fig. S14. | Topological phase transition of the boundary bands in PC. a**, A ribbon geometry of PCs with uniform Kekulé modulation denoted by $\phi$. **b**, **c**, Projected band structures of the ribbon versus $k_x$ at $\phi = 0.773\pi$ (**b**) and $\phi = 0.973\pi$ (**c**), where the Zak phases of the boundary modes are also given. The dark red (gray) lines indicate

the states at upper (lower) boundary and dots denote bulk states. In calculation, the periodic boundary conditions are applied in the $x$ direction, while the ribbon structure is thick enough in the $y$ direction (the number of units is 20).

6. For a thorough survey on MLZM applications, the authors may consider citing its recent lasing realizations in photonics [Nat. Commun. 14, 707 (2023); Light Sci. Appl. 12, 255 (2023)].

**Reply:** We thank the referee for bringing us these two interesting works and this constructive suggestion. Indeed, they are related to our work and they have now been cited as Refs. [23] and [24].

I also suggest the authors take note of these comments for smoother reading:

1. In Figs. 2-3, are the units of x, y axes in the field mapping consistent with the experimental setup?

**Reply:** We appreciate the referee for pointing out these typos. In the revised manuscript, the units of x, y axes in Fig. 3 have been revised.

2. To differentiate between Figs. 3-4, it will be helpful to include the Kekulé phase map and braiding configuration of BMLZMs in Fig. 4, where multiple zero modes are involved.

**Reply:** We thank the referee for the valuable suggestion. The Kekulé phase map and braiding configuration of BMLZMs have now been added in Fig. 4.

Related descriptions have also been added in the caption of Fig. 4 and main text of the revised manuscript (lines 366-371 and 174-180) , as "**a**, Braiding trajectory of the two BMLZMs in a triangular-prism space ($x$-$y$-$t$). The inset illustrates the schematic of the braiding process, with $T$ representing the period for the BMLZMs to rotate back to the initial configuration when the modulation phase $\phi_r \to \phi_r + 2\pi$. **b**, The distributions of the modulation phase $\phi_r$ for six representative layers (layers 1, 4, 7, 9, 11, and 13), corresponding to $\alpha_0 = 0,\ \pi/2,\ \pi,\ 4\pi/3,\ 5\pi/3$, and $2\pi$. The black lines mark the position with $\phi_r = 0.206\pi$." and "The schematic of the braiding of two

BMLZMs around another two CMLZMs is illustrated in Fig. 4a. … Figure 4b shows the distributions of the modulation phase $\phi_r$ for six layers (layers 1, 4, 7, 9, 11, and 13), corresponding to $\alpha_0 = 0,\ \pi/2,\ \pi,\ 4\pi/3,\ 5\pi/3$, and $2\pi$. The black lines mark the position with $\phi_r = 0.206\pi$ where the BMLZMs are located at (see Supplementary Note II). ”

3. In the analytical solution of BMLZM [Eq. (S7)], what do x_0 and y_0 represent?

**Reply:** We appreciate the referee for pointing out this mistake. In Eq. (S7) and the following text of the revised SI, $x_0$ ($y_0$) represents the coordinate of the domain-wall (boundary in $y$ direction), as shown in Fig. R3.

Related descriptions have been added in the **S-II. c** of revised SI and $x_0$ and $y_0$ have also been labelled in the Fig. S6a as "$x_0$ ($y_0$) represents the position of the domain-wall (the upper boundary in the $y$ direction), as labeled in Fig. S6a."

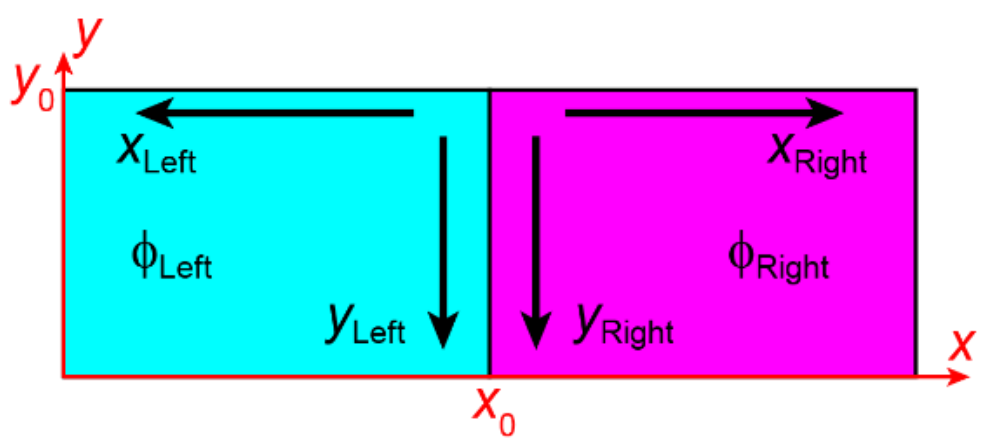


**FIG. R3.** Sketch showing the sampling points location and two domains.
